# Endoplasmic reticulum engagement of transcripts triggers *VSG* mRNA balancing in trypanosomes

**DOI:** 10.1371/journal.ppat.1014518

**Published:** 2026-08-18

**Authors:** Erick O. Aroko, Elisabeth Meyer-Natus, Christopher Batram, Kathrin Weißenberg, Jasmin Henning, Majeed Bakari-Soale, Nicola G. Jones, Markus Engstler

**Affiliations:** 1 Department of Cell and Developmental Biology, University of Würzburg, Würzburg, Germany; 2 Department of Medical Biochemistry, Kisii University, Kisii, Kenya; Heidelberg University, GERMANY

## Abstract

The cell surface of bloodstream-form African trypanosomes is covered by a dense coat of variant surface glycoproteins (VSGs). By periodically switching the expressed VSG antigen, parasites evade host immune responses. *VSG* mRNA constitutes ~10% of total cellular mRNA, and depletion of *VSG* transcripts is lethal. When two VSGs are expressed simultaneously, however, total *VSG* mRNA levels remain close to wild-type amounts, indicating the presence of a balancing mechanism that limits the overall abundance of these highly expressed transcripts. Using inducible and constitutive expression systems, we found that attenuation of endogenous *VSG* mRNA requires efficient engagement of ectopic transcripts with the endoplasmic reticulum (ER). This response occurs independently of efficient VSG protein production and does not require a *VSG* open reading frame. In contrast, abundant transcripts lacking functional ER-engagement signals fail to trigger balancing despite containing the *VSG* 3′ UTR 16-mer stability element. These results indicate that the signal for *VSG* mRNA regulation is the presence of abundant ER-engaged transcripts (i.e., transcripts undergoing co-translational targeting to the ER), rather than VSG-specific sequence features or productive protein synthesis. Based on our findings together with previous work, we propose an ER-engagement-coupled homeostatic attenuation mechanism in which increased ER-engaged transcript load elicits a regulatory response that reduces endogenous *VSG* mRNA abundance. This model links the cytoplasmic burden of ER-targeted transcripts to transcriptional control of the nuclear VSG expression site, providing a mechanism that could allow trypanosomes to couple secretory pathway capacity to surface antigen expression. Such a mechanism could enable trypanosomes to maintain secretory pathway homeostasis while supporting rapid surface-coat remodelling during antigenic variation.

## Author summary

African trypanosomes evade the host immune system by regularly changing the variant surface glycoprotein (VSG) that coats their cell surface. At any given time, one VSG is expressed at very high levels, and the parasite tightly controls the total amount of *VSG* mRNA. Previous work showed that when an additional VSG is artificially expressed, the parasite compensates by reducing expression of the original VSG, a phenomenon termed “mRNA balancing.” However, the signal that triggers this response has remained elusive. Here, we show that mRNA balancing does not require production of a fully functional VSG protein. Instead, the response is triggered when large amounts of *VSG* mRNA, or other mRNAs encoding ER-targeted proteins, engage the endoplasmic reticulum (ER), the cellular compartment where secreted and surface proteins enter the secretory pathway. Our findings support a model in which *VSG* mRNA balancing is triggered by the abundance of ER-engaged mRNAs, rather than by successful assembly of a functional surface coat. This could provide a rapid way to adjust VSG expression and may help the parasite maintain surface homeostasis during immune evasion.

## Introduction

African trypanosomes parasitise humans and a wide range of mammals, causing sleeping sickness and Nagana, respectively. These parasites have a digenetic life cycle, shuttling between the mammalian hosts and their insect vector, the tsetse fly. The mammalian stage bloodstream form (BSF) parasites are extracellular, residing in the blood, lymph, and the interstitial fluid of various tissues. Therefore, African trypanosomes are under constant attack from the host’s immune system, and their cell surface has evolved as the first and most important line of defence. The cell surface of BSF *Trypanosoma brucei* is the best characterised compared to other African trypanosomes. This parasite is coated by a dense monolayer of an immunogenic glycosylphosphatidylinositol (GPI) anchored protein known as the variant surface glycoprotein (VSG). Other than providing an impenetrable protective shield to the parasites, the VSG coat is essential for antibody clearance and antigenic variation [1–3]. Together, these mechanisms ensure that the parasites stay ahead of the host’s immune responses.

As GPI-anchored proteins, nascent VSGs contain an N-terminal endoplasmic reticulum (ER) import signal peptide that is cleaved following translocation to produce the mature protein [4,5]. Although no strict consensus sequence has been defined for these naturally occurring ER import signals, cleavable signal peptides of secretory and GPI-anchored proteins share several common features. These include a positively charged N-terminal region (n-region), a hydrophobic core (h-region), and a polar C-terminal region (c-region) [6,7]. The c-region contains the signal peptidase cleavage site and typically harbours small, uncharged aliphatic residues at the -1 and -3 positions relative to the scissile bond [8,9]. Variations in the signal peptide sequence affect different aspects of protein biogenesis, including targeting and translocation, cleavage, and ER exit [6,10], and the hydrophobicity of the h-region has been reported to be vital in determining whether a substrate is translocated by the signal receptor particle (SRP) dependent or independent pathway in yeast [11].

Further, both the length and overall hydrophobicity of the h-region appear to be critical for efficient translocation [15]. *T. brucei* VSG ER import signal peptides are approximately 15–30 residues long and share the characteristic features for signal peptides in yeast and mammals [5,16,17].

Transcription of virtually all eukaryotic genes is monocistronic. However, most *T. brucei* protein-coding genes are arranged in polycistronic arrays transcribed by RNA polymerase II [18,19]. The long polycistronic transcripts are processed into mature mRNAs by trans-splicing of a 39-nucleotide spliced leader to the 5′ end and polyadenylation at the 3′ end of individual genes [20–22]. Due to this polycistronic arrangement of genes and a lack of transcriptional initiation control of individual genes, regulation of RNA levels in *T. brucei* is predominantly post-transcriptional [23]. RNA-binding proteins (RBPs) and sequence elements within the 3′ UTRs of genes are crucial for this post-transcriptional modulation of *T. brucei* gene expression [24,25]. Transcription of the major surface proteins in *T. brucei* is unusual in that it is driven by RNA polymerase I. In the BSF, a single *VSG* gene located in one of ~15 telomeric polycistronic VSG expression units known as the expression site (ES), is monoallelically expressed to ensure that only a single VSG is produced at a time from the vast repertoire of 2000 genes and pseudogenes [26,27]. The active ES localises to the extranucleolar expression site body (ESB) in the nucleus [28,29].

The expression of VSG is tightly regulated to ensure an intact coat is maintained both *in vivo* and *in vitro*. Down-regulation of VSG expression via RNAi or by blocking translation using morpholino oligonucleotides causes a precise pre-cytokinesis cell cycle arrest and subsequent cell death [30,31]. A study by Muñoz-Jordán et al. [32] showed *T. brucei* could be engineered to constitutively express two VSGs in equal amounts by integrating a second *VSG* gene just upstream of the active ES-resident *VSG*, generating a so-called double expressor. Integration of a second *VSG* gene downstream of the active ES promoter, approximately 60 kb upstream of the native *VSG*, also supported stable expression of two VSGs with roughly equal amounts of both mRNA and protein, that did not exceed the wild-type levels. In addition, when transcripts of one of the *VSGs* were depleted using RNAi, there was a compensatory effect by which the transcripts of the other *VSG* were upregulated to wild-type levels [33]. A similar apparent upregulation of the ectopic *VSG* expression was observed when the native ES-resident *VSG* was deleted by replacement with a blasticidin resistance gene [34]. *VSG* mRNA regulation has been shown not to require expression site residence. Inducible overexpression of an ectopic *VSG121* from the ribosomal spacer region resulted in a fast and efficient exchange of the *VSG* mRNA population and protein, with similar kinetics in both monomorphic and pleomorphic trypanosomes [35,36]. In the monomorphic cell line, the ectopic *VSG* mRNA is rapidly upregulated to 80% of the wild-type levels within 2 h of inducing expression and this level of expression is maintained for at least 24 h. Concomitantly, the endogenous *VSG* mRNA is downregulated and reaches ⁓25% of wild-type amounts within 6 h of induction of expression [35]. Taken together, these observations strongly suggest the existence of a regulatory mechanism that maintains *VSG* mRNA abundance within a narrow range. However, the molecular trigger for this balancing response remains unknown.

One candidate signal that could be involved in *VSG* mRNA control is a conserved 16-mer sequence present in all *T. brucei VSG* 3′ UTRs that is essential for high expression and stability of *VSG* mRNA [37]. This sequence has been proposed to recruit a limiting RNA-binding factor that could potentially act as a counting mechanism [34]. Recently, it has been suggested that this limiting factor could be the 16-mer motif-dependent inclusion of N^6^-methyladenosine in the *VSG* poly(A) tails [38]. In another study the mRNA binding protein CFB2 was found to interact with the 16-mer motif, where it mediates *VSG* mRNA stability by recruitment of a stabilising complex. In addition, it was proposed that regulation of CFB2 levels might in turn limit VSG synthesis to avoid excess production [39]. However, by introducing premature termination codons (PTCs) in the ectopic *VSG* gene open reading frame (ORF), Maudlin et al. reported massively increased total *VSG* mRNA levels, thus questioning whether a direct counting mechanism for *VSG* mRNA exists [40].

Here, we show that the endogenous *VSG* mRNA regulation response is elicited only when ectopic transcripts are targeted to the ER. Consistent with this interpretation, high-level expression of ER-targeted GFP is sufficient to trigger attenuation of endogenous *VSG* mRNA. These findings indicate that the balancing response does not depend on the identity of the *VSG* open reading frame or efficient VSG protein production but instead correlates with the presence of abundant ER-engaged transcripts. We therefore propose that trypanosomes maintain secretory pathway homeostasis by attenuating endogenous *VSG* mRNA when the load of ER-engaged transcripts increases.

Thus, the counterbalancing of *VSG* mRNA levels appears to be a strategy for regulating the secretory pathway’s cargo and maintaining ER homeostasis. As the regulation is independent of the open reading frame, it could well facilitate the rapid exchange of surface coats during antigenic variation and developmental progression.

## Results

### Different *T. vivax* VSGs elicit distinct *VSG* mRNA balancing responses in *T. brucei*

The abundance of *VSG* mRNA in *T. brucei* is tightly regulated, and previous studies have suggested that sequence elements within the *VSG* transcript might contribute to this balancing mechanism. We therefore asked whether *T. vivax VSG* transcripts can trigger the *VSG* mRNA balancing response in *T. brucei*.

For this purpose, we selected two well documented *T. vivax* VSGs, *ILDat1.2* (TriTrypDB: TvY486_0008160) and *ILDat2.1* (GenBank: Z48228.1) [41–45], for inducible overexpression in *T. brucei*. This expression strategy was used because it enables monitoring of early events after induction of VSG expression, and it is not obscured by adaptive responses to constitutive ectopic VSG expression, which can occur during selection of transgenic parasites [35,46]. First, a construct for the expression of ILDat2.1 VSG was generated.

**Fig 1:**
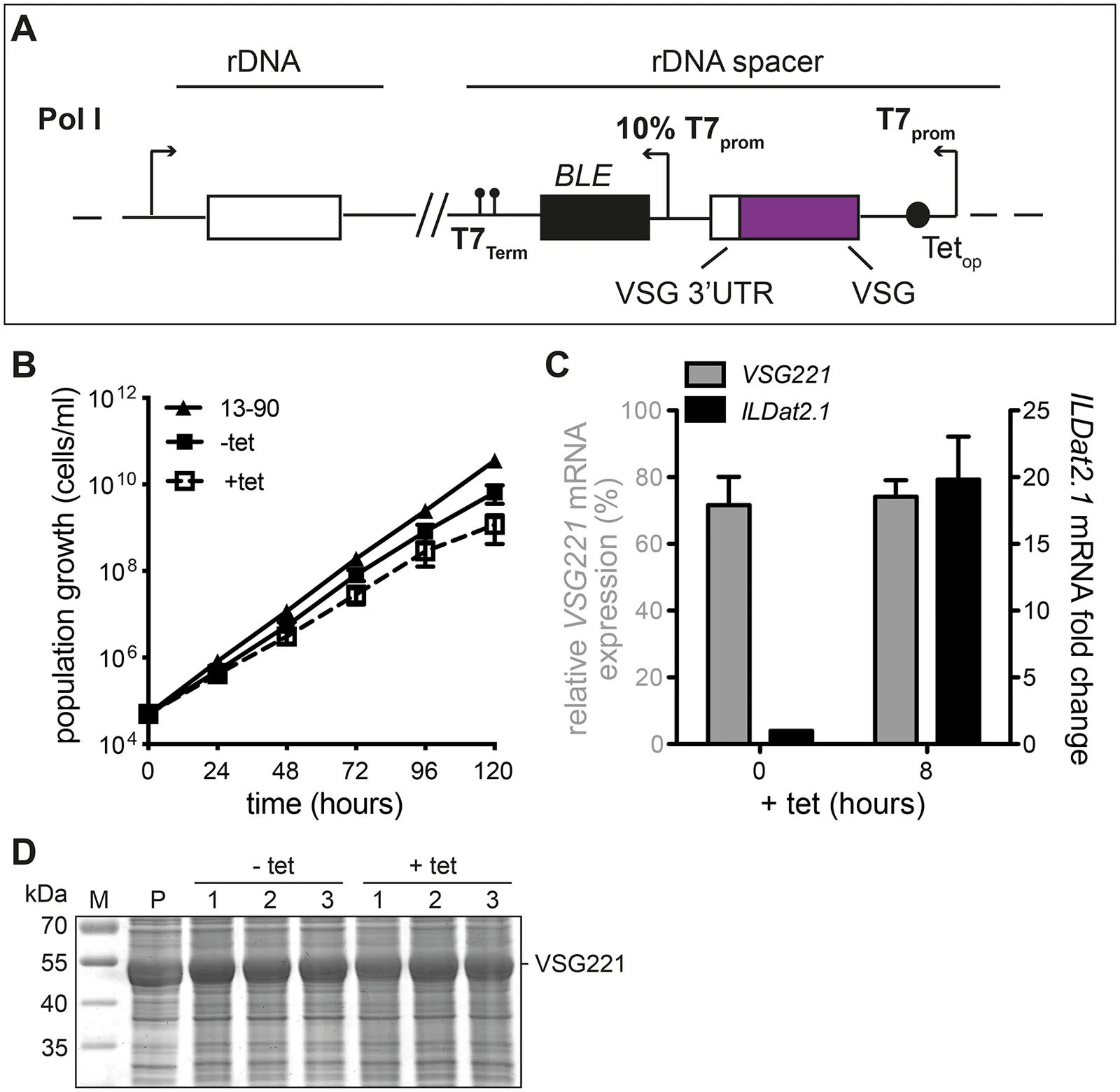
An endogenous *VSG* mRNA regulation response is not elicited upon induction of ILDat2.1 expression. **(A)** Schematic of the ectopic VSG overexpression strategy. The *ILDat2.1* construct was integrated into a transcriptionally silent rDNA spacer and expression of the VSG was driven by the T7 promoter in the presence of tetracycline. **(B)** Cumulative growth profiles of uninduced (-tet) and induced (+tet) 221^ES^.ILDat2.1^tet^ cells. The data are averages from three independent clonal cell lines, with error bars representing ± standard deviation (SD). The parental 13-90 cells (P) served as a growth control. **(C)** Relative quantification of *VSG* mRNA levels in the presence (8 h) or absence (0 h) of tetracycline. Expression of *VSG221* (left y-axis) is presented as a percentage relative to the *VSG221* expression of the parental 13-90 cells while the ectopic *ILDat2.1* value (right y-axis) is relative to the non-induced levels. The values are given as mean ± SD of three independent clones. The mRNA was normalised to *tubulin*. **(D)** Analysis of ILDat2.1 protein expression by SDS-PAGE after 24 h in the presence (+tet) or absence (-tet) of tetracycline. The parental 13-90 cells (P) served as the loading control and three independent clones (1 – 3) were analysed.

As the published *ILDat2.1* sequence lacks a start codon [44], we added a start codon to the *ILDat2.1* VSG open reading frame (S1 Table). Given that the conserved elements in the *VSG* 3′ UTRs are unique to *T. brucei* and essential for high levels of expression [34,37], a *T. brucei VSG* 3′ UTR sequence was fused to the *ILDat2.1 VSG* ORF, which was then integrated into the transcriptionally silent rDNA spacer region using the pLew82v4 vector (Addgene plasmid #24009). Expression of the ectopic *VSG* genes was driven by an ectopic tetracycline inducible T7 RNA polymerase promoter (Fig 1A) [35].

Inducing the expression of ILDat2.1 VSG in the 221^ES^.ILDat2.1^tet^ cell line caused a minor reduction in proliferation (Fig 1B). We tested whether high levels of *ILDat2.1 VSG* mRNA were transcribed and if so, whether an endogenous *VSG* mRNA regulation response was triggered upon induction of expression. Quantitative dot blots carried out 8 h after induction showed an approximately 20-fold increase of the *ILDat2.1 VSG* mRNA. This increase was higher than the approximate 10-fold change recorded when wild-type *T. brucei VSG*s are expressed in this way. The endogenous *VSG221* mRNA level was, however, not downregulated (Fig 1C). This contrasts with the regulation that occurs when *T. brucei VSG*s are overexpressed. No ILDat2.1 VSG protein was detectable by Coomassie staining, although low-level expression cannot be excluded (Fig 1D). As the endogenous *VSG221* mRNA was not affected, and thus VSG221 protein levels remained high, the transgenic trypanosomes grew normally (Fig 1B-1D).

Despite strong accumulation of *ILDat2.1* transcripts, endogenous *VSG221* mRNA levels remained unchanged. Thus, high levels of a 16-mer–containing *VSG* transcript alone are insufficient to trigger *VSG* mRNA balancing.

**Fig 2:**
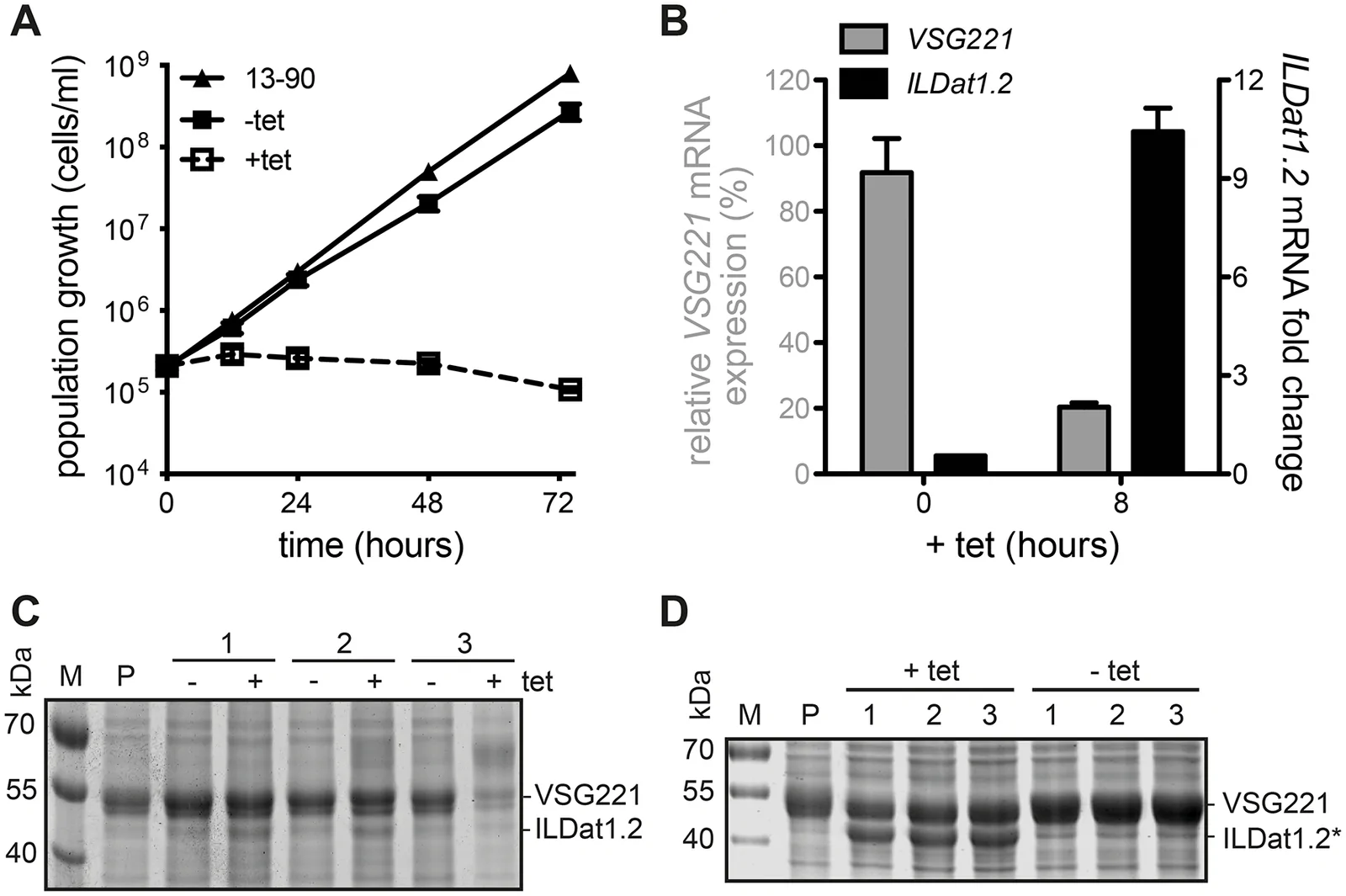
The endogenous regulation response is elicited upon induction of ILDat1.2 VSG expression. **(A)** Cumulative growth of the 221^ES^.ILDat1.2^tet^ cell line in the presence (+tet) or absence (-tet) of tetracycline. The data are averages of three independent clones and the error bars represent ± SD. The parental 13-90 cells (P) served as a growth control. **(B)** Relative quantification of mRNA levels of the ectopic *ILDat1.2* expressed from an rDNA spacer and the endogenous *VSG221* using dot blots. The cells were induced to express the ectopic *VSG* for 8 h**.** Expression of *VSG221* (left y-axis) is given as a percentage relative to the *VSG221* expression of the parental 13‑90 cells while the ectopic *ILDat1.2* value (right y-axis) is relative to the non-induced levels. *VSG* transcript levels were normalised to *tubulin* and the results given as means of three independent clones. The error bars indicate the SD. **(C, D)** SDS-PAGE analyses of protein expression of wild-type ILDat1.2 **(C)** and ILDat1.2 with its GPI signal sequence replaced by that of the *T. brucei* VSG MITat1.11 (ILDat1.2*) **(D)** after 24 h in the presence (+tet) or absence (-tet) of tetracycline. The parental 13-90 cells (P) served as a reference. Three independent inducible clonal cell lines were analysed.

Induction of expression of a second *T. vivax* VSG, ILDat1.2 VSG (S1 Table), in the 221^ES^.ILDat1.2^tet^ cell line, led to a different phenotype, namely a significant slowing in growth within 24 h and complete stalling thereafter (Fig 2A). Quantitative dot blots carried out 8 h after inducing expression showed that a high amount of *ILDat1.2 VSG* mRNA was present, with a concomitant reduction of the endogenous *VSG221* mRNA to 20% of the wild-type levels (Fig 2B). This phenotype contrasted with that observed for VSG ILDat2.1; however, the reason for this difference was difficult to interpret. Furthermore, the analysis of ILDat1.2 expression showed that the protein was produced in only low amounts in two of the three clones analysed (Fig 2C), differing from what happens when *T. brucei* VSGs are overexpressed. This means that downregulation of the endogenous *VSG* mRNA, in addition to the insufficient production of the ectopic ILDat1.2, resulted in a shortage of VSG protein and slowed parasite growth, which eventually led to cell death.

In search for the cause of the poor production of ILDat1.2 VSG protein, we examined whether the native ILDat1.2 GPI-anchoring signal functions efficiently in *T. brucei*. We replaced the C-terminal GPI-signal sequence with that of *T. brucei* VSG MITat1.11 (S1 Table). This ILDat1.2 VSG transgene was highly expressed on both mRNA and protein levels (Fig 2D), and cell growth was normal (S1 Fig).

These results demonstrate that, in principle, a *T. vivax VSG* transcript fused to a *T. brucei VSG* 3′ UTR can elicit *VSG* mRNA balancing in *T. brucei*. However, the balancing response was only observed in the ILDat1.2 overexpression cell line but not in the ILDat2.1 overexpression cell line. This discrepancy prompted us to investigate which feature of the ectopic transcript determines whether *VSG* mRNA balancing is triggered.

### Functional ER import signals determine whether ectopic *VSG* transcripts trigger mRNA balancing

We then also replaced the native ILDat2.1 GPI anchor signal with that of *T. brucei* VSG MITat1.11. This, however, led neither to regulation of the endogenous *VSG* mRNA nor to high-level ectopic VSG protein expression, despite the production of high levels of *ILDat2.1* mRNA.

As VSG proteins are synthesised in the secretory pathway, we next considered the possibility that differences in ER targeting might determine whether ectopic transcripts trigger balancing.

**Fig 3:**
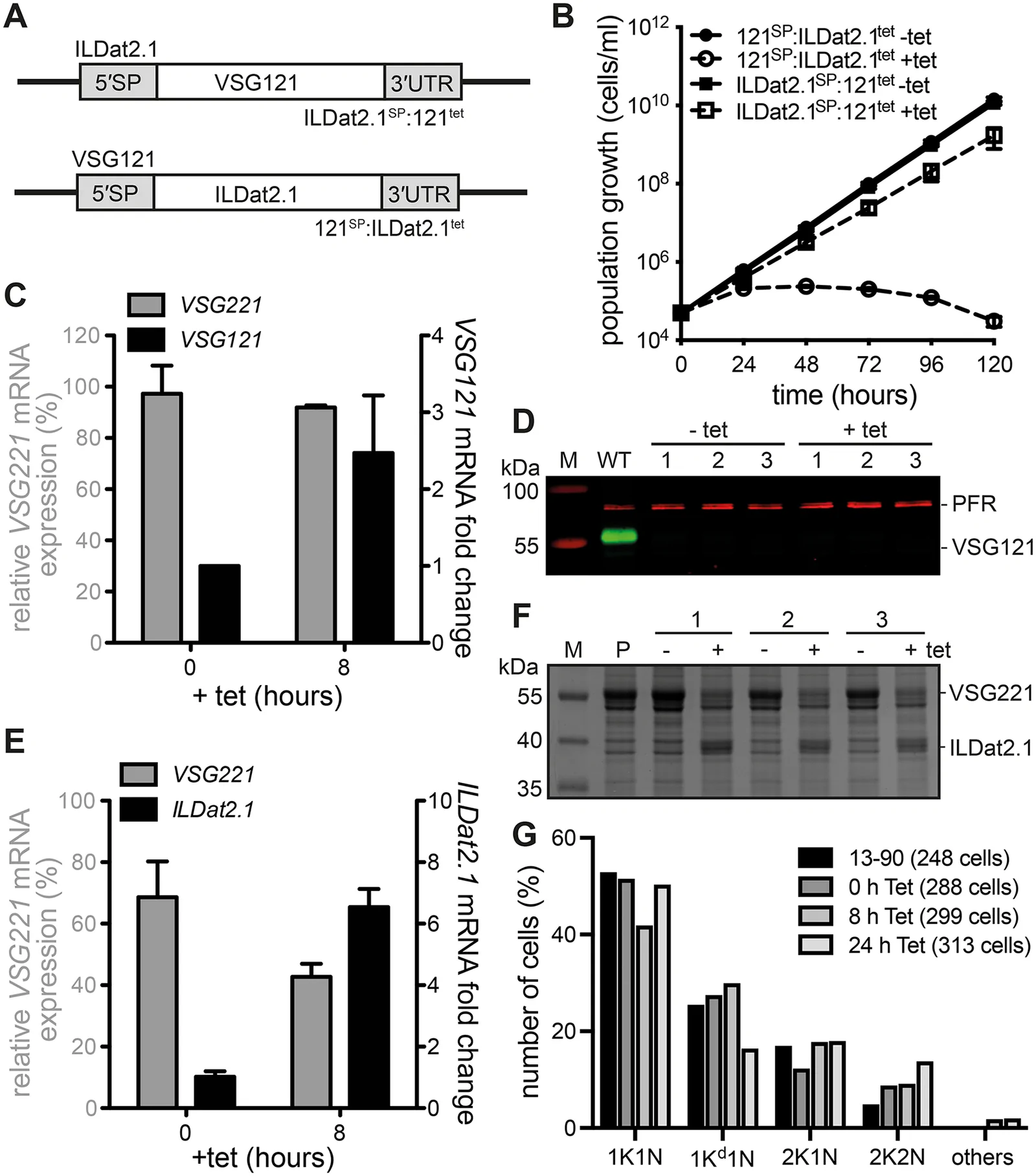
The endogenous *VSG* mRNA is regulated only when the ectopic VSG has a functional ER-import signal. **(A)** Schematic of the constructs used to generate the ILDat2.1^SP^:121^tet^ (upper panel) and 121^SP^:ILDat2.1^tet^ (lower panel) cell lines. **(B)** Cumulative growth curves of the 121^SP^:ILDat2.1^tet^ and ILDat2.1^SP^:121^tet^ cell lines. Three independent clones were analysed for 5 days in the presence (+tet) or absence (-tet) of tetracycline and the values are presented as means ± SD. **(C)** Relative quantification of *VSG* mRNA levels before (0 h) and after (8 h) inducing expression of the ectopic *VSG* in the ILDat2.1^SP^:121^tet^ cells. *VSG221* expression (left y-axis) is shown as a percentage relative to the *VSG221* expression in the parental 13-90 cells, while the *VSG121* expression (right y-axis) is given relative to the non-induced levels. The mRNA was normalised to *tubulin*. Two independent clones were analysed, and the values are presented as means ± SD. **(D)** Western blot analysis of VSG121 protein expression in ILDat2.1^SP^:121^tet^ cells before (-tet) and 24 h after (+tet) induction of expression, detected using anti-VSG121 antibodies. Wild-type (WT) *T. brucei* cells expressing VSG121 served as the positive control (green) and PFR protein (red) served as the loading control. **(E)** Analysis of *VSG* mRNA expression in three clones of the 121^SP^:ILDat2.1^tet^ cells. The endogenous *VSG221* and ectopic *ILDat2.1* are quantified and presented as in **(C)**. **(F)** SDS-PAGE analysis of ILDat2.1 protein expression in the 121^SP^:ILDat2.1^tet^ cells before (-tet) and 24 h after (+tet) addition of tetracycline. Three independent clones of the 121^SP^:ILDat2.1^tet^ cells were analysed, with the 13-90 parental cells (P) serving as a reference. Both the endogenous VSG221 and overexpressed ILDat2.1 bands are labelled. Assignment of the ILDat2.1 band is putative and based on its appearance only following induction of expression and its expected size range between 35 and 40 kDa. **(G)** Cell cycle analysis of 121^SP^:ILDat2.1^tet^ (clone 1) at 0 h, 8 h and 24 h of induction with tetracycline. Parental 13-90 cells served as a reference. Approximately 300 cells were analysed per sample (see figure legend for the number of cells analysed for each sample). Cells were classified according to the number of kinetoplasts (K) and nuclei (N) present as 1K1N, 1K^d^1N (dividing kinetoplast), 2K1N or 2K2N. Cells with aberrant K/N configurations were grouped as “others”.

We performed two experiments to test this possibility: (i) we tried to express *T. brucei* VSG121 with the potentially defective ILDat2.1 ER import signal (Fig 3A, upper panel), and (ii) we tried to rescue expression of ILDat2.1 by replacing the ILDat2.1 ER import signal with that of *T. brucei* VSG121 (Fig 3A, lower panel).

Induction of the ILDat2.1^SP^:121^tet^ reporter produced *VSG121* mRNA but did not affect parasite growth and no *VSG* mRNA balancing was observed, as endogenous *VSG221* mRNA levels in the ILDat2.1^SP^:121^tet^ cell line remained at pre-induction expression levels (Fig 3B, 3C). Western blotting failed to detect the VSG reporter, indicating that protein expression was absent or below the detection limit of the assay (Fig 3D). Since endogenous *VSG* mRNA and protein were expressed abundantly, the cells grew well.

This result is consistent with defective ER targeting of the transcript, explaining why ILDat2.1 expression does not trigger *VSG* mRNA balancing, which was further supported by a further experiment. Induction of expression of ILDat2.1 VSG fused to the VSG121 ER import signal led to markedly impaired cell growth within 24 h, followed by cell death. Analysis of *VSG* transcripts in the 121^SP^:ILDat2.1^tet^ cell line showed that expression of the VSG chimera caused a reduction of the endogenous *VSG221* mRNA to ~40% of the wild-type levels within 8 h (Fig 3E). Interestingly, despite efficient transcription of the reporter *VSG* mRNA, only a faint protein band between 35 – 40 kDa was observed, indicating that the recombinant *T. vivax* VSG protein was poorly expressed (Fig 3F). This assignment remains tentative due to the lack of a specific antibody; however, the increased intensity of a band in the expected size range for the *T. vivax* VSG across all three induced clones is consistent with this interpretation.

To test whether the low detectability of the putative ILDat2.1 protein reflected proteostasis-dependent turnover, we treated induced 121^SP^:ILDat2.1^tet^ cells with the p97/VCP inhibitor CB-5083 [47]. This treatment did not lead to a clear increase in the intensity of the putative ILDat2.1 band, although this analysis is limited by the absence of a specific antibody (S5 Fig).

The marked decrease in endogenous *VSG* mRNA resulted in depletion of wild-type VSG protein and eventually in cell death (Fig 3F, 3B). Together, these experiments indicate that successful ER targeting of the ectopic transcript is required to trigger a *VSG* mRNA balancing response, but that efficient VSG protein production is not.

We also analysed nuclear and kinetoplast configurations after induction. 121^SP^:ILDat2.1^tet^ caused only a mild and heterogeneous shift in cell-cycle distribution, with a modest increase in 2K2N and abnormal cells by 24 h (Fig 3G). Population volume measurements did not reveal a pronounced uniform shift, consistent with the absence of a strong synchronous cell-cycle arrest (S7 Fig).

### ER proximity of ectopic *VSG* transcripts depends on a functional ER import signal

To further confirm that an ILDat2.1 ER import signal mistargeted the mRNA and that indeed *VSG* mRNA must be targeted to the ER for *VSG* mRNA balancing, we investigated the cellular localisation of *ILDat2.1* mRNA containing the native defective ER import signal and a chimera with a functional VSG121 ER import signal.

For this, we analysed the intracellular localisation of *ILDat2.1* transcripts using single-molecule fluorescence *in situ* hybridisation (smFISH) combined with ER marker staining. These experiments directly test whether the ILDat2.1 signal peptide fails to direct transcripts to the ER.

**Fig 4:**
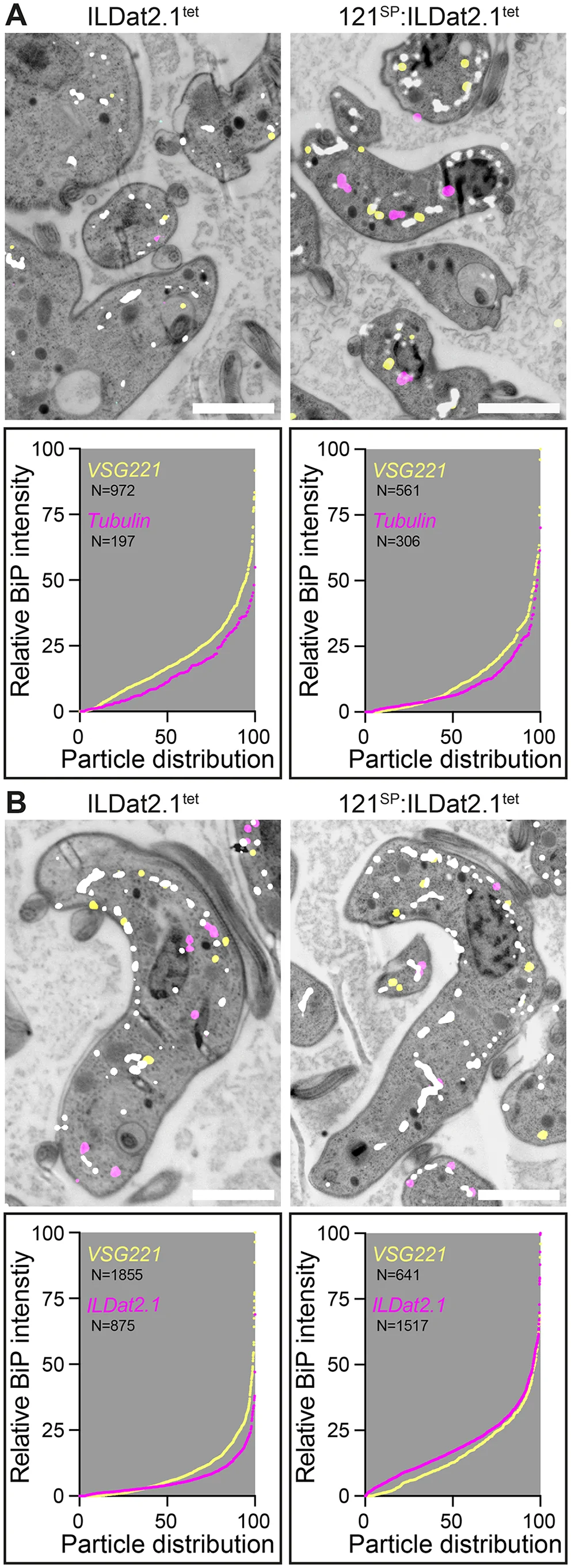
*ILDat2.1* mRNA is only efficiently transported to the ER when it contains a *bona fide* ER-import signal from a *T. brucei* VSG. **(A)** Top: Representative correlative light (smFISH and immunofluorescence) and electron microscopy (inverted SEM) images of LR White-embedded trypanosomes expressing wild-type ILDat2.1 or 121^SP^:ILDat2.1, showing the localisation of *VSG221* mRNA (yellow), *α-tubulin* mRNA (magenta) and BiP protein (White). Scale bars: 2 µm. Bottom: Pairwise comparative quantitative analysis of mRNA proximity to the ER (BiP signal) for *VSG221* (yellow) and *α-tubulin* (magenta) mRNA. The graph shows the intensity of the BiP signal at the respective location of individual mRNA molecules of *VSG221* and *α-tubulin*. The number (N) of individual mRNA molecules analysed are shown. Intensities are plotted sorted from lowest to highest intensity with the intensities given relative to the most intense BiP signal and the particles plotted normalised to the total amount of particles analysed in each data set (particle distribution) to allow comparison of the two curves. **(B)** Top: Representative correlative light (smFISH and immunofluorescence) and electron microscopy (inverted SEM) images of LR White-embedded trypanosomes expressing wild-type ILDat2.1 or 121^SP^:ILDat2.1, showing the localisation of *VSG221* mRNA (yellow), *ILDat2.1* mRNA (magenta) and BiP protein (White). Scale bars: 2 µm. Bottom: Pairwise comparative quantitative analysis of mRNA proximity to the ER (BiP signal) for *VSG221* (yellow) and *ILDat2.1* (magenta) mRNA. The graph shows the intensity of the BiP signal at the respective location of individual mRNA molecules of *VSG221* and *ILDat2.1*. The number (N) of individual mRNA molecules analysed are shown. Intensities are plotted sorted from lowest to highest intensity with the intensities given relative to the most intense BiP signal and the particles plotted normalised to the total amount of particles analysed in each data set (particle distribution) to allow comparison of the two curves. Individual images of the composite images shown here are provided in S2 Fig. Additional analyses of mRNA proximity to the ER are shown in S3 Fig.

The method preserves the integrity of cells and allows the detection of even highly abundant RNA as single molecules [48]. We used two trypanosome cell lines, ILDat2.1^tet^ and 121^SP^:ILDat2.1^tet^, which inducibly expressed ILDat2.1 VSG with either a defective or a functional ER import signal against a VSG221 background. The LR White embedded trypanosomes (8 h post induction) were probed with Affymetrix probe sets for *VSG221* (yellow) and *ILDat2.1* (magenta), with *α-tubulin* mRNA (magenta) serving as a marker for cytoplasmic mRNA. The samples were then incubated with an antibody against BiP, a protein that localises to the lumen of the endoplasmic reticulum (ER) (white). Subsequently, cells were prepared for scanning electron microscopy (as described in Methodology). Quantitative proximity analysis, based on local BiP fluorescence intensity at the site of individual mRNA molecules, showed that the endogenous *VSG221* mRNA was located closer to the ER marker BiP than the cytoplasmic *α-tubulin* mRNA in both cell lines (Fig 4A). Like *α-tubulin* mRNA, *ILDat2.1* transcripts carrying the native ILDat2.1 signal showed lower BiP-proximity values than *VSG221* mRNA, whereas replacement with the functional VSG121 ER import signal increased transcript proximity to BiP to slightly above that of *VSG221* transcripts (Fig 4B). These results indicate that the native ILDat2.1 signal peptide is defective in mediating ER engagement, while the VSG121 signal restores ER engagement of the transcript. This is consistent with the observation that only transcripts carrying a functional ER import signal trigger *VSG* mRNA balancing. Individual images corresponding to the overlays shown in Fig 4 are presented in S2 Fig, with additional analyses provided in S3 Fig.

### Non-VSG ER import signals target *VSGs* to the ER but differentially influence the regulation of total *VSG* mRNA levels

Having established that ER targeting of transcripts correlates with *VSG* mRNA balancing, we next asked whether this response requires VSG-specific ER import signals.

We selected the ER import signals of abundant GPI-anchored surface proteins of different insect stages of *T. brucei* (including the epimastigote-specific *brucei* alanine-rich protein (BARP) and the procyclic EP1 protein) as well as the non-abundant procyclic factor H receptor (FHR) [49–51]. These surface proteins, like the VSGs, traverse the secretory pathway. Thus, we generated *VSG121* transgenes with BARP (UniProtKB: C9ZZN9), EP1 (UniProtKB: Q389V1) and FHR (UniProtKB: Q57Z47) ER import signals. This time, we decided to use constitutive expression from the VSG221 expression site. We have shown that integrating *VSG121* downstream of the *VSG221* results in a 70:30 expression ratio of *VSG221* and *VSG121* mRNA, respectively, relative to the respective wild-type amounts (S4 Fig).

**Fig 5:**
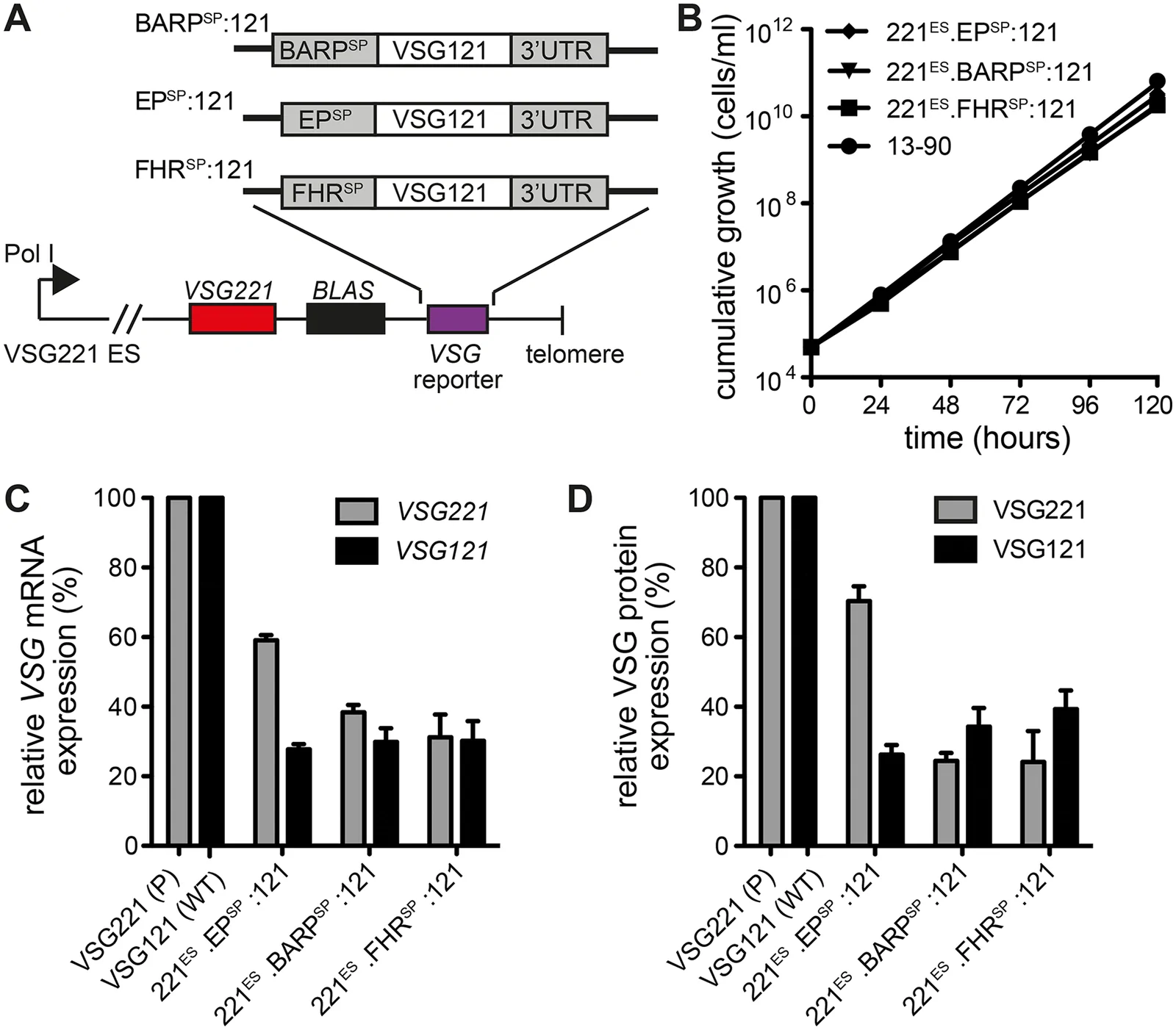
Non-VSG ER import signals target *VSGs* to the ER, but regulation of the endogenous *VSG* mRNA differs. **(A)** Illustration of the constructs and expression strategy used to generate the 221^ES^.BARP^SP^:121, 221^ES^.EP^SP^:121 and 221^ES^.FHR^SP^:121 cell lines. The employed BARP, EP and FHR ER import signal sequences are provided in S3 Table. **(B)** Analysis of the growth curves showed only a slight reduction of growth in all of the generated cell lines as compared to the parental 13-90 cells. The data are presented as means ± SD of three independent clonal cell lines. **(C)** Relative quantification of *VSG* mRNA and **(D)** protein levels. *VSG221* mRNA expression levels are relative to the parental (P) 13-90 *VSG221* expression levels while *VSG121* expression is relative to the wild-type (WT) *VSG121* expression levels. The mRNA was normalised to *tubulin* mRNA whereas the VSG protein was normalised to the PFR protein. The values are presented as means ± SD of three independent clones for all cell lines except 221^ES^.FHR^SP^:121, where two independent clones were used for the quantification of *VSG* mRNA.

We integrated the recombinant *VSG* genes just downstream of the active ES resident *VSG221* to generate the cell lines 221^ES^.BARP^SP^:121, 221^ES^.EP1^SP^:121 and 221^ES^.FHR^SP^:121 (Fig 5A).

The three experiments yielded clonal trypanosome cell lines which showed only minor growth effects (Fig 5B). At the transcript level, a *trans*-regulation of the *VSG* mRNA was elicited in all three clones. In the 221^ES^.BARP^SP^:121 cell line, the endogenous *VSG221* and the ectopic *VSG121* were expressed at approximately 40% and 30% of the wild-type levels, respectively, whereas both *VSGs* were approximately 30% of the wild-type levels in the 221^ES^.FHR^SP^:121 cell line. In the 221^ES^.EP1^SP^:121 cell line, the endogenous *VSG221* and the ectopic *VSG121* were expressed at approximately 60% and 30% of the wild-type levels, respectively (Fig 5C). Although the *trans*-regulation mechanism was operational in the 221^ES^.BARP^SP^:121 and 221^ES^.FHR^SP^:121 cells, the total *VSG* mRNA was ~ 70% of the wild-type levels and the ectopic VSG121 protein appeared to be expressed more than the endogenous VSG221 (Fig 5D). Together, these results show that ER import signals from non-VSG surface proteins are sufficient to support *VSG* mRNA balancing.

### ER-targeted *GFP* is sufficient to trigger *VSG* mRNA balancing

If ER-targeted transcripts are indeed the trigger for *VSG* mRNA balancing, then expression of a non-*VSG* transcript that is efficiently targeted to the ER should be sufficient to elicit the response. To test this prediction, we expressed GFP reporters either in the cytosol or targeted to the ER. The first reporter consisted of the *GFP* ORF and the *VSG121* 3′ UTR. This construct was integrated into an rDNA spacer to generate the 221^ES^.GFP^tet^ cell line that inducibly expresses GFP in the cytosol. We also generated a second reporter for expression of ER-targeted GFP. In addition to the *VSG* 3′ UTR sequence, a procyclin (EP1) ER import signal was coupled to the 5′ end of the *GFP* ORF and integrated into a transcriptionally silent rDNA spacer, yielding the 221^ES^.EP^SP^:GFP^tet^ cell line that inducibly expresses an ER-targeted GFP reporter.

**Fig 6:**
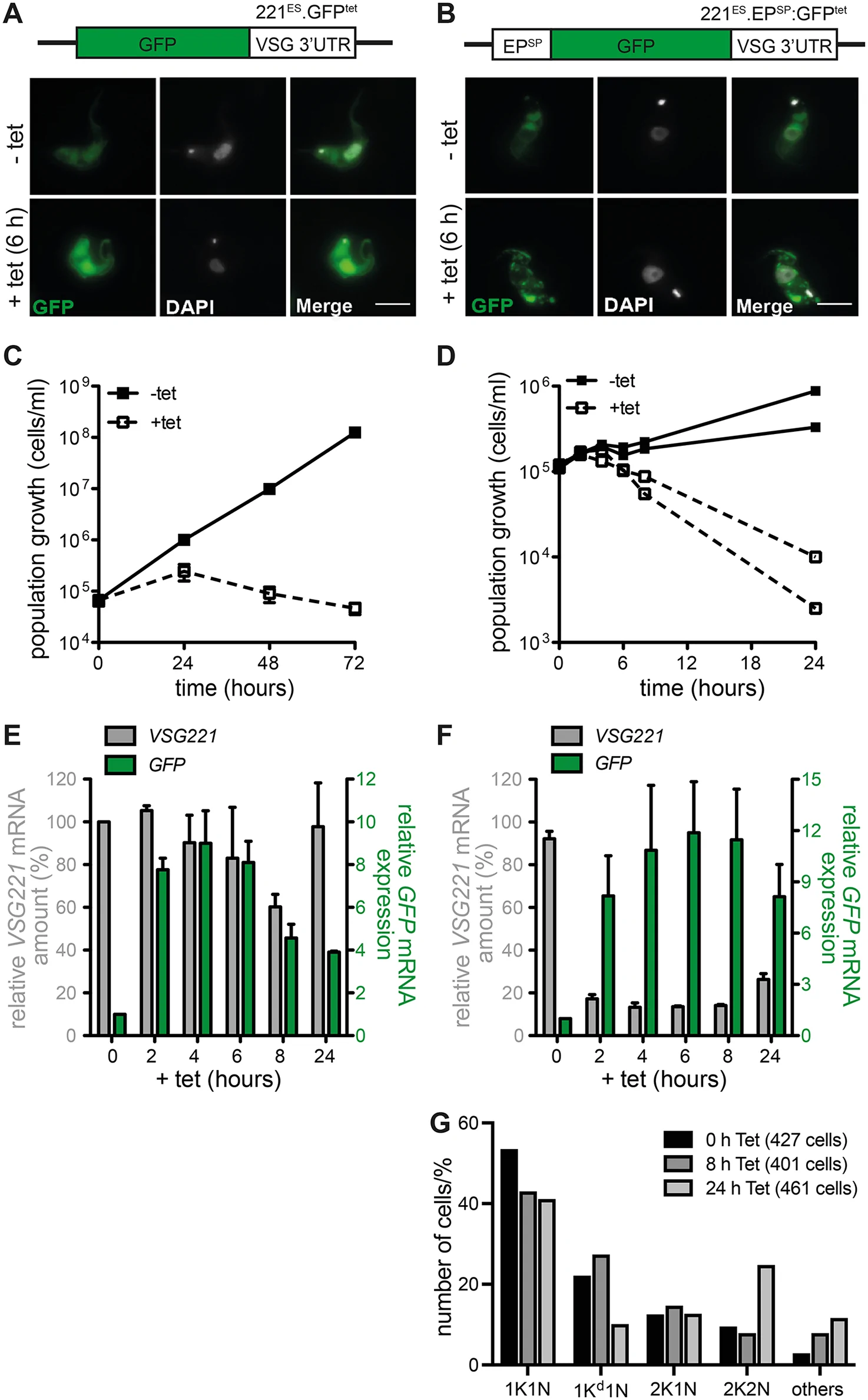
High-level expression of ER-targeted *GFP* reporter causes downregulation of *VSG* mRNA amounts. **(A, B)** Schematic of the GFP reporter constructs and fluorescence microscopy images of fixed cells showing the expression of GFP before (-tet) and 6 h post-induction of expression with 1 µg/ml tetracycline (+tet) in the 221^ES^.GFP^tet^ cells expressing cytosolic and nuclear GFP **(A)**, and 221^ES^.EP^SP^:GFP^tet^ cell line expressing GFP in the ER **(B)**. GFP fluorescence is shown in green while parasite nuclei and kinetoplasts were stained with 4,6-diamidino-2-phenylindole (DAPI, shown in white). Scale bar: 5 µm. **(C)** Growth curves showing cumulative cell numbers of three clonal cell lines grown for 72 h in the absence (-tet) or presence (+tet) of cytosolic *GFP* reporter induction. **(D)** Growth curves showing cumulative cell numbers of two independent clones grown for 24 h in the absence (-tet) or presence (+tet) of ER targeted *GFP* reporter induction. The data in **(C)** and **(D)** are averages with error bars representing the SD. **(E, F)** Relative quantification of *VSG* (left y-axis) and *GFP* (right y-axis) mRNA levels during the course of tetracycline induced cytosolic **(E)** and ER targeted **(F)** *GFP* reporter overexpression. Total RNA samples were dot-blotted and hybridised with fluorescently labelled probes specific for *VSG221*, *GFP* and *tubulin*. The data were quantified by normalisation to *tubulin*. *VSG* mRNA expression is presented as percentage means ± SD for two independent clones normalised to the parental 13-90 cells expressing *VSG221* whereas *GFP* expression values are relative to the non-induced cells, -Tet = 1. **(G)** Cell cycle analysis of 221^ES^.EP^SP^:GFP^tet^ (clone 2) at 0 h, 8 h and 24 h of induction with tetracycline. Approximately 400 cells were analysed per sample (see figure legend for the number of cells analysed for each sample). Cells were classified according to the number of kinetoplasts (K) and nuclei (N) present as 1K1N, 1K^d^1N (dividing kinetoplast), 2K1N or 2K2N. Cells with aberrant K/N configurations were grouped as “others”.

After inducing expression, fluorescence microscopy revealed a strong GFP signal in the cytoplasm and nucleus of the cell line expressing the GFP reporter lacking the ER import signal (Fig 6A). On the other hand, a patchy expression of GFP was observed in a compartment consistent with the ER after inducing the expression of ER targeted GFP (Fig 6B). A slight reduction in growth was recorded within the first 24 h of cytosolic reporter overexpression, after which the cell numbers began to decline (Fig 6C), likely reflecting the burden of high-level reporter expression [52]. In the cell line expressing the ER-targeted reporter, a rapid stalling in parasite growth was observed after inducing expression, followed by a decline in cell numbers within 6 h (Fig 6D). As we were specifically interested in very early effects after induction, this later growth defect did not affect the interpretation of the data. On the transcript level, quantitative dot blots showed an immediate increase in the *GFP* mRNA in the 221^ES^.GFP^tet^ cell line, with peak *GFP* expression recorded between 2 – 6 h after induction, followed by a decline. The two VSG 16-mer containing *GFP* transcripts – one cytosolic, the other ER-targeted – differed in their capabilities to balance the endogenous *VSG* mRNA. There was no impact on the *VSG* mRNA levels within the first 6 h of inducing GFP lacking an ER-targeting signal. However, after 8 h, there was a reduction of both the *VSG* and *GFP* mRNAs. The *VSG* transcript reverted to wild-type levels after 24 h (Fig 6E).

In contrast, expression of the ER-targeted GFP reporter caused a rapid reduction of endogenous *VSG* mRNA. The endogenous *VSG221* mRNA decreased to 20% of the wild-type level within 2 h of expression (Fig 6F). Because ER-targeted GFP caused a particularly strong growth phenotype, we next asked whether proteostasis contributed to GFP detectability and toxicity. CB-5083 treatment increased ER-targeted GFP levels by approximately 1.6–1.9-fold, consistent with partial proteostasis-dependent turnover of the reporter (S6 Fig). Thus, protein quality-control processes contribute to the downstream phenotype of ER-targeted GFP, but this does not alter the conclusion that early *VSG* mRNA attenuation is triggered by ER engagement rather than by a *VSG* open reading frame in the experimental setup used here. Cell-cycle analysis revealed a reproducible increase in 2K2N cells after induction of ER-targeted GFP expression (Fig 6G). This phenotype was more pronounced than for 121^SP^:ILDat2.1^tet^, indicating that different ER-engaged reporters can elicit distinct downstream cellular consequences despite triggering *VSG* mRNA balancing. Cell volume measurements and representative microscopy images are shown in S7 Fig and S8 Fig, respectively.

The results of these experiments suggested that, in fact, the regulation of endogenous *VSG* did not require the ectopic *VSG* open reading frame or a VSG-specific ER import signal.

**Fig 7:**
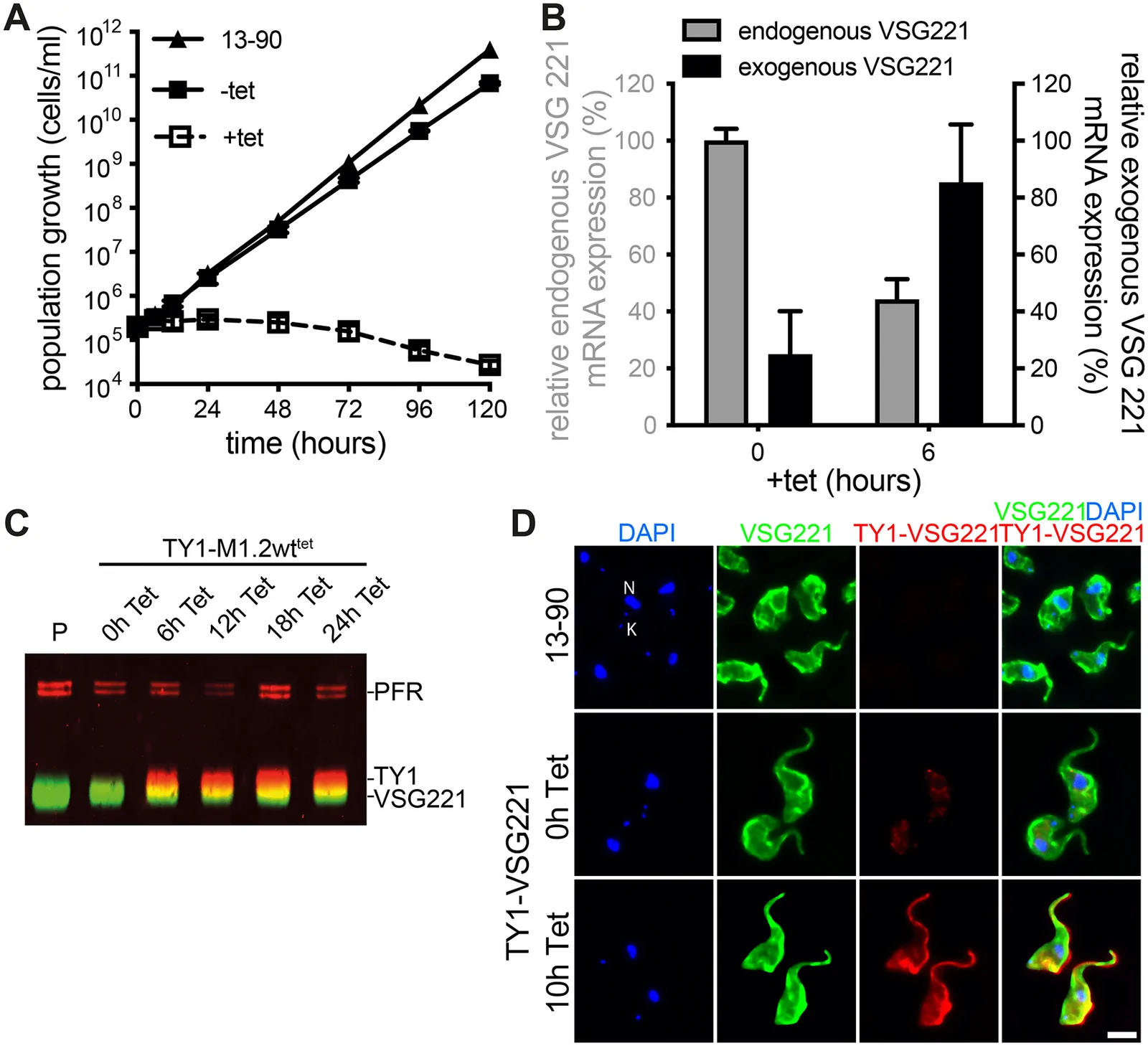
Inducible expression of TY1-tagged VSG221 VSG triggers rapid mRNA balancing and acute growth arrest despite correct protein expression and localisation. **(A)** Growth curves of three independent clones with or without tetracycline induction of TY1-VSG221 expression. Induction leads to rapid growth arrest within 6 h and a subsequent decline in cell numbers. **(B)** Quantification of endogenous and exogenous *VSG221* mRNA levels before and 6 h after induction of TY1-VSG221 expression. Bars represent the relative contributions of endogenous *VSG221* and exogenous *TY1-VSG221* transcripts to total *VSG221* mRNA. Exogenous transcript levels were calculated by subtracting endogenous M1.2 signal (detected using a 5′ UTR-specific probe), from total *VSG221* mRNA (detected using a probe targeting the ORF). The values are presented as means ± SD of three independent clones. **(C)** Protein analysis by Western blot showing expression of TY1-VSG221 (TY1, red) following induction with tetracycline for 0, 6, 12, 18 and 24 h alongside total VSG221 levels (green). PFR (red) acts as a loading control. **(D)** Immunofluorescence analysis of cells before (0 h) and 24 h post induction of TY1-VSG221 expression demonstrates efficient trafficking of TY1-VSG221 to the cell surface. The parental 13–90 cells are shown as a control. Both endogenous and ectopic TY1-tagged VSG221 were detected with an anti-VSG221 antibody (green) with the ectopic TY1-VSG221 specifically detected with an anti-TY1 antibody (red). Kinetoplasts and nuclei were stained with DAPI (blue). Scale bar: 5 µm.

To further test whether *VSG* mRNA balancing depends on successful production of a functional surface coat, we analysed an ectopically expressed TY1-tagged VSG221 VSG. Induction of TY1-VSG221 caused rapid growth arrest, followed by a decline in cell numbers (Fig 7A). Within 6 h of induction, *TY1-VSG221* transcripts accounted for the majority of total *VSG221* mRNA, accompanied by a marked reduction of endogenous *VSG221* mRNA (Fig 7B). This reciprocal shift is consistent with rapid *VSG* mRNA balancing.

Importantly, TY1-VSG221 protein was detectable after induction and immunofluorescence analysis showed localisation of the tagged VSG at the cell surface (Fig 7C, 7D). Thus, the balancing response can be activated even when the resulting VSG fusion protein is deleterious. These data support the interpretation that *VSG* mRNA balancing is initiated upstream of final surface coat functionality, most plausibly at the level of abundant ER-engaged *VSG* transcripts.

Together, these experiments demonstrate that *VSG* mRNA balancing does not depend on the identity of the *VSG* open reading frame or on a VSG-specific signal peptide. Instead, the response is triggered specifically by the presence of abundant transcripts that engage the ER targeting machinery. Constructs that generate large numbers of ER-engaged transcripts consistently elicit attenuation of endogenous *VSG* mRNA, whereas highly expressed transcripts that remain cytosolic fail to do so. These observations indicate that the parameter monitored by the cell is the abundance of ER-engaged transcripts rather than VSG-specific sequence features or productive VSG protein synthesis.

We therefore conclude that, in the experimental systems analysed here, ER engagement is required for rapid attenuation of endogenous *VSG* mRNA.

This finding suggests that trypanosomes couple the cytoplasmic load of ER-targeted transcripts to regulation of the nuclear VSG expression site.

## Discussion

In this study, we investigated the mechanism underlying the balancing of *VSG* mRNA levels in bloodstream-form *Trypanosoma brucei*. Previous work showed that when a second VSG is expressed, the total amount of *VSG* mRNA rapidly returns to approximately wild-type levels, suggesting the existence of a homeostatic regulatory mechanism. Here, we demonstrate that the conserved 16-mer, present in all expressed VSGs, alone is not sufficient to initiate balancing and instead this balancing response is triggered specifically by the presence of abundant transcripts that are targeted to the endoplasmic reticulum (ER). The response does not require the *VSG* open reading frame or efficient VSG protein production, as high-level expression of ER-targeted *GFP* is sufficient to elicit attenuation of endogenous *VSG* mRNA, whereas highly abundant transcripts lacking ER-targeting signals fail to do so. These findings indicate that the signal for *VSG* mRNA regulation is the presence of ER-engaged transcripts rather than VSG-specific sequence features. Together with previously published data, these findings support an ER-engagement-coupled homeostatic attenuation model in which an increased load of ER-targeted transcripts triggers DOT1B-dependent attenuation of the active VSG expression site, thereby rapidly restoring a steady-state set point for *VSG* mRNA abundance.

Antigenic variation is a powerful strategy employed by several pathogens to evade elimination by host immune responses. Monoallelic expression of surface antigens by parasitic pathogens, such as trypanosomes and *Plasmodium falciparum*, ensure that only a single antigen is expressed at a time from a vast repertoire of silent genes [53,54]. In trypanosomes, variation of the VSG antigens involves transcriptional switching or recombination events that introduce a new antigen into the active VSG expression site [55].

In a previous study, an *in situ* VSG switch was simulated by inducible and high-level expression of a second VSG from the ribosomal spacer [35]. When a *VSG121* gene was expressed ectopically, an almost instantaneous reaction from the expression site was consistently found: the mRNA of the ES-resident *VSG* declined rapidly to low levels. It was further shown when overexpressing *VSG121* in a *VSG221* background that the parasites, in a next step, can attenuate the expression site in a DOT1B-dependent manner [35]. This, however, is a stochastic process. Using a pleomorphic trypanosome strain, it was shown that in a clonal population, ES-silencing occurred in some 50% of all cells, while all cells silenced the VSG. This means that ES-silencing is independent from VSG silencing [36]. This is also supported by the fact that the overexpression of VSG221 in VSG221 expressing monomorphic cells resulted in a VSG switch but had no significant effect on growth suggesting that only the VSG but not the ES was silenced [46]. Furthermore, not all ectopically expressed *VSG* genes were affecting the ES-derived *VSG* mRNA with equal efficiency, and not all ESs were equally responsive. Thus, trypanosomes exhibit a remarkable degree of phenotypic plasticity and high cell-to-cell variability. This makes perfect sense for a parasite that, during its life cycle, encounters extremely different microenvironments and that does not regulate gene expression by transcriptional control, as most other eukaryotes do. Instead, posttranscriptional control is paramount [23]. Previous studies established that ectopic VSG expression can trigger *VSG* mRNA balancing but did not identify which feature of the ectopic construct initiates the response. The experiments presented here progressively exclude several possibilities: the response does not require a particular *VSG* open reading frame, VSG protein identity, or a VSG-specific ER import signal. Instead, across the different constructs analysed, the common feature associated with rapid balancing is competence for ER engagement. We therefore consider the principal advance of this study to be the identification of ER engagement as an upstream requirement for the balancing response, while the molecular sensor and downstream mechanism remain to be determined.

Our results support an ER-engagement-coupled homeostatic attenuation mechanism for *VSG* mRNA balancing. In this framework, induction of an ectopic secretory-pathway transcript rapidly increases the pool of ER-engaged mRNAs (i.e., transcripts undergoing co-translational targeting/engagement with the ER import machinery). When this pool exceeds a functional set point for ER engagement capacity, the cell rapidly attenuates endogenous *VSG* mRNA levels via the expression-site attenuation mechanism described previously (e.g., DOT1B-dependent attenuation), thereby restoring ER-engaged transcript load toward baseline, below the functional set point. Our experiments do not exclude transcript turnover as an additional component of this response. Transcripts that exceed available ER-engagement capacity could potentially remain non-engaged and become selectively destabilised. Distinguishing altered transcription from differential transcript turnover will require direct kinetic measurements of both processes. Protein quality-control mechanisms may additionally influence the detectability and stability of some reporter proteins, particularly ILDat2.1 and ER-targeted GFP. However, these downstream processes are not required to explain the early *VSG* mRNA balancing response observed in the present study.

Our data further support the conclusion that the determining factor is the abundance of ER-engaged transcripts rather than the identity or translation efficiency of the encoded protein. Based on our previous work, expression-site attenuation represents one plausible downstream mechanism for reducing the dominant Pol I-derived VSG transcript pool when ER-engaged transcript load increases; however, this connection was not directly tested in the present study.

In all previous experiments, one observation was consistently made: the inducible expression of a second VSG only led to a very short period of overshooting total *VSG* mRNA, which then rapidly levelled to approximately wild-type amounts [35,36]. The simultaneous increase of ectopic *VSG* mRNA and decline of ES-resident *VSG* mRNA could be observed. This was not only documented by overexpressing VSGs, but also by generating so-called double expressor cell lines, in which two *VSG* genes are constitutively expressed in tandem from the same expression site. VSG double expressors were first generated in 1996 [32], but the levelling of *VSG* mRNAs long remained undetected or anecdotal. We propose that trypanosomes maintain secretory pathway homeostasis by preventing excessive accumulation of highly abundant ER-engaged transcripts [35,36]. We have shown that less than 50% of wild-type *VSG* mRNA levels is sufficient for VSG coat formation and hence the parasites seem to operate on the safe side [56]. In the present study, we have explored some basic features of the *VSG* mRNA that are involved in mRNA balancing. We first asked if the process requires a *T. brucei* VSG and hence if it is species-specific. The related African trypanosomes *T. congolense* and *T. vivax* also express VSGs on their cell surface and the VSG coat is subject to antigenic variation, however, the VSG proteins may be divergent from *T. brucei*. While we have studied VSGs from both species, for the present study, we decided to challenge *T. brucei* with overexpression of two *T. vivax* VSGs. This turned out to be more complex than we thought.

Inducing the expression of the *T. vivax* ILDat1.2 VSG resulted in a rapid reduction of the endogenous *T. brucei VSG221* mRNA amounts, similar to the results obtained when *T. brucei* VSGs were overexpressed [35]. However, inducing the expression of another *T. vivax* VSG, ILDat2.1, did not affect the endogenous *VSG221* mRNA levels, despite the efficient production of *ILDat2.1* transcript. We initially thought that this result might reflect the high phenotypic flexibility of trypanosomes. However, we had never observed high-level expression of *VSG* mRNA not resulting in mRNA balancing. Therefore, we surmised that the *ILDat2.1* transcript lacked an essential feature.

The first candidate was the ER import signal sequence, as *ILDat2.1* transcripts failed to produce readily detectable protein. In the present work, we use “ER engagement” operationally to denote functional coupling of the transcript to ER targeting/import, irrespective of the precise contribution of SRP-dependent versus SRP-independent routes [57]. This operational definition is consistent with current models in which ER-associated translation reflects the coordinated interaction of mRNAs, ribosomes and the ER translocation machinery, and is not solely explained by signal peptide-mediated targeting [12–14].

It has been suggested, however, that procyclic trypanosomes and *S. cerevisiae* appear to rely on the SRP-independent pathway for the translocation of GPI-anchored secretory proteins, and the SRP-dependent pathway for transmembrane-bearing proteins [58,59]. In *S. cerevisiae*, cytosolic factors including the heat shock protein 40 (Hsp40), yeast dnaJ protein 1 (Ydj1), and Hsp70 are all involved in the SRP-independent translocation of GPI-anchored proteins [58].

Indeed, the expression of a transcript of a hybrid *ILDat2.1 VSG* reporter containing a *bona fide T. brucei* VSG ER import signal resulted in the expected balancing of endogenous *VSG221* mRNA, proving that the process can be triggered by expression of a non-*T. brucei* VSG. The second insight from this experiment was that balancing is most probably independent from efficient VSG production, because, although the ER signal was functional, ILDat2.1 protein appeared to be poorly made. Lastly, the experiment strongly suggested that for mRNA balancing to operate, the transcripts need to be targeted to the ER.

The next questions we asked were (i) if the ER-signal must be VSG-specific and (ii) if mRNA balancing requires a VSG at all. The ER import signal peptides of GPI-anchored *T. brucei* proteins VSG, BARP, EP1 and FHR all conform to the general organisation of signal peptides [60,61]. In fact, we found that the ER import signals of BARP, EP1 and FHR can efficiently target VSGs to the ER. Interestingly, *VSG* mRNA was differentially regulated in VSG double expressor cell lines expressing ectopic VSG121 with BARP or FHR ER import signals, compared to cells expressing ectopic *VSG121* with its native or EP1 ER import signal. The mRNAs of the endogenous *VSG221* and ectopic *VSG121* with its native (S4 Fig) or EP1 ER import signal were expressed at a 70:30 ratio. In contrast, equal transcript levels of endogenous *VSG221* and ectopic *VSG121* were observed in cell lines expressing ectopic VSG121 with BARP and FHR ER import signals. These results agree with previous findings which showed that ER import signals can differentially influence the translocation and processing of substrates [6,62,63]. It has been reported that specific motifs in the h-region can determine the efficiency of translocation of substrates into the ER of *T. brucei* [64]. Therefore, possibly the variations in *VSG* mRNA balancing were due to differences in the h-region motifs of the reporter VSG ER import signals. Importantly, however, the experiments clearly showed that the VSG ER import signal does not harbour special features required for the balancing of *VSG* mRNA.

To address the question whether the “signal” for mRNA balancing resides in the *VSG* open reading frame, we used ER-targeted GFP and GFP lacking an ER-targeting signal coupled to the *VSG* 3′ UTR to ensure high levels of expression. In agreement with all data obtained so far, we showed that high expression levels of ER-targeted *GFP* from the rDNA spacer locus resulted in a *trans*-regulation of the *VSG* mRNA. This regulation was absent when the *GFP* reporter was not targeted to the ER. The GFP reporter expression experiments showed that *VSG* mRNA balancing is neither dependent on the *VSG* open reading frame nor on the VSG ER import signal.

These results demonstrate that, in our experimental framework, high-level expression of an ER-targeted transcript can be sufficient to trigger attenuation of endogenous *VSG* mRNA.

The only common denominator in all our experiments was the presence of an enigmatic 16‑mer motif in the 3′ UTR of the transcripts. This motif is essential for *VSG* mRNA stability in bloodstream stage parasites and is 100% conserved in all *T. brucei VSG* mRNAs. Interestingly, it is completely absent from the genomes of the related African trypanosome species *T. congolense* and *T. vivax*, which also have a VSG surface coat. The F-box RNA binding protein (CFB2) has been suggested as a limiting factor which interacts with the 16‑mer motif in the *VSG* 3′ UTR, thereby actively regulating *VSG* mRNA abundance [39]. As an alternative mechanism, *VSG* mRNA levels might be modulated by a feedback that is dependent on the production of functional GPI-anchored VSG protein [40].

In our experiments, the ectopic *VSG121* and *ILDat2.1 VSG* reporters with an apparently dysfunctional ILDat2.1 VSG ER import signal, were both fused to a complete *T. brucei VSG* 3′ UTR with an intact 16-mer motif. High levels of 16-mer-containing mRNA accumulated without ER-proximal localisation. However, levels of endogenous *VSG* mRNA were not downregulated, suggesting that the total *VSG* mRNA greatly exceeded the wild-type amounts in these cells. This agrees with the study by Maudlin et al., which shows that *VSG* mRNA can be expressed above the wild-type levels [40]. These observations also imply that, in principle, the available CFB2 protein pool is sufficient to interact with the 16-mer of a second highly expressed *VSG* and can thus stabilise the mRNA. These observations argue against a simple counting mechanism based solely on 16-mer availability.

We, therefore, propose that though the interaction between the 16-mer and CFB2 is essential for high expression and stability of *VSG* mRNA [34,39], the steady-state *VSG* mRNA amount is regulated at a different level.

Maudlin et al. reported that introducing a premature termination codon (PTC) just before the ectopic VSG221 GPI signal altered the behaviour of *VSG* mRNA regulation [40]. One interpretation is that downstream events linked to GPI anchoring influence the response.

Our results instead support a model in which ER targeting/engagement is the initiating requirement for *VSG* mRNA attenuation, while downstream processing steps (including GPI anchoring and proteostasis) may modulate phenotypic outcomes depending on construct and expression-site context.

Previously published studies reported a 50:50 expression of *VSG* mRNA when the wild-type *VSG* was integrated upstream of the endogenous *VSG* into the VSG221 ES [32,33], whereas Maudlin et al., express the second *VSG* upstream of the endogenous *VSG* from the VSG121 ES and the VSG221:VSG121 levels had a 1:3 ratio [40]. Additionally, expressing ectopic VSG221 with PTCs at different locations activated a pathway that increased the total *VSG* mRNA amounts. These variations in the modulation of *VSG* mRNA levels when different ES are used suggests there might be additional intricacies for the regulation of *VSG* mRNA.

In summary, we have shown that *VSG* mRNA balancing is independent of the *VSG*’s ORF and of effective VSG production, but clearly dependent on high levels of ER-targeted transcripts. In view of the biology of trypanosomes this makes sense. Trypanosomes lack transcriptional control and highly abundant surface proteins are expressed by Pol I [23,65]. During antigenic variation, mRNA balancing would cause an exchange of the *VSG* mRNA population before the completion of an expression site switch. During developmental progression to the procyclic insect stage, high-level expression of ER-targeted *procyclin* mRNA could contribute to balancing the amount of *VSG* mRNA present in the cell. This would likewise be true when procyclin is replaced with BARP in the next step of life cycle progression. Thus, trypanosomes exploit a simple but very effective system of posttranscriptional transcript balancing that allows for high phenotypic plasticity and robustness. In some respects, transcript balancing in trypanosomes is reminiscent of a phenomenon called transcript buffering, which was first described in yeast [66,67]. Transcript buffering involves a complex interplay that regulates transcription rates in the nucleus and mRNA decay in the cytoplasm to ensure steady-state mRNA levels are maintained in the cell [68–70]. Transcript buffering has further been demonstrated in mammalian cells [71,72], indicating a conserved process in eukaryotes. Transcript buffering can either be gene-specific or global and is possibly regulated by distinct mechanisms. How signal for crosstalk between transcription and mRNA decay is perceived, and its directionality is not well understood. In yeast, central players in transcript buffering include transcription initiation and the mRNA decay pathway factors [73]. In trypanosomes, the situation is certainly different from yeast and mammals, as transcription is not regulated. However, the basic need for transcript buffering also applies. We call the phenomenon transcript balancing as just two players are involved; in bloodstream stage trypanosomes these are two populations of *VSG* mRNAs during antigenic variation.

We propose that trypanosomes balance abundant secretory-pathway transcripts to match ER engagement capacity and maintain secretory homeostasis. Identifying the molecular sensor that links ER engagement to VSG expression-site attenuation will be an important goal for future work. This mechanism may represent a specialised form of transcript buffering adapted to the extreme transcriptional architecture of trypanosomes.

The conceptual simplicity of the trypanosome system provides opportunities that are less accessible in opisthokont models. It has been suggested that mRNAs with longer half-lives are particularly suitable for studying transcript buffering [66]. Accordingly, the high-level expression and long half-life of *VSG* mRNA can be exploited to investigate transcript buffering in the tractable *T. brucei* system.

## Methodology

### Cultivation and genetic manipulation of trypanosomes

All cell lines generated in this study are based on monomorphic *T. brucei* 427 MITat1.2 13–90 bloodstream-form parasites expressing the T7-polymerase and tetracycline repressor [74]. The cells were maintained below 1 x 10^6^ cells/ml in HMI-9 medium supplemented with 10% heat-inactivated foetal calf serum (Sigma-Aldrich) at 37 °C and 5% CO_2_. For transfections, 10 µg of linearised DNA was transfected into 3.0 x 10^7^ mid-log phase cells in Amaxa Basic Parasite Nucleofector Solution 1 using the X-001 program of an Amaxa Nucleofector II (Lonza, Switzerland). Hygromycin, G418, blasticidin and phleomycin were used at 5, 2.5, 5, and 1 µg/ml, respectively, for the selection of recombinant cell lines. Overexpression was induced with 1 µg/ml tetracycline. Growth rates were monitored for 24 – 120 h and cell densities determined after specified intervals using a Neubauer counting chamber.

Monomorphic wild-type *T. brucei* Lister 427 cells expressing VSG121 were cultivated in HMI-9 medium supplemented with 10% heat-inactivated foetal bovine serum (Sigma-Aldrich) at 37 °C and 5% CO_2_ without antibiotics.

### Plasmid construction and generation of cell lines

The *ILDat1.2* and *ILDat2.1* coding sequences [41–45], each fused to the 3′ UTR of *MITat1.1 VSG*, were synthesized with EcoRI restriction sites at the 5′ and 3′ ends and cloned into pBSK II (+), resulting in plasmids pBSK.ILDat1.2 and pBSK.ILDat2.1, respectively. As the published ILDat2.1 sequence lacks a start codon [44], the first two nucleotides (CC) were replaced with ATG to generate an in-frame open reading frame encoding a protein of 392 amino acids. The inserts were excised using HindIII and SmaI, followed by ligation into pLew82v4 plasmid that was linearised sequentially with XhoI (followed by refilling the overhangs with the Klenow fragment) and HindIII. The pLew.ILDat1.2 and pLew.ILDat2.1 plasmids were NotI-linearised and transfected into *T. brucei* 13–90 cells to generate the 221^ES^.ILDat1.2^tet^ and 221^ES^.ILDat2.1^tet^ cell lines, respectively.

Chimeric sequences were generated by PCR-driven overlap extension [75]. To replace the native *ILDat2.1* ER import signal with a *VSG121* ER import signal, *ILDat2.1:M1.1* 3′ UTR sequence without the ER import signal and the *VSG121* ER import signal were amplified from plasmid pBSK.ILDat2.1 and pLew.121wt, respectively, using long primers that covered the overlap region of the two sequences. The products of the two PCR reactions were used as templates in a subsequent overlap PCR. Fusion of the two overlapping fragments occurred during the initial amplification cycles, after which the generated full-length product was amplified using primers annealing to the start of *VSG121* ER import signal and the end of *ILDat2.1:M1.1* 3′ UTR sequence. The fused fragment was subcloned into pJet1.2 and pBSK II (+). The insert was excised and ligated into the pLew82v4 vector as described above to generate the 221^ES^.121^SP^:ILDat2.1^tet^ cell line. A similar approach was used to replace the native *VSG121* ER import signal with that of *ILDat2.1 VSG* from plasmid pJET.121 and pBSK.ILDat2.1, respectively. Transfection of the linearised construct into the parental cells generated the 221^ES^.ILDat2.1^SP^:121^tet^ cell line.

To replace the ILDat1.2 GPI signal peptide, the ILDat1.2 GPI signal peptide was first predicted using the SignalP version 4.1 online server [76]. Next, the ILDat1.2 ORF lacking the native GPI signal coding sequence and MITat1.11 GPI signal coding sequence (TCCAGTTTTCTAGTAAGCAAACAATTCGCCCTAATGGTTTCTTCTGCATTTGCG GCCTTACTT TTTTAA) coupled to the *MITat1.1* 3′ UTR were separately amplified using long overlapping primers from the plasmid pBSK.ILDat1.2:M1.1 and pKD4.M1.11:M1.1UTR, respectively. The PCR products were fused in a second PCR reaction as described above. A similar approach was used to replace the predicted ILDat2.1 GPI signal peptide with that of MITat1.11 VSG. The ILDat2.1 ORF lacking the GPI signal peptide was amplified from plasmid pBSK.ILDat2.1:M1.1, while the MITat1.11 GPI signal sequence together with *MITat1.1* 3′ UTR was amplified from plasmid pKD4.M1.11:M1.1UTR. The generated hybrid gene fragments were cloned into the pLew82v4 overexpression vector and transfected into the parental *T. brucei* 13–90 cells.

The GFP coding sequence was amplified from plasmid p3822 (kindly provided by M. Carrington, Cambridge, UK) using primers CFP_U.HindIII and 04_GFP_BamHI_L and ligated into pBSK II (+) plasmid with SmaI and BamHI restriction sites. The *VSG* 3′ UTR was amplified from plasmid pBSK.M1.6-198 with primers M1.63′UTRBglII_U and M1.6_full_L and ligated after the GFP coding sequence using Bglll and BamHI restriction sites. The *GFP:VSG121* 3′ UTR hybrid was excised using HindIII and XbaI and cloned into pLew82v4 (Addgene plasmid #24009) to generate the pLew.GFP-198 construct. This construct was then linearised with NotI and transfected into the 13–90 parental cell line to generate the 221^ES^.GFP^tet^ cell line. For the addition of an ER-import signal to the GFP reporter, the coding sequence for the EP1 ER import signal (EP^SP^) was excised from plasmid pLew.SP:GFP:GPI-198 with BstEII. The EP^SP^ was ligated into BstEII linearised pLew.GFP-198 plasmid resulting in the pLew.EP^SP^:GFP-198 plasmid, which was NotI-linearised and transfected into 13–90 cells to generate the 221^ES^.EP^SP^:GFP^+tet^ cell line.

For constitutive expression of VSG121 downstream of the expression site resident *VSG221*, the *VSG121* ORF was amplified from plasmid pRS.121 by PCR. The PCR product was blunted and ligated into pJET1.2:Blas:GFP:UTR after removal of the *GFP* ORF with PacI and PaeI restriction enzymes. Next, the Blas:VSG121:UTR was transferred from the pJET1.2:Blas:VSG121:UTR into a modified pbRN6 [77] and the upstream integration region extended as described above to generate pbRN6.M1.6wt plasmid. The construct was linearised with SacI and SalI and transfected into the 13–90 cells. To replace the VSG121 ER import signal with that of FHR, the region upstream of the *VSG121* gene and the *VSG121* sequence minus the signal peptide was amplified from plasmid pbRN6.M1.6wt using primers that contained the full signal sequence and fused as described above. Replacement of the VSG121 ER import signal with the BARP and EP1 signal sequences was carried out in a similar manner. The reporter sequences were cloned into pJET1.2, excised using EcoRI and HindIII, and cloned into pbRN6.M1.6wt plasmid that was linearised in the same way. Transfection of SacI and SalI linearised constructs into 13–90 cells created the 221^ES^.FHR^SP^:121 and the 221^ES^.BARP^SP^:121 cell lines. All enzymes were obtained from Thermo Fisher Scientific (USA). The primers are available on request.

### RNA analysis

Total RNA was extracted from 1 x 10^8^ parasites using the RNeasy Mini Kit (Qiagen, Netherlands) as per the manufacturer’s instructions. For quantification of mRNA, 3 µg of total RNA was denatured with glyoxal at 50 °C for 40 min and transferred onto a Hybond-N nylon membrane (GE Healthcare, UK) using a Minifold I dot blotter (Schleicher & Schuell, Germany). The blots were hybridised overnight at 42 °C with oligonucleotide probes labelled with fluorescent IRDye 682 (*VSG121* probe: GGCTGCGGTTACGTAGGTGTCGATGTCGAGATTAAG; *VSG221* ORF probe: CAGCGTAAACAACGCACCCTTCGGTTGGTCGTCTAG; *VSG221* 5′ UTR probe: TTCGTGTCGCGTAGGAATAACTACAA;*GFP* probe: GCCGTTCTTCTGCTTGTCGGCCATGATATAGA; *ILDat1.2* and *2.1* probe: TAGGATATCAAGCTTGTGAATTTTACTTTTTGG, targeting the *GPEET* 5′ UTR sequence present in these ectopically expressed transcripts) or IRDye 782 labelled *tubulin* probe*:* ATCAAAGTACACATTGATGCGCTCCAGCTGCAGGTC). Imaging and quantification of fluorescence was carried out using the Li-Cor Odyssey or Li-Cor Odyssey CLx system (Li-Cor, Netherlands) and Image Studio Lite.

### Protein analyses

Trypanosomes were resuspended and lysed in protein sample buffer to yield equivalents of 2 x 10^5^ cells/µl. For quantification of VSG proteins by SDS-PAGE 5 µl of the protein sample, was resolved on 12.5% SDS-PAGE gels. For Western blots 2.5 µl was used and proteins were subsequently transferred onto a nitrocellulose membrane. For quantification by dot blots, 3 µl of the protein sample was applied directly to the nitrocellulose membrane. The membranes were blocked for 1 h at room temperature or overnight at 4 °C with 5% (w/v) skimmed milk powder in PBS. Next, the membranes were incubated with primary antibodies (rabbit anti-VSG221 (1:5,000, polyclonal), rabbit anti-VSG121 (1:2,000, polyclonal; kind gift from M. Carrington), rabbit anti-GFP (1:2,000, polyclonal; #A11122 Invitrogen), mouse anti-TY1 (1:2,000, monoclonal, BB2) and mouse anti-PFR (L13D6) (1:20, monoclonal; kind gift from P. Bastin)) diluted in PBS containing 1% (w/v) skimmed milk powder and 0.1% (v/v) Tween 20. After wash steps, the membranes were incubated with IRDye 800CW labelled goat-anti-rabbit and IRDye 680RD labelled goat-anti-mouse secondary antibodies (1:10,000; Li-Cor Biosciences) diluted in PBS containing 1% (w/v) skimmed milk powder and 0.1% (v/v) Tween 20. Coomassie-stained gels and Western/dot blots were imaged and quantified with the Li-Cor Odyssey system. Images of Coomassie-stained gels were also captured using an iBright CL1000 (Invitrogen).

### Fluorescence microscopy

Parasites were fixed in a final concentration of 4% w/v formaldehyde and 0.05% v/v glutaraldehyde overnight at 4 °C. After fixation, the cells were washed twice with PBS and stained with 1 µg/ml of DAPI immediately before imaging. The cells were imaged using an iMIC widefield fluorescence microscope (FEI Photonics, Germany) fitted with a CCD camera (Sensicam qe, pixel size 6.45 μm, PCO, Germany) using a 100x (NA 1.4) objective (Olympus, Germany) and the filter cubes ET-GFP and DAPI (Chroma Technology CORP, USA). The set up was controlled by the Live Acquisition software (FEI Photonics, Germany). Alternatively, trypanosomes were viewed with an automated DMI6000B wide field fluorescence microscope (Leica Microsystems, Germany) equipped with a DFC365FX camera (pixel size 6.45 µm) and a 100x oil objective (NA 1.4). The images are displayed as maximum intensity projections of Z-stacks. Image analysis was carried out using Fiji [78].

### Immunofluorescence microscopy

4 x 10^6^ parasites were harvested by centrifugation at 1,400 x*g* and 4 °C for 10 min and washed once in FCS free HMI-9. Cells were then fixed in a final concentration of 4% w/v formaldehyde for 30 minutes at room temperature. The fixative was removed by washing with vPBS (PBS supplemented with 46 mM sucrose and 10 mM glucose) and the resulting cell pellet was resuspended in 120 µl PBS. Per analysis 30 µl of the cell suspension was applied to poly-L-lysine coated slides and left to settle for 1 h in a humidifying chamber. Following a wash step with PBS, incubation for 10 min in PBS with 100 mM Tris-HCl and another PBS wash cells were blocked for 1 h with 1% BSA in PBS. Cells were then incubated for 1 h with primary antibodies (rabbit anti-VSG221 (1:100) or mouse anti-TY1 (1:500, BB2)) in 0.1% BSA in PBS. Excess antibodies were removed by washing in PBS before incubation with secondary antibodies (Alexa Fluor 488 goat anti-rabbit or Alexa Fluor 594 goat anti-mouse (both 1:500, Thermo Fisher Scientific)) in 0.1% BSA in PBS. Following removal of excess secondary antibodies by washes with PBS cells were incubated for 5 min at room temperature and in the dark with 0.5 µg/ml DAPI to stain kinetoplasts and nuclei. After a final wash step with PBS, cells were mounted with 80% glycerol in PBS. The cells were imaged using an iMIC widefield fluorescence microscope (FEI Photonics, Germany) as described above.

### LR White smFISH

Localisation of *VSG* mRNA molecules in relation to the ER was achieved by correlative single molecule FISH and immunofluorescence on LR White embedded trypanosome samples as described originally in Kramer et al. [48]. Around 4 x 10^7^ trypanosomes were harvested from cell culture and high pressure frozen before freeze substitution and LR White embedding. Sections of 100 nm thickness were generated and collected onto poly-L-lysine coated slides. Affymetrix smFISH, immunofluorescence staining and contrasting were performed sequentially. Affymetrix smFISH was performed following the protocol supplied with the QuantiGene ViewRNA ISH Cell Assay Kit (Glass Slide Format; Thermo Fisher Scientific) and modifications described in Kramer et al. [48]. The Affymetrix Probes used, targeted the mRNA of *VSG221*, *ILDat2.1* and *tubulin* and probes were generated based on the entire open reading frames with the regions coding for the ER-import signal and GPI-signal omitted for ILDat2.1. The sequences used for probe design are shown in S2 Table. Slides were stored in PBS overnight prior to immunofluorescence staining. Slides were incubated for 15 min each in 20 mM glycine PBS and then 1% BSA in PBS prior to incubation with the primary antibody rabbit anti-BiP (1:10,000; kind gift from J. Bangs) in 0.1% BSA in PBS for 2 h. Following four 5 min washes with PBS, the samples were incubated for 40 min with the secondary antibody Alexa Fluor 568 (1:250 dilution) and 5 µg/ml Hoechst in 0.1% BSA in PBS. Cells were then washed again 4 times for 5 min with PBS before embedding in ProLong Diamond Antifade (Invitrogen).

For quantitative mRNA to ER proximity analysis, slides were imaged using a DMI8 inverted widefield microscope (Thunder Imager, Leica Microsystems) with an HCX PL APO CS objective (100x, NA = 1.4, Leica Microsystems) using Type F immersion oil (refractive index = 1.518, Leica Microsystems). The microscope was controlled by the LAS-X software (Leica Microsystems). Samples were illuminated with an LED8 light source (Leica Microsystems). Excitation light was selected by using the filter sets: EX 462–496 nm; DC 500 nm; EM 506–532 (Alexa Fluor 488); EX 566–590 nm; DC 598 nm; EM602–680 nm (Alexa Fluor 568); EX 622–654 nm; DC 660 nm; EM 666–724 nm (Alexa Fluor 647); EX 374–407 nm; DC 415 nm; EM 420–450 nm (Hoechst). Image stacks containing 15 slices were captured using a K5 sCMOS camera (6.5 µm pixel size, Leica Microsystems).

Data were analysed with the aid of Fiji [78]. mRNA signals (*VSG221* and *α*-*tubulin* or *VSG221* and *ILDat2.1*) were localised on the in-focus plane of the acquired stack with the “find maxima” function and saved in the ROI manager. Intensities of the BIP signal were then measured at these locations and plotted together for each image acquired. For this, the intensities were sorted from low to high and plotted relative to the most intense signal. As the amount of mRNA signals for the pair of mRNAs analysed varied in an image, particles were plotted as particle distribution where particle numbers were normalised to the total number present. This allows for a pairwise comparison of the measured signal intensities.

For image generation, data were acquired on an Elyra S.1 structured illumination microscope (Zeiss), equipped with a 63x oil immersion objective and an sCMOS-Camera (PCO Edge 5.5). Following acquisition of fluorescence images, the samples were prepared for acquisition of electron micrographs. First, remaining immersion oil was removed with 100% EtOH. Then, slides were incubated overnight in ultra-pure water in order to be able to remove the cover slips. Samples were then dried and the slides cut with a diamond pen to smaller pieces that could be mounted into the scanning electron microscope. The samples were then contrasted by incubation for 10 min in 2% aqueous uranyl acetate and, followed by three washes with water, incubation for 5 min with 50% Reynolds’ lead citrate [79] in water and two subsequent washes with water. After drying, the samples were imaged with a field emission scanning electron microscope (JEOL JSM-7500F) employing a LABE (low-angle backscattered electron) detector. Inversion of the acquired data leads to TEM-like contrasted images as shown. For a detailed description of this detection method see [80]. Inkscape was used to manually correlate fluorescent images with the electron micrographs using the nuclei and kinetoplasts as intrinsic landmarks [80].

### Cell cycle analyses

For cell cycle analyses 1 x 10^7^ cells were harvested at 0, 8 and 24 h of induction with tetracycline by centrifugation at 1,400 x*g* and 4 °C for 10 min. The parasites were washed once in ice-cold TDB (5 mM KCl, 80 mM NaCl, 1 mM MgSO_4_, 20 mM Na_2_HPO_4_, 2 mM NaH_2_PO_4_, 20 mM glucose, pH 7.6), adjusted to a concentration of 1 x 10^8^ cells/ml and incubated with 10 µM ATTO 488 NHS-ester (ATTO-TEC GmbH) for 15 min on ice and in the dark. The cells were then washed three times with ice-cold TDB to remove any unbound dye and subsequently fixed in a final concentration of 2% w/v formaldehyde for 30 min at room temperature. After fixation, the cells were washed once with TDB and applied to a poly-L-lysine coated coverslip by centrifugation at 750 x*g* and room temperature for 1 min. Cells were mounted using Fluoromount-G with DAPI (Invitrogen). Slides were analysed directly using a DMI8 inverted widefield microscope (Thunder Imager, Leica Microsystems) or images were acquired for subsequent analysis as described above in the LR White smFISH section. Excitation light was selected by using the filter sets: EX 462–496 nm; DC 500 nm; EM 506–532 (ATTO 488); and EX 374–407 nm; DC 415 nm; EM 420–450 nm (DAPI). Image stacks containing 20 slices were captured using a K5 sCMOS camera (6.5 µm pixel size, Leica Microsystems).

### CB-5083 treatment of cells

Cells were induced with 1 µg/ml tetracycline to express either 121^SP^.ILDat2.1^tet^ (24 h) or LS.GFP-198 (4 h) before addition of 10 µg/ml CB-5083 in DMSO (#S8101, Selleckchem) for 4 h. Whole cell protein lysates were prepared and analysed as described under protein analyses. For Western and dot blot analysis of LS.GFP-198 a rabbit anti-GFP antibody (1:2,000, Invitrogen A11122) was used alongside a mouse monoclonal anti-PFR antibody (L13D6) (1:20; kind gift from P. Bastin).

### *T. brucei* cell volume distribution measurements

Cell volumes were analysed at 0 h, 8 h and 24 h after induction of expression with tetracycline with a Coulter counter Multisizer 4e equipped with a 50 µm aperture tube (Beckman Coulter GmbH, Germany). Cells were cultured in 0.2 µm filtered medium to avoid clogging of the aperture. At each time point, a total of 30,000 cells were analysed in a size range of 3.0 to 30 µm equivalent spherical diameter (ESD). Data are shown as mean ±SD of three (121^SP^.ILDat2.1^tet^) or two independent clones (LS.GFP-198).

## Supporting information

S1 Fig Cumulative growth curve of the cell line expressing ILDat1.2 VSG, in which the native GPI signal was replaced with that from *T. brucei* VSG MITat1.11. The growth was analysed for five days in the presence and absence of tetracycline. The parental 13–90 cells served as a control. Three independent clones were analysed, and data are presented as mean ± standard deviation (SD).
(PNG)

S2 Fig *ILDat2.1* mRNA is only efficiently transported to the ER when it contains a *bona fide* *T. brucei* VSG ER import signal. **(A)** Example showing individual electron microscopy images (inverted SEM, labelled EM) and overlays of the EM image with correlative light data (smFISH and immunofluorescence) of LR White embedded trypanosomes showing the localisation of BiP protein (white), *VSG221* mRNA (yellow), *α-tubulin* mRNA (magenta) and an overlay of all three fluorescence images and the EM image in the 121^SP^:ILDat2.1^tet^ and the ILDat2.1^tet^ cell lines. Scale bars: 2 µm. The graphs show pairwise comparative quantitative analyses of mRNA proximity to the ER (BiP signal) for *VSG221* (yellow) and *α-tubulin* (magenta) mRNA. The intensity of the BiP signal at the respective location of individual mRNA molecules of *VSG221* and *α-tubulin* is shown. The number (N) of individual mRNA molecules analysed are shown. Intensities are plotted sorted from lowest to highest intensity with the intensities given relative to the most intense BiP signal and the particles plotted normalised to the total amount of particles analysed in each data set (particle distribution) to allow comparison of the two curves. **(B)** Example showing individual electron microscopy images (inverted SEM, labelled EM) and overlays of the EM image with correlative light data (smFISH and immunofluorescence) of LR White embedded trypanosomes showing the localisation of BiP protein (white), *VSG221* mRNA (yellow), *ILDat2.1* mRNA (magenta) and an overlay of all three fluorescence images and the EM image in the 121^SP^:ILDat2.1^tet^ and ILDat2.1^tet^ cell lines. Scale bars: 2 µm. The graphs show pairwise comparative quantitative analyses of mRNA proximity to the ER (BiP signal) for *VSG221* (yellow) and *ILDat2.1* (magenta) mRNA. The intensity of the BiP signal at the respective location of individual mRNA molecules of *VSG221* and *ILDat2.1* is shown. The number (N) of individual mRNA molecules analysed are shown. Intensities are plotted sorted from lowest to highest intensity with the intensities given relative to the most intense BiP signal and the particles plotted normalised to the total amount of particles analysed in each data set (particle distribution) to allow comparison of the two curves. The overlays and graphs are identical with the images shown in Fig 4.
(PNG)

S3 Fig ILDat2.1 with a *bona fide* ER import signal is localised to the ER. **(A)** Comparative analysis of mRNA proximity to the ER (BiP signal) for *VSG221* (yellow) and *ILDat2.1* (magenta) (top graphs), and for *VSG221* (yellow) and *α-tubulin* (magenta) mRNA (bottom graphs) in the ILDat2.1^tet^ cell line. The graph shows the intensity of the BiP signal at the respective location of individual mRNA molecules of *VSG221* and *α-tubulin* or *ILDat2.1*. The number of individual mRNA molecules (N) analysed are shown. Intensities are plotted sorted from lowest to highest with the intensities given relative to the most intense BiP signal and the particles plotted are normalised to the total amount of particles analysed in each data set (particle distribution) to allow comparison of the two curves. **(B**) Comparative analysis of mRNA proximity to the ER (BiP signal) for *VSG221* (yellow) and *ILDat2.1* (magenta) (top graphs), and for *VSG221* (yellow) and *α-tubulin* (magenta) mRNA (bottom graphs) in the 121^SP^:ILDat2.1^tet^ cell line. The graphs show the intensity of the BiP signal at the respective location of individual mRNA molecules of *VSG221* and *α-tubulin* or *ILDat2.1*. The number of individual mRNA molecules (N) analysed are shown. Intensities are plotted sorted from lowest to highest with the intensities given relative to the most intense BiP signal and the particles plotted are normalised to the total amount of particles analysed in each data set (particle distribution) to allow comparison of the two curves.
(PNG)

S4 Fig Expression of VSG121 from downstream of the active BES-resident VSG221. **(A)** Quantification of *VSG221* and *VSG121* mRNA and **(B)** VSG221 and VSG121 protein expression in the 221^ES^.121 cell line. *VSG* mRNA and protein were normalised to *tubulin* and PFR protein, respectively. Expression levels are presented as percentages relative to VSG expression in the parental 13–90 cells and wild-type VSG121 for VSG221 and VSG121, respectively. Values are given as means and the error bars show the SD of three independent clones.
(PNG)

S5 Fig Treatment of 121^SP^:ILDat2.1-expressing cells with CB-5083. **(A)** Coomassie-stained gel showing 121^SP^:ILDat2.1 expression of tetracycline induced cells treated with CB-5083 (+) or DMSO as a control (-). **(B, C)** Tentative relative quantification of 121^SP^:ILDat2.1 expression based on the Coomassie gel shown in **(A)** with Tubulin used for normalisation. **(B)** Regions used to extract band intensities for tubulin and ILDat2.1 are marked in the gel. **(C)** No clear increase in expression of ILDat2.1 could be observed with fold changes between untreated and CB-5083 treated cells shown for the three clones analysed. As the quantification is based on Coomassie staining due to the absence of a specific antibody, the results should be interpreted cautiously.
(PNG)

S6 Fig CB-5083 treatment increases protein levels of ER-targeted GFP. **(A)** Western blot analysis showing LS.GFP-198 expression after treatment with CB-5083 (+) or treatment with DMSO as a control (-). PFR served as loading control. **(B)** Dot blot for quantification of GFP with and without CB-5083 treatment. **(C)** Fold change in GFP expression between untreated and CB-5083 treated cells.
(PNG)

S7 Fig Cell volume distributions of cells at different time points of induction of 121SP:ILDat2.1 and ER-targeted GFP expression. **(A, B)** Cell volume distributions were measured with a Coulter counter at 0 h, 8 h and 24 h of tetracycline induction. Data represent 30,000 cells per measurement and are shown as mean ± SD. **(A)** The graph is based on the analysis of three independent 121^SP^:ILDat2.1^tet^ clones. **(B)** The graph is based on the analysis of two independent LS.GFP-198 clones.
(PNG)

S8 Fig Overview images of cells at 0 h, 8 h and 24 h after induction of expression of LS.GFP-198 cells. **(A-C)** Composite immunofluorescence images of cells labelled with ATTO 488 NHS-ester (shown in magenta) and DAPI (shown in cyan). **(A)** 0 h time point. **(B)** 8 h time point. **(C)** 24 h time point. Scale bar: 20 µm.
(PNG)

S1 Table Full coding sequences of proteins analysed in this study. Full coding sequences of all expressed proteins in this study are shown from start to stop codon.
(DOCX)

S2 Table Sequence regions used for Affymetrix probe design. Full coding sequences of transcripts targeted by Affymetrix probes are shown from start to stop codon. Sequence regions excluded from probe design are indicated in grey. The added start codon in *ILDat2.1* is underlined.
(DOCX)

S3 Table The grand average hydropathy (GRAVY) scores of select ER import signals. The reported ILDat2.1 ER import signal is hydrophilic. The GRAVY scores were computed using Expasy ProtParam (https://web.expasy.org/protparam). A positive or negative GRAVY score indicates that the signal peptide is hydrophobic or hydrophilic, respectively. The hydrophobic core of the signal sequence (underlined) was predicted using Phobius (https://www.ebi.ac.uk/Tools/pfa/phobius/). The reported ILDat2.1 ER import signal peptide (Gardiner et al., 1996 [44]) could not be predicted by Phobius.
(DOCX)

S1 Raw images Raw Coomassie gels, Western blots and dot blots. (PDF)

S1 Data Excel file containing all data tables for graphs shown in the manuscript. (XLSX)

## References

[1] Engstler M, Pfohl T, Herminghaus S, Boshart M, Wiegertjes G, Heddergott N, et al. Hydrodynamic flow-mediated protein sorting on the cell surface of trypanosomes. Cell. 2007;131(3):505–15. doi: 10.1016/j.cell.2007.08.046 17981118

[2] Mugnier MR, Cross GAM, Papavasiliou FN. The in vivo dynamics of antigenic variation in *Trypanosoma brucei*. Science. 2015;347(6229):1470–3. doi: 10.1126/science.aaa4502 25814582 PMC4514441

[3] Pays E, Vanhamme L, Pérez-Morga D. Antigenic variation in *Trypanosoma brucei*: facts, challenges and mysteries. Curr Opin Microbiol. 2004;7(4):369–74. doi: 10.1016/j.mib.2004.05.001 15288623

[4] Kreil G. Transfer of proteins across membranes. Annu Rev Biochem. 1981;50:317–48. doi: 10.1146/annurev.bi.50.070181.001533 7023361

[5] McConnell J, Gurnett AM, Cordingley JS, Walker JE, Turner MJ. Biosynthesis of *Trypanosoma brucei* variant surface glycoprotein. I. Synthesis, size, and processing of an N-terminal signal peptide. Mol Biochem Parasitol. 1981;4(3–4):225–42. doi: 10.1016/0166-6851(81)90021-9 6120455

[6] Hegde RS, Bernstein HD. The surprising complexity of signal sequences. Trends Biochem Sci. 2006;31(10):563–71. doi: 10.1016/j.tibs.2006.08.004 16919958

[7] Kunze M, Berger J. The similarity between N-terminal targeting signals for protein import into different organelles and its evolutionary relevance. Front Physiol. 2015;6:259. doi: 10.3389/fphys.2015.00259 26441678 PMC4585086

[8] Nielsen H, Engelbrecht J, von Heijne G, Brunak S. Defining a similarity threshold for a functional protein sequence pattern: the signal peptide cleavage site. Proteins. 1996;24(2):165–77. doi: 10.1002/(SICI)1097-0134(199602)24:2<165::AID-PROT4>3.0.CO;2-I 8820484

[9] von Heijne G. Patterns of amino acids near signal-sequence cleavage sites. Eur J Biochem. 1983;133(1):17–21. doi: 10.1111/j.1432-1033.1983.tb07424.x 6852022

[10] Owji H, Nezafat N, Negahdaripour M, Hajiebrahimi A, Ghasemi Y. A comprehensive review of signal peptides: Structure, roles, and applications. Eur J Cell Biol. 2018;97(6):422–41. doi: 10.1016/j.ejcb.2018.06.003 29958716

[11] Ng DT, Brown JD, Walter P. Signal sequences specify the targeting route to the endoplasmic reticulum membrane. J Cell Biol. 1996;134(2):269–78. doi: 10.1083/jcb.134.2.269 8707814 PMC2120870

[12] Chen Q, Jagannathan S, Reid DW, Zheng T, Nicchitta CV. Hierarchical regulation of mRNA partitioning between the cytoplasm and the endoplasmic reticulum of mammalian cells. Mol Biol Cell. 2011;22(14):2646–58. doi: 10.1091/mbc.E11-03-0239 21613539 PMC3135488

[13] Reid DW, Nicchitta CV. Primary role for endoplasmic reticulum-bound ribosomes in cellular translation identified by ribosome profiling. J Biol Chem. 2012;287(8):5518–27. doi: 10.1074/jbc.M111.312280 22199352 PMC3285328

[14] Reid DW, Nicchitta CV. Diversity and selectivity in mRNA translation on the endoplasmic reticulum. Nat Rev Mol Cell Biol. 2015;16(4):221–31. doi: 10.1038/nrm3958 25735911 PMC4494666

[15] Nilsson I, Lara P, Hessa T, Johnson AE, von Heijne G, Karamyshev AL. The code for directing proteins for translocation across ER membrane: SRP cotranslationally recognizes specific features of a signal sequence. J Mol Biol. 2015;427(6 Pt A):1191–201. doi: 10.1016/j.jmb.2014.06.014 24979680 PMC4277940

[16] Boothroyd JC, Paynter CA, Cross GA, Bernards A, Borst P. Variant surface glycoproteins of *Trypanosoma brucei* are synthesised with cleavable hydrophobic sequences at the carboxy and amino termini. Nucleic Acids Res. 1981;9(18):4735–43. doi: 10.1093/nar/9.18.4735 6272213 PMC327471

[17] Cross GA. Structure of the variant glycoproteins and surface coat of *Trypanosoma brucei*. Philos Trans R Soc Lond B Biol Sci. 1984;307(1131):3–12. doi: 10.1098/rstb.1984.0104 6151686

[18] Johnson PJ, Kooter JM, Borst P. Inactivation of transcription by UV irradiation of *T. brucei* provides evidence for a multicistronic transcription unit including a VSG gene. Cell. 1987;51(2):273–81. doi: 10.1016/0092-8674(87)90154-1 3664637

[19] Siegel TN, Gunasekera K, Cross GAM, Ochsenreiter T. Gene expression in *Trypanosoma brucei*: lessons from high-throughput RNA sequencing. Trends Parasitol. 2011;27(10):434–41. doi: 10.1016/j.pt.2011.05.006 21737348 PMC3178736

[20] De Lange T, Berkvens TM, Veerman HJ, Frasch AC, Barry JD, Borst P. Comparison of the genes coding for the common 5’ terminal sequence of messenger RNAs in three trypanosome species. Nucleic Acids Res. 1984;12(11):4431–43. doi: 10.1093/nar/12.11.4431 6204273 PMC318848

[21] Campbell DA, van Bree MP, Boothroyd JC. The 5’-limit of transposition and upstream barren region of a trypanosome VSG gene: tandem 76 base-pair repeats flanking (TAA)90. Nucleic Acids Research. 1984;12(6):2759–74. doi: 10.1093/nar/12.6.2759 6324125 PMC318704

[22] Michaeli S. Trans-splicing in trypanosomes: machinery and its impact on the parasite transcriptome. Future Microbiol. 2011;6(4):459–74. doi: 10.2217/fmb.11.20 21526946

[23] Clayton CE. Life without transcriptional control? From fly to man and back again. EMBO J. 2002;21(8):1881–8. doi: 10.1093/emboj/21.8.1881 11953307 PMC125970

[24] Clayton C. Regulation of gene expression in trypanosomatids: living with polycistronic transcription. Open Biol. 2019;9(6):190072. doi: 10.1098/rsob.190072 31164043 PMC6597758

[25] Kramer S. Developmental regulation of gene expression in the absence of transcriptional control: the case of kinetoplastids. Mol Biochem Parasitol. 2012;181(2):61–72. doi: 10.1016/j.molbiopara.2011.10.002 22019385

[26] Cross GAM, Kim H-S, Wickstead B. Capturing the variant surface glycoprotein repertoire (the VSGnome) of *Trypanosoma brucei* Lister 427. Mol Biochem Parasitol. 2014;195(1):59–73. doi: 10.1016/j.molbiopara.2014.06.004 24992042

[27] Hertz-Fowler C, Figueiredo LM, Quail MA, Becker M, Jackson A, Bason N, et al. Telomeric expression sites are highly conserved in *Trypanosoma brucei*. PLoS One. 2008;3(10):e3527. doi: 10.1371/journal.pone.0003527 18953401 PMC2567434

[28] Budzak J, Kerry LE, Aristodemou A, Hall BS, Witmer K, Kushwaha M, et al. Dynamic colocalization of 2 simultaneously active VSG expression sites within a single expression-site body in *Trypanosoma brucei*. Proc Natl Acad Sci U S A. 2019;116(33):16561–70. doi: 10.1073/pnas.1905552116 31358644 PMC6697882

[29] Navarro M, Gull K. A pol I transcriptional body associated with VSG mono-allelic expression in *Trypanosoma brucei*. Nature. 2001;414(6865):759–63. doi: 10.1038/414759a 11742402

[30] Sheader K, Vaughan S, Minchin J, Hughes K, Gull K, Rudenko G. Variant surface glycoprotein RNA interference triggers a precytokinesis cell cycle arrest in African trypanosomes. Proc Natl Acad Sci U S A. 2005;102(24):8716–21. doi: 10.1073/pnas.0501886102 15937117 PMC1150830

[31] Ooi C-P, Smith TK, Gluenz E, Wand NV, Vaughan S, Rudenko G. Blocking variant surface glycoprotein synthesis alters endoplasmic reticulum exit sites/Golgi homeostasis in *Trypanosoma brucei*. Traffic. 2018;19(6):391–405. doi: 10.1111/tra.12561 29533496 PMC6001540

[32] Muñoz-Jordán JL, Davies KP, Cross GA. Stable expression of mosaic coats of variant surface glycoproteins in *Trypanosoma brucei*. Science. 1996;272(5269):1795–7. doi: 10.1126/science.272.5269.1795 8650579

[33] Smith TK, Vasileva N, Gluenz E, Terry S, Portman N, Kramer S, et al. Blocking variant surface glycoprotein synthesis in *Trypanosoma brucei* triggers a general arrest in translation initiation. PLoS One. 2009;4(10):e7532. doi: 10.1371/journal.pone.0007532 19855834 PMC2762041

[34] Ridewood S, Ooi C-P, Hall B, Trenaman A, Wand NV, Sioutas G, et al. The role of genomic location and flanking 3’UTR in the generation of functional levels of variant surface glycoprotein in *Trypanosoma brucei*. Mol Microbiol. 2017;106(4):614–34. doi: 10.1111/mmi.13838 28906055 PMC5698767

[35] Batram C, Jones NG, Janzen CJ, Markert SM, Engstler M. Expression site attenuation mechanistically links antigenic variation and development in *Trypanosoma brucei*. Elife. 2014;3:e02324. doi: 10.7554/eLife.02324 24844706 PMC4027811

[36] Zimmermann H, Subota I, Batram C, Kramer S, Janzen CJ, Jones NG, et al. A quorum sensing-independent path to stumpy development in *Trypanosoma brucei*. PLoS Pathog. 2017;13(4):e1006324. doi: 10.1371/journal.ppat.1006324 28394929 PMC5398725

[37] Berberof M, Vanhamme L, Tebabi P, Pays A, Jefferies D, Welburn S, et al. The 3’-terminal region of the mRNAs for VSG and procyclin can confer stage specificity to gene expression in *Trypanosoma brucei*. EMBO J. 1995;14(12):2925–34. doi: 10.1002/j.1460-2075.1995.tb07292.x 7796818 PMC398412

[38] Viegas IJ, de Macedo JP, Serra L, De Niz M, Temporão A, Silva Pereira S, et al. N^6^-methyladenosine in poly(A) tails stabilize VSG transcripts. Nature. 2022;604(7905):362–70. doi: 10.1038/s41586-022-04544-0 35355019 PMC9150445

[39] Melo do Nascimento L, Egler F, Arnold K, Papavasiliou N, Clayton C, Erben E. Functional insights from a surface antigen mRNA-bound proteome. Elife. 2021;10:e68136. doi: 10.7554/eLife.68136 33783358 PMC8051951

[40] Maudlin IE, Kelly S, Schwede A, Carrington M. VSG mRNA levels are regulated by the production of functional VSG protein. Mol Biochem Parasitol. 2021;241:111348. doi: 10.1016/j.molbiopara.2020.111348 33352254 PMC7871013

[41] Barry JD, Gathuo H. Antigenic variation in *Trypanosoma vivax*: isolation of a serodeme. Parasitology. 1984;89 (Pt 1):49–58. doi: 10.1017/s0031182000001128 6206455

[42] Chamond N, Cosson A, Blom-Potar MC, Jouvion G, D’Archivio S, Medina M, et al. *Trypanosoma vivax* infections: pushing ahead with mouse models for the study of Nagana. I. Parasitological, hematological and pathological parameters. PLoS Negl Trop Dis. 2010;4(8):e792. doi: 10.1371/journal.pntd.0000792 20706595 PMC2919405

[43] Gardiner PR, Pearson TW, Clarke MW, Mutharia LM. Identification and isolation of a variant surface glycoprotein from *Trypanosoma vivax*. Science. 1987;235(4790):774–7. doi: 10.1126/science.3810164 3810164

[44] Gardiner PR, Nene V, Barry MM, Thatthi R, Burleigh B, Clarke MW. Characterization of a small variable surface glycoprotein from *Trypanosoma vivax*. Mol Biochem Parasitol. 1996;82(1):1–11. doi: 10.1016/0166-6851(96)02687-4 8943146

[45] Greif G, Ponce de Leon M, Lamolle G, Rodriguez M, Piñeyro D, Tavares-Marques LM, et al. Transcriptome analysis of the bloodstream stage from the parasite *Trypanosoma vivax*. BMC Genomics. 2013;14:149. doi: 10.1186/1471-2164-14-149 23497072 PMC4007602

[46] Jones NG, Engstler M. Structure-function analysis defines the minimal functional C-terminal domain of the variant surface glycoprotein of *Trypanosoma brucei*. J Biol Chem. 2025;301(7):110260. doi: 10.1016/j.jbc.2025.110260 40412524 PMC12226131

[47] Garrison P, Bangs JD. p97 Inhibitor CB-5083 Blocks ERAD in *Trypanosoma brucei*. Mol Biochem Parasitol. 2020;239:111313. doi: 10.1016/j.molbiopara.2020.111313 32735998

[48] Kramer S, Meyer-Natus E, Stigloher C, Thoma H, Schnaufer A, Engstler M. Parallel monitoring of RNA abundance, localization and compactness with correlative single molecule FISH on LR White embedded samples. Nucleic Acids Res. 2021;49(3):e14. doi: 10.1093/nar/gkaa1142 33275141 PMC7897490

[49] Urwyler S, Studer E, Renggli CK, Roditi I. A family of stage-specific alanine-rich proteins on the surface of epimastigote forms of *Trypanosoma brucei*. Mol Microbiol. 2007;63(1):218–28. doi: 10.1111/j.1365-2958.2006.05492.x 17229212

[50] Roditi I, Carrington M, Turner M. Expression of a polypeptide containing a dipeptide repeat is confined to the insect stage of *Trypanosoma brucei*. Nature. 1987;325(6101):272–4. doi: 10.1038/325272a0 3808022

[51] Macleod OJS, Bart J-M, MacGregor P, Peacock L, Savill NJ, Hester S, et al. A receptor for the complement regulator factor H increases transmission of trypanosomes to tsetse flies. Nat Commun. 2020;11(1):1326. doi: 10.1038/s41467-020-15125-y 32165615 PMC7067766

[52] Liu HS, Jan MS, Chou CK, Chen PH, Ke NJ. Is green fluorescent protein toxic to the living cells?. Biochem Biophys Res Commun. 1999;260(3):712–7. doi: 10.1006/bbrc.1999.0954 10403831

[53] Borst P. Antigenic variation and allelic exclusion. Cell. 2002;109(1):5–8. doi: 10.1016/s0092-8674(02)00711-0 11955440

[54] Voss TS, Healer J, Marty AJ, Duffy MF, Thompson JK, Beeson JG, et al. A var gene promoter controls allelic exclusion of virulence genes in *Plasmodium falciparum* malaria. Nature. 2006;439(7079):1004–8. doi: 10.1038/nature04407 16382237

[55] Horn D. Antigenic variation in African trypanosomes. Mol Biochem Parasitol. 2014;195(2):123–9. doi: 10.1016/j.molbiopara.2014.05.001 24859277 PMC4155160

[56] Bakari-Soale M, Batram C, Zimmermann H, Jones NG, Engstler M. The dual role of the 16mer motif within the 3’ untranslated region of the variant surface glycoprotein of *Trypanosoma brucei*. bioRxiv. 2023. doi: 10.1101/2023.12.21.572740 PMC12763537 41247779

[57] Manna PT, Boehm C, Leung KF, Natesan SK, Field MC. Life and times: synthesis, trafficking, and evolution of VSG. Trends Parasitol. 2014;30(5):251–8. doi: 10.1016/j.pt.2014.03.004 24731931 PMC4007029

[58] Ast T, Cohen G, Schuldiner M. A network of cytosolic factors targets SRP-independent proteins to the endoplasmic reticulum. Cell. 2013;152(5):1134–45. doi: 10.1016/j.cell.2013.02.003 23452858

[59] Goldshmidt H, Sheiner L, Bütikofer P, Roditi I, Uliel S, Günzel M, et al. Role of protein translocation pathways across the endoplasmic reticulum in *Trypanosoma brucei*. J Biol Chem. 2008;283(46):32085–98. doi: 10.1074/jbc.M801499200 18768469

[60] von Heijne G. Life and death of a signal peptide. Nature. 1998;396(6707):111, 113. doi: 10.1038/24036 9823886

[61] Walter P, Johnson AE. Signal sequence recognition and protein targeting to the endoplasmic reticulum membrane. Annu Rev Cell Biol. 1994;10:87–119. doi: 10.1146/annurev.cb.10.110194.000511 7888184

[62] Levine CG, Mitra D, Sharma A, Smith CL, Hegde RS. The efficiency of protein compartmentalization into the secretory pathway. Mol Biol Cell. 2005;16(1):279–91. doi: 10.1091/mbc.e04-06-0508 15496459 PMC539172

[63] Martoglio B, Dobberstein B. Signal sequences: more than just greasy peptides. Trends Cell Biol. 1998;8(10):410–5. doi: 10.1016/s0962-8924(98)01360-9 9789330

[64] Duffy J, Patham B, Mensa-Wilmot K. Discovery of functional motifs in h-regions of trypanosome signal sequences. Biochem J. 2010;426(2):135–45. doi: 10.1042/BJ20091277 20017734

[65] Günzl A, Bruderer T, Laufer G, Schimanski B, Tu L-C, Chung H-M, et al. RNA polymerase I transcribes procyclin genes and variant surface glycoprotein gene expression sites in *Trypanosoma brucei*. Eukaryot Cell. 2003;2(3):542–51. doi: 10.1128/EC.2.3.542-551.2003 12796299 PMC161450

[66] Haimovich G, Medina DA, Causse SZ, Garber M, Millán-Zambrano G, Barkai O, et al. Gene expression is circular: factors for mRNA degradation also foster mRNA synthesis. Cell. 2013;153(5):1000–11. doi: 10.1016/j.cell.2013.05.012 23706738

[67] Sun M, Schwalb B, Schulz D, Pirkl N, Etzold S, Larivière L, et al. Comparative dynamic transcriptome analysis (cDTA) reveals mutual feedback between mRNA synthesis and degradation. Genome Res. 2012;22(7):1350–9. doi: 10.1101/gr.130161.111 22466169 PMC3396375

[68] Pérez-Ortín JE, de Miguel-Jiménez L, Chávez S. Genome-wide studies of mRNA synthesis and degradation in eukaryotes. Biochim Biophys Acta. 2012;1819(6):604–15. doi: 10.1016/j.bbagrm.2011.12.002 22182827

[69] Shalem O, Dahan O, Levo M, Martinez MR, Furman I, Segal E, et al. Transient transcriptional responses to stress are generated by opposing effects of mRNA production and degradation. Mol Syst Biol. 2008;4:223. doi: 10.1038/msb.2008.59 18854817 PMC2583085

[70] Sun M, Schwalb B, Pirkl N, Maier KC, Schenk A, Failmezger H, et al. Global analysis of eukaryotic mRNA degradation reveals Xrn1-dependent buffering of transcript levels. Mol Cell. 2013;52(1):52–62. doi: 10.1016/j.molcel.2013.09.010 24119399

[71] Abernathy E, Gilbertson S, Alla R, Glaunsinger B. Viral Nucleases Induce an mRNA Degradation-Transcription Feedback Loop in Mammalian Cells. Cell Host Microbe. 2015;18(2):243–53. doi: 10.1016/j.chom.2015.06.019 26211836 PMC4538998

[72] Singh P, James RS, Mee CJ, Morozov IY. mRNA levels are buffered upon knockdown of RNA decay and translation factors via adjustment of transcription rates in human HepG2 cells. RNA Biol. 2019;16(9):1147–55. doi: 10.1080/15476286.2019.1621121 31116665 PMC6693547

[73] Timmers HTM, Tora L. Transcript buffering: a balancing act between mRNA synthesis and mRNA degradation. Mol Cell. 2018;72(1):10–7. doi: 10.1016/j.molcel.2018.08.023 30290147

[74] Wirtz E, Leal S, Ochatt C, Cross GA. A tightly regulated inducible expression system for conditional gene knock-outs and dominant-negative genetics in *Trypanosoma brucei*. Mol Biochem Parasitol. 1999;99(1):89–101. doi: 10.1016/s0166-6851(99)00002-x 10215027

[75] Heckman KL, Pease LR. Gene splicing and mutagenesis by PCR-driven overlap extension. Nat Protoc. 2007;2(4):924–32. doi: 10.1038/nprot.2007.132 17446874

[76] Nielsen H. Predicting secretory proteins with SignalP. Methods in Molecular Biology. 2017;1611:59–73. doi: 10.1007/978-1-4939-7015-5_6 28451972

[77] Janzen CJ, Lander F, Dreesen O, Cross GAM. Telomere length regulation and transcriptional silencing in KU80-deficient *Trypanosoma brucei*. Nucleic Acids Res. 2004;32(22):6575–84. doi: 10.1093/nar/gkh991 15602000 PMC545459

[78] Schindelin J, Arganda-Carreras I, Frise E, Kaynig V, Longair M, Pietzsch T, et al. Fiji: an open-source platform for biological-image analysis. Nat Methods. 2012;9(7):676–82. doi: 10.1038/nmeth.2019 22743772 PMC3855844

[79] Reynolds ES. The use of lead citrate at high pH as an electron-opaque stain in electron microscopy. J Cell Biol. 1963;17(1):208–12. doi: 10.1083/jcb.17.1.208 13986422 PMC2106263

[80] Markert SM, Bauer V, Muenz TS, Jones NG, Helmprobst F, Britz S, et al. 3D subcellular localization with superresolution array tomography on ultrathin sections of various species. Methods Cell Biol. 2017;140:21–47. doi: 10.1016/bs.mcb.2017.03.004 28528634

