# Supplementary material for: Endoplasmic reticulum engagement of transcripts triggers VSG mRNA balancing in trypanosomes
Source: PLoS Pathog. 2026 Aug 18;22(8):e1014518. doi: 10.1371/journal.ppat.1014518 (PMC13515020; doi:10.1371/journal.ppat.1014518)
Supplement: Supplementary file 1 [file ppat.1014518.s012.pdf]

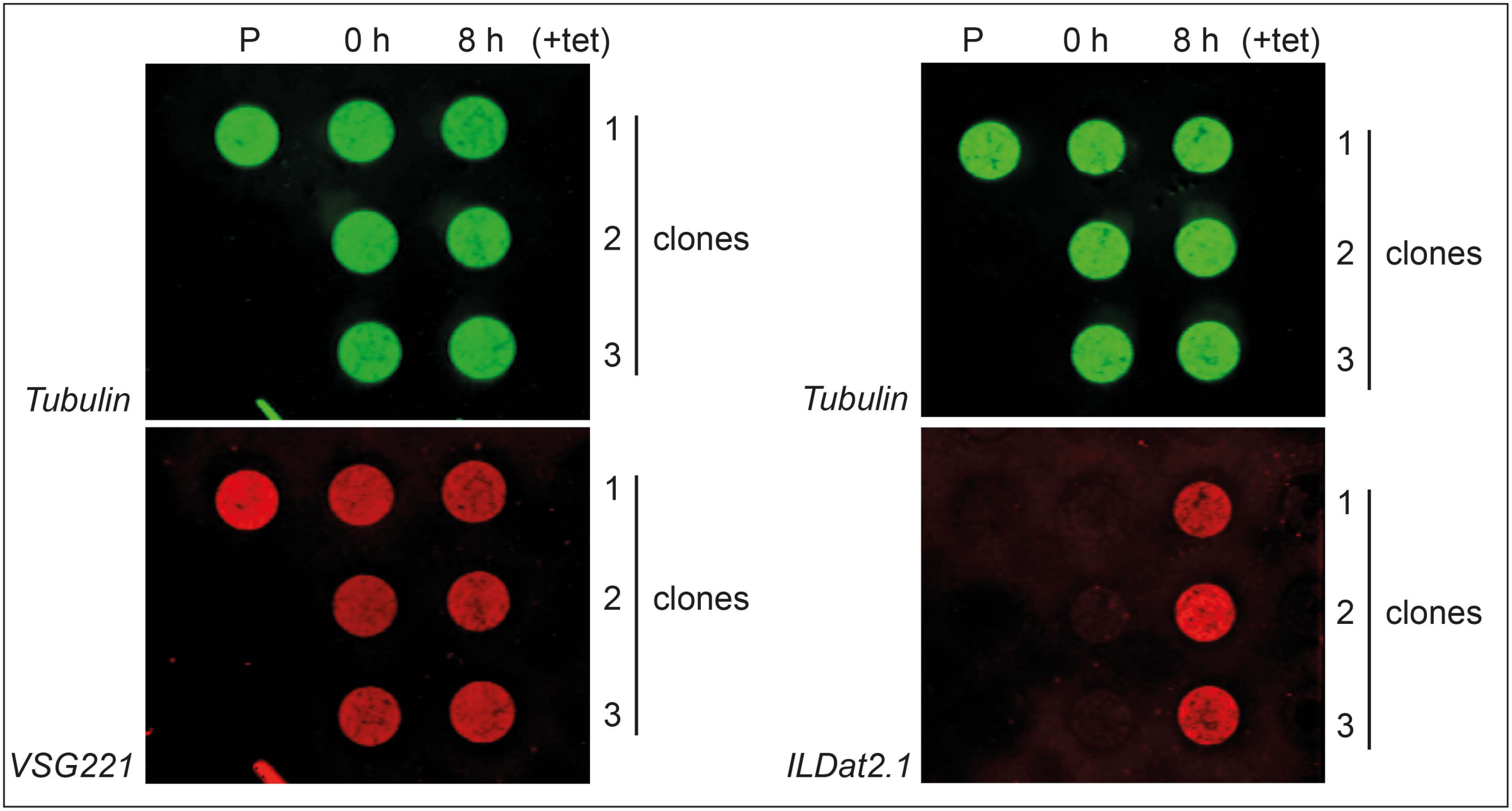

Figure 1C

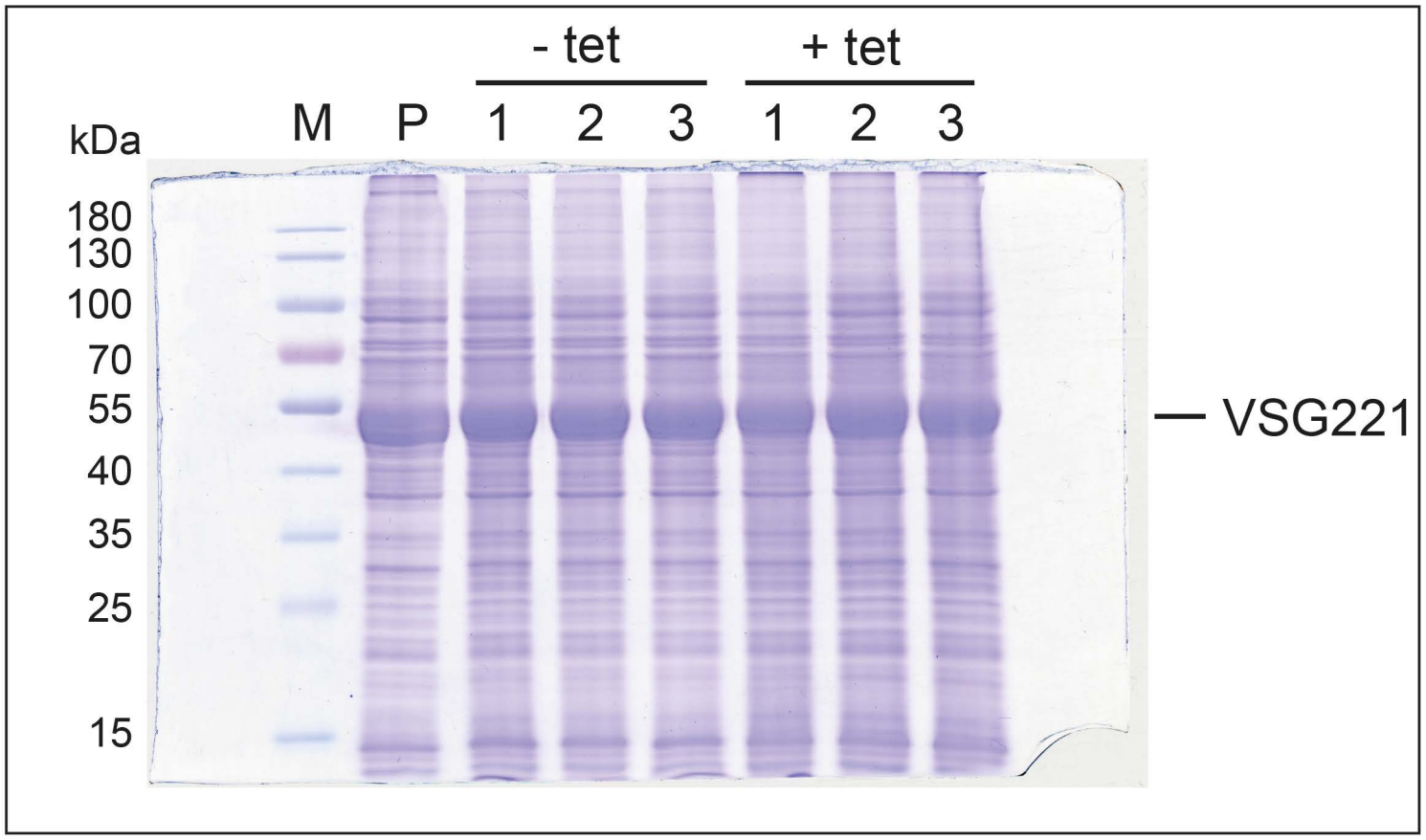

Figure 1D

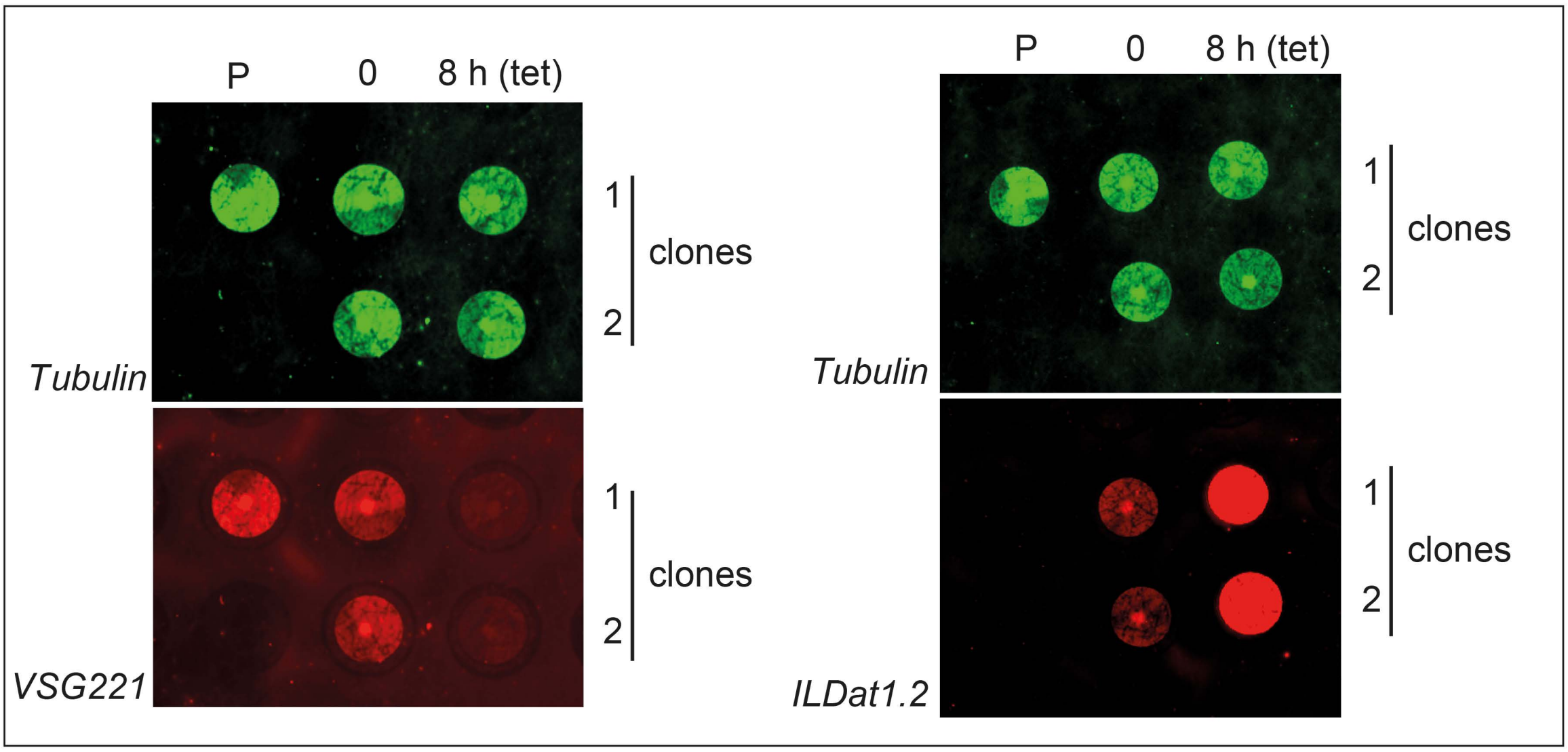

Figure 2B

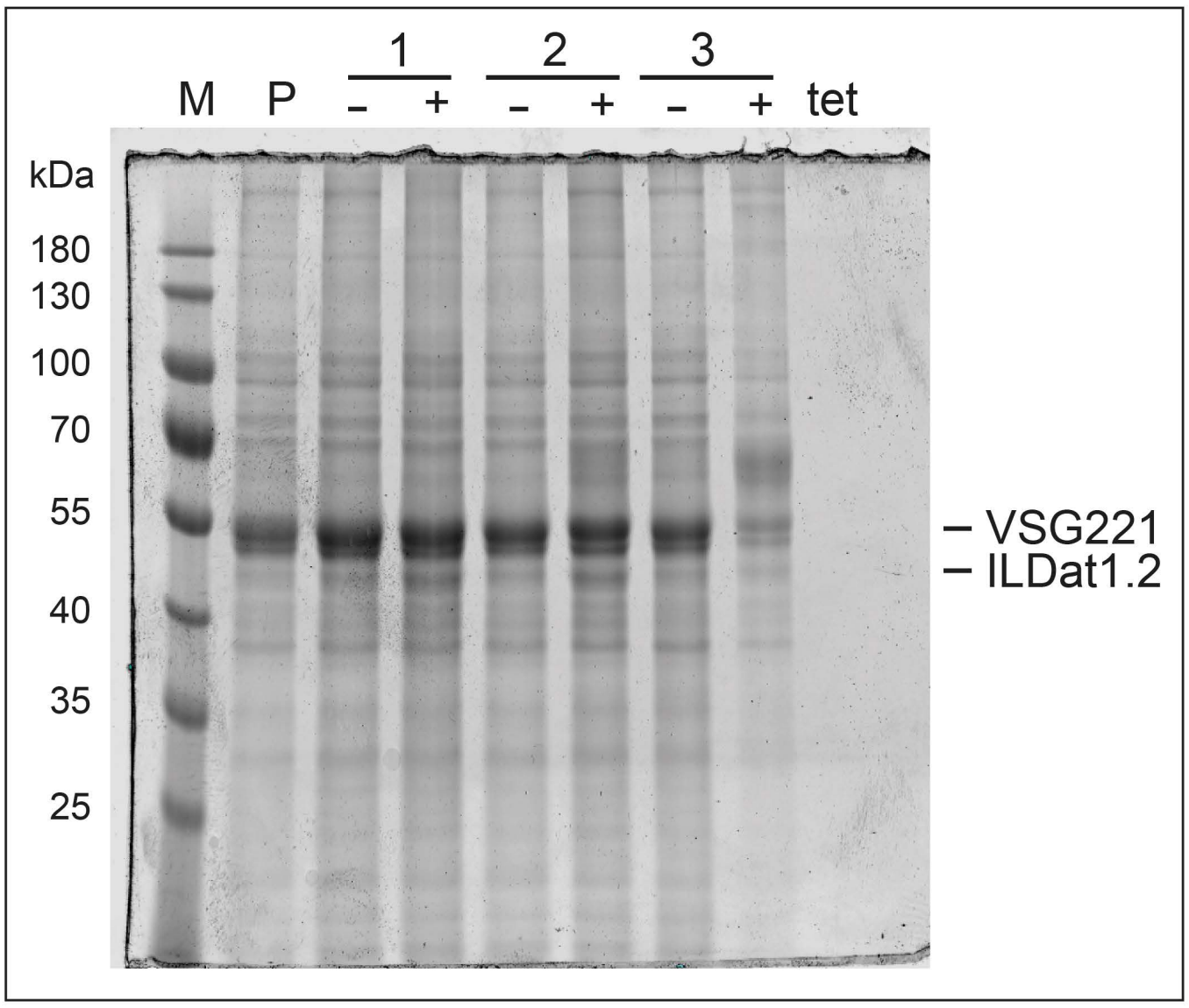

Figure 2C

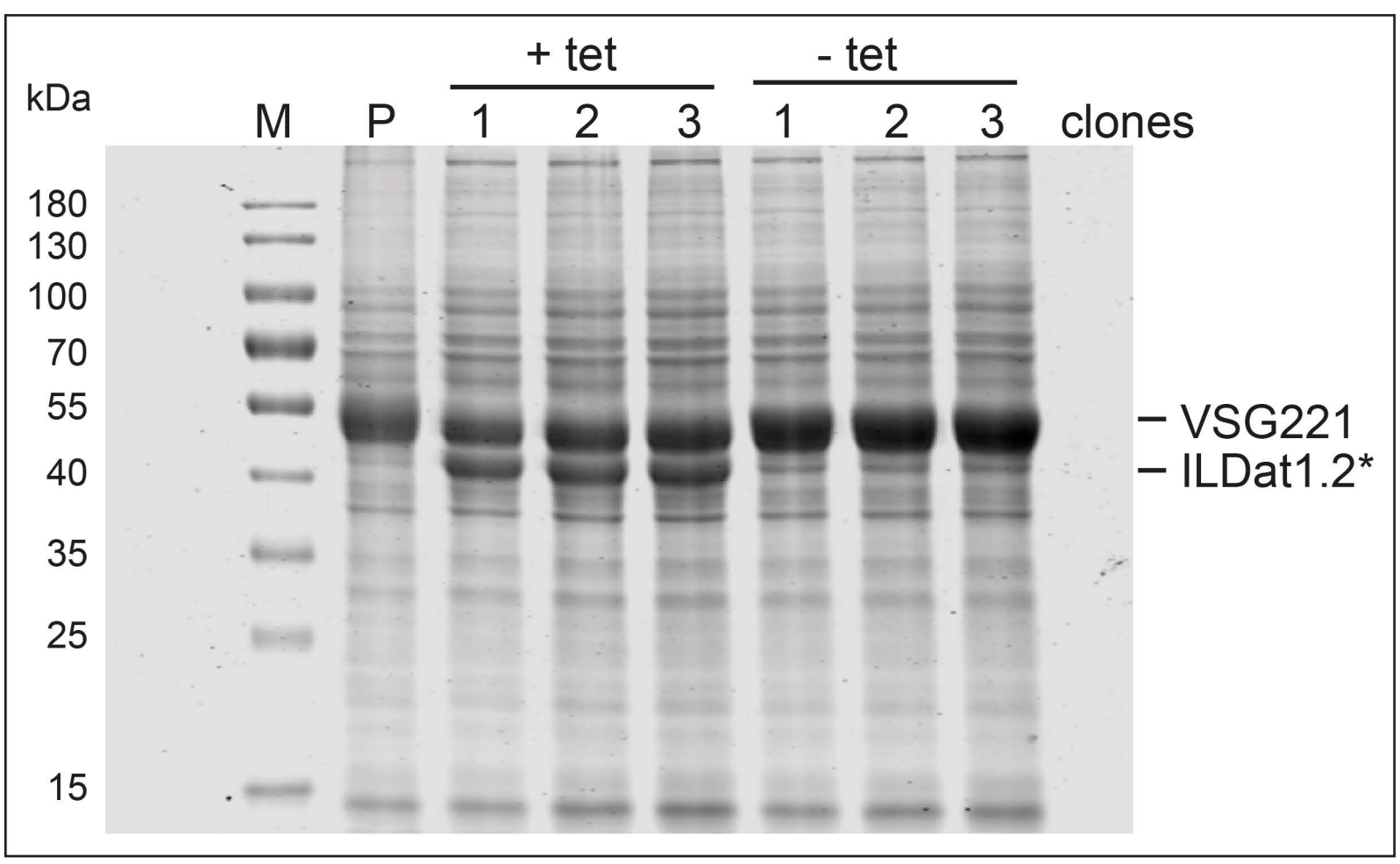

Figure 2D

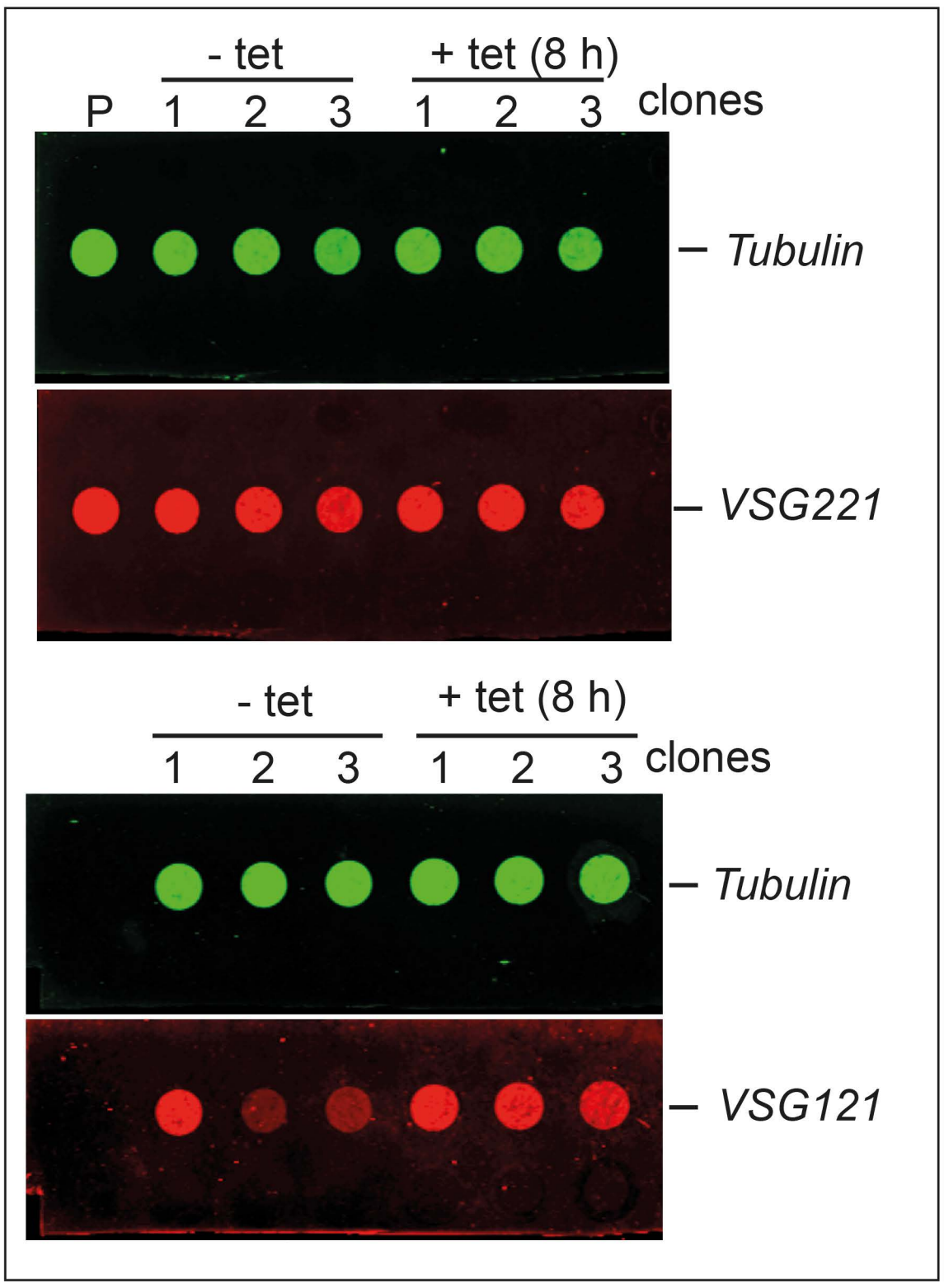

Figure 3C

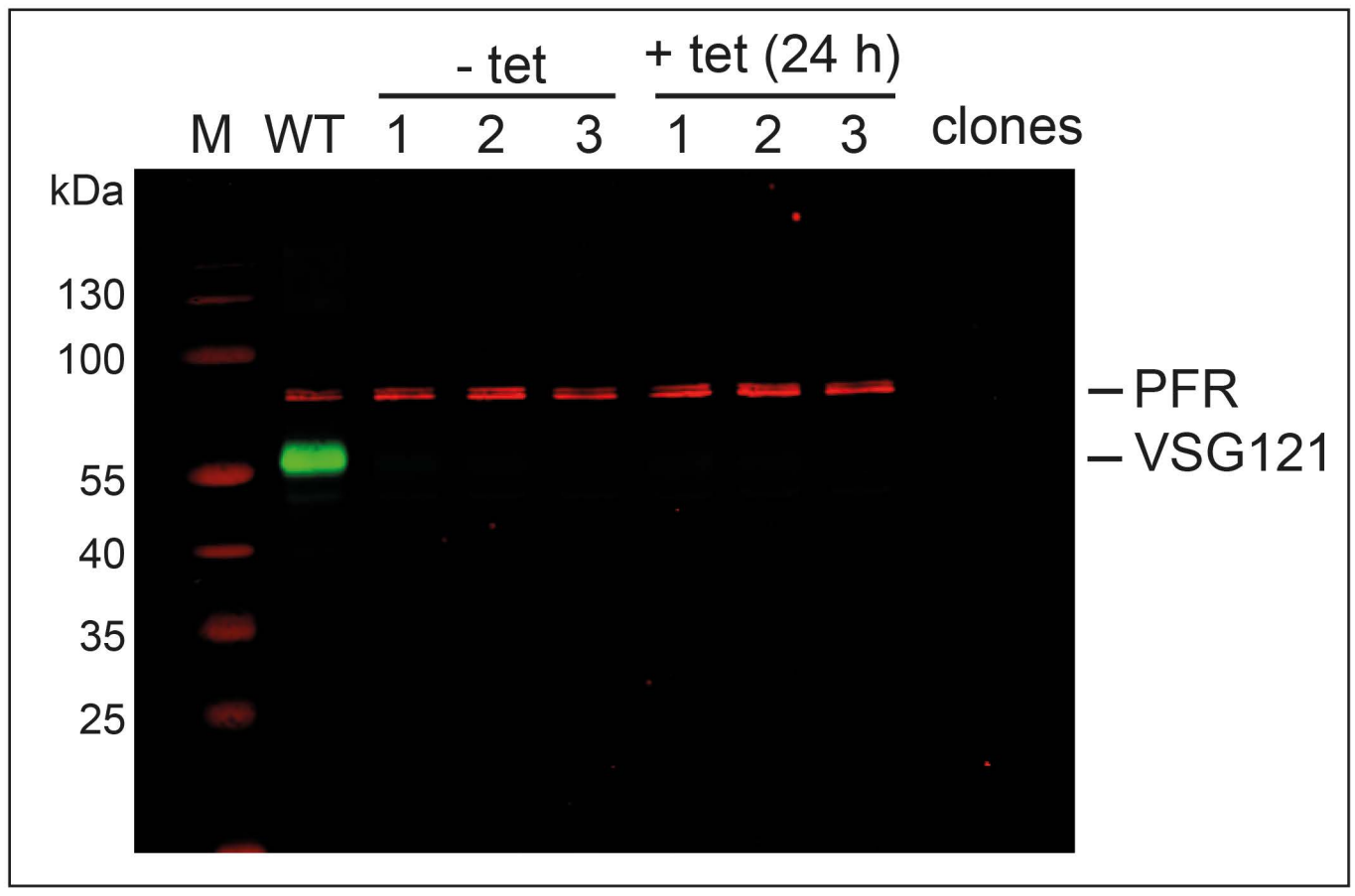

Figure 3D

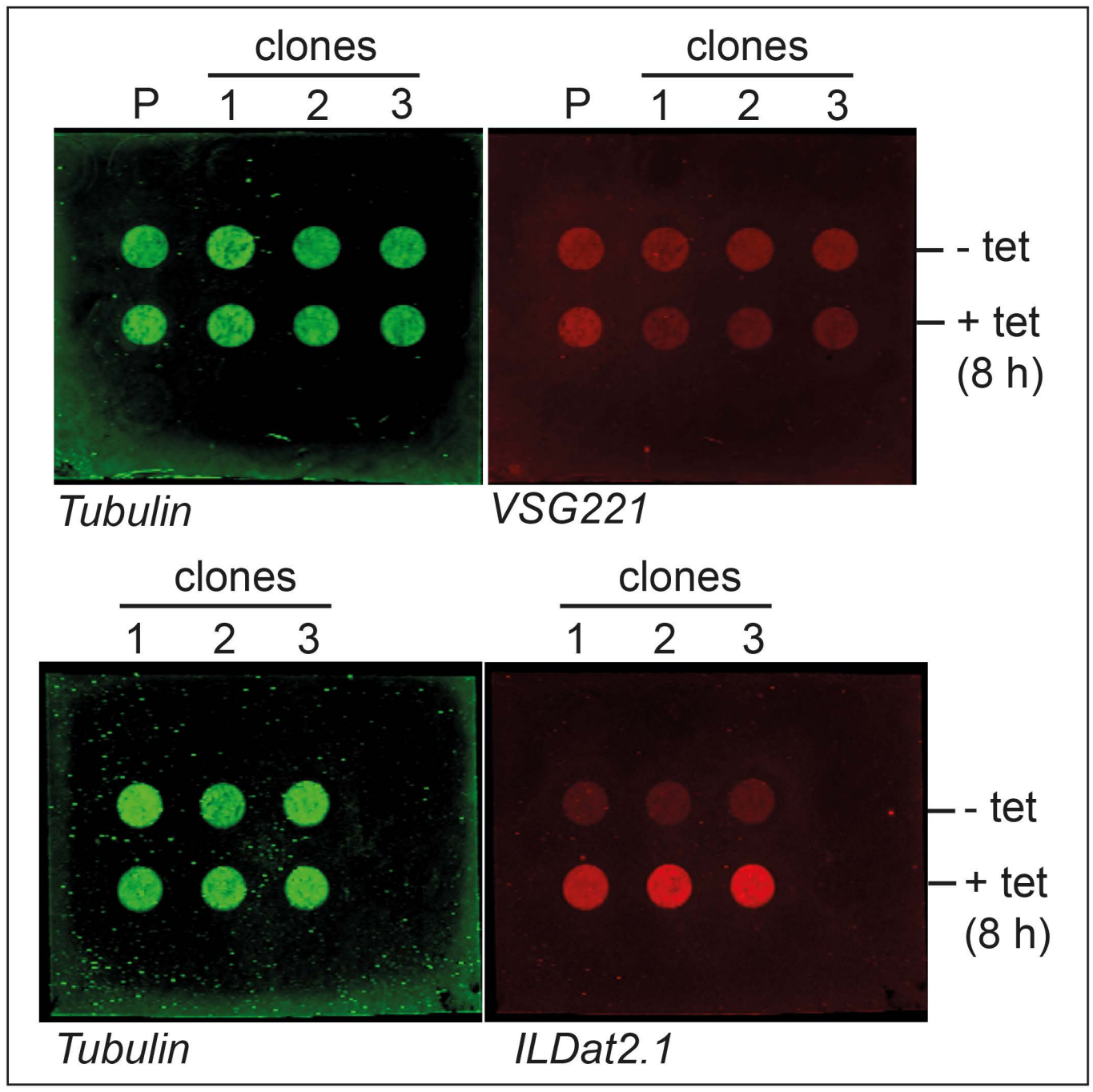

Figure 3E

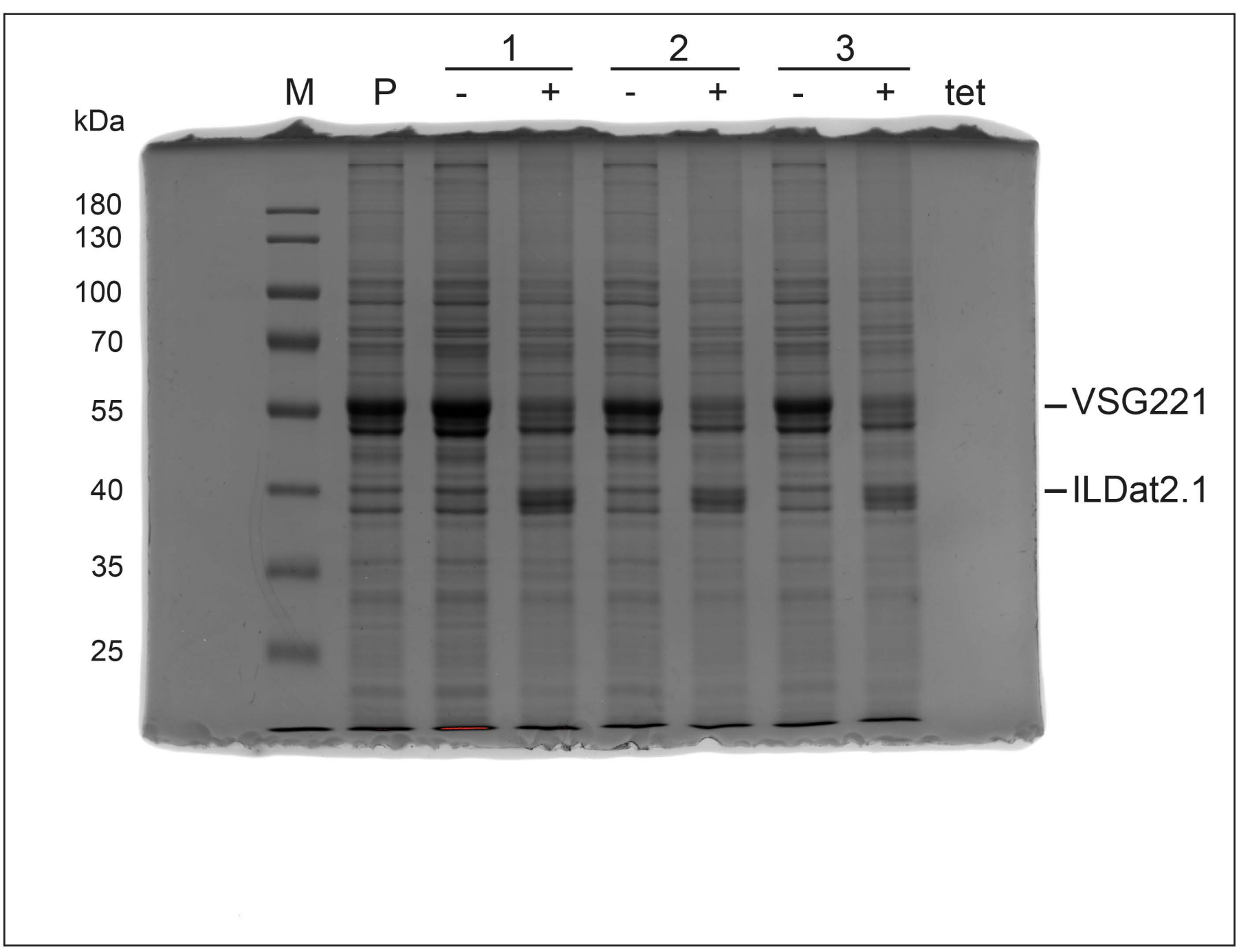

Figure 3F

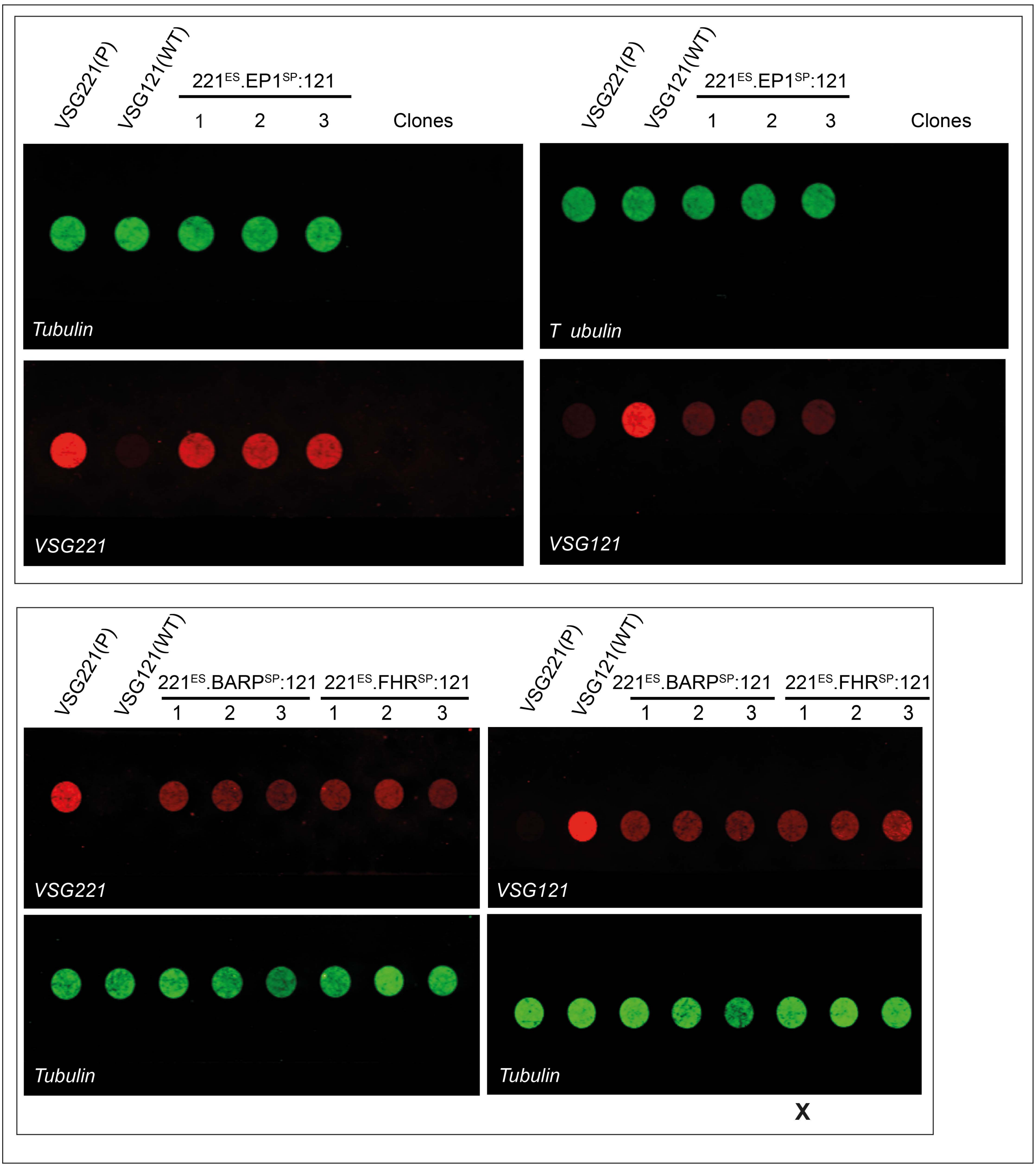

Figure 5C

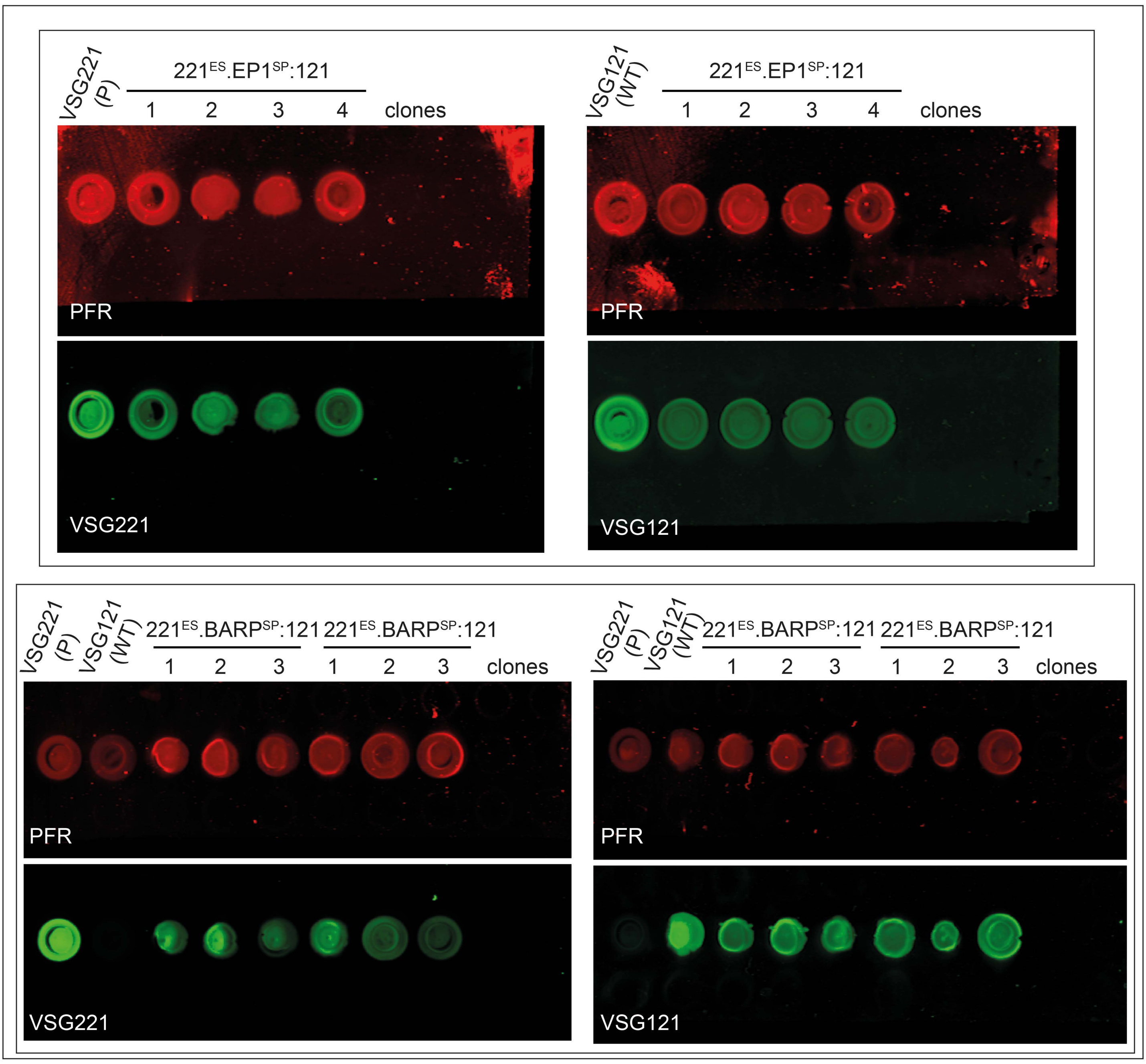

Figure 5D

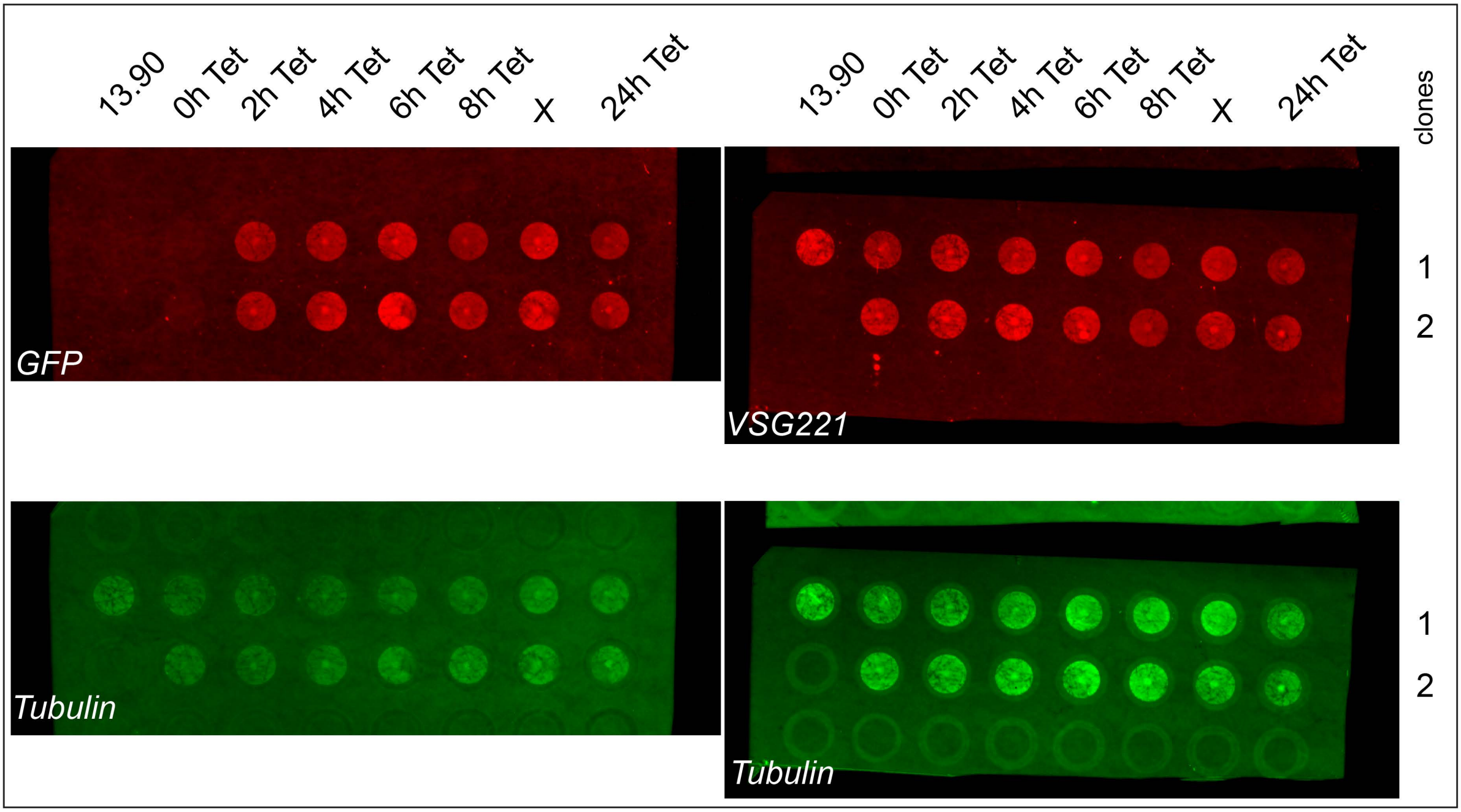

Figure 6E

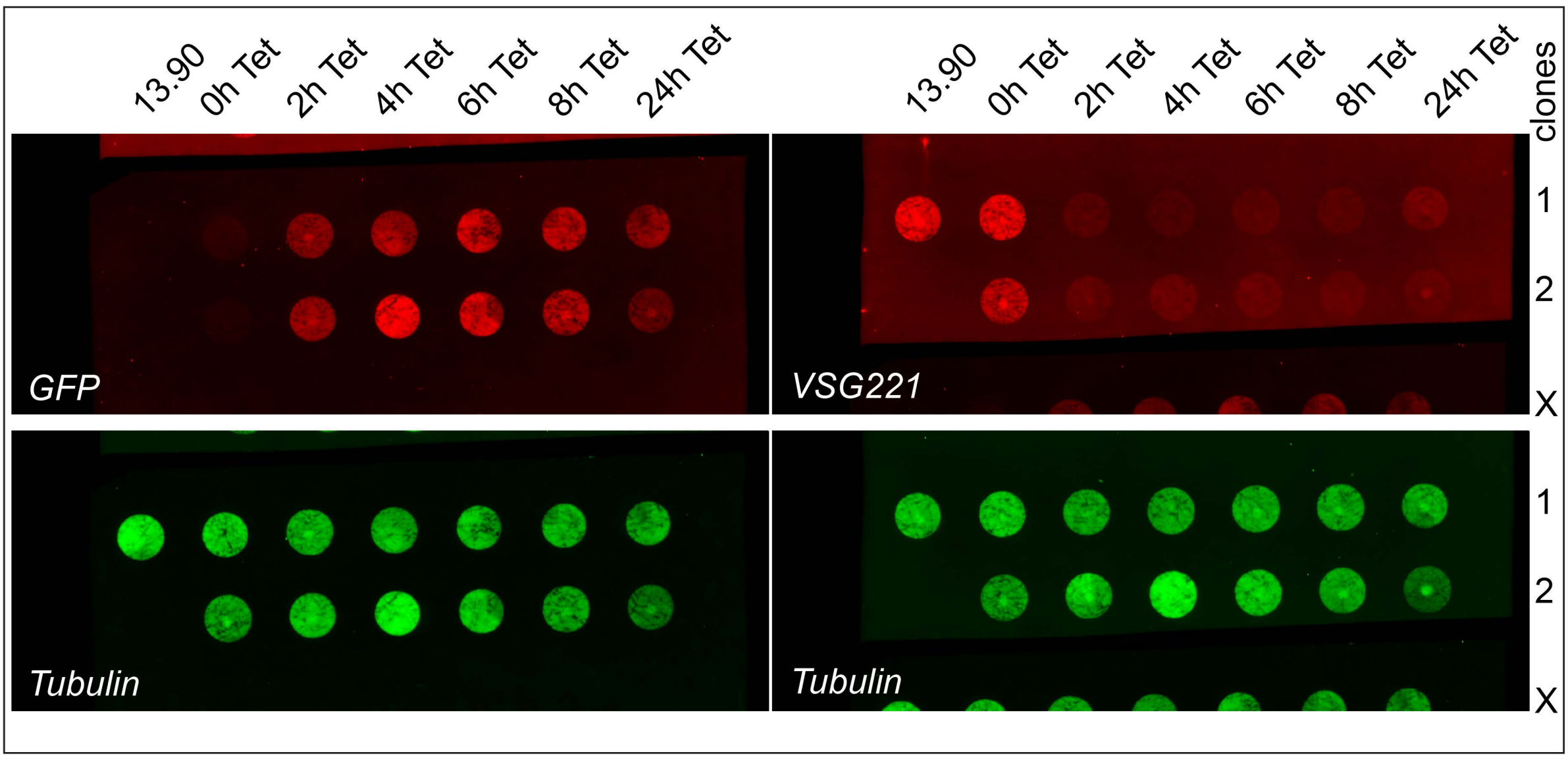

Figure 6F

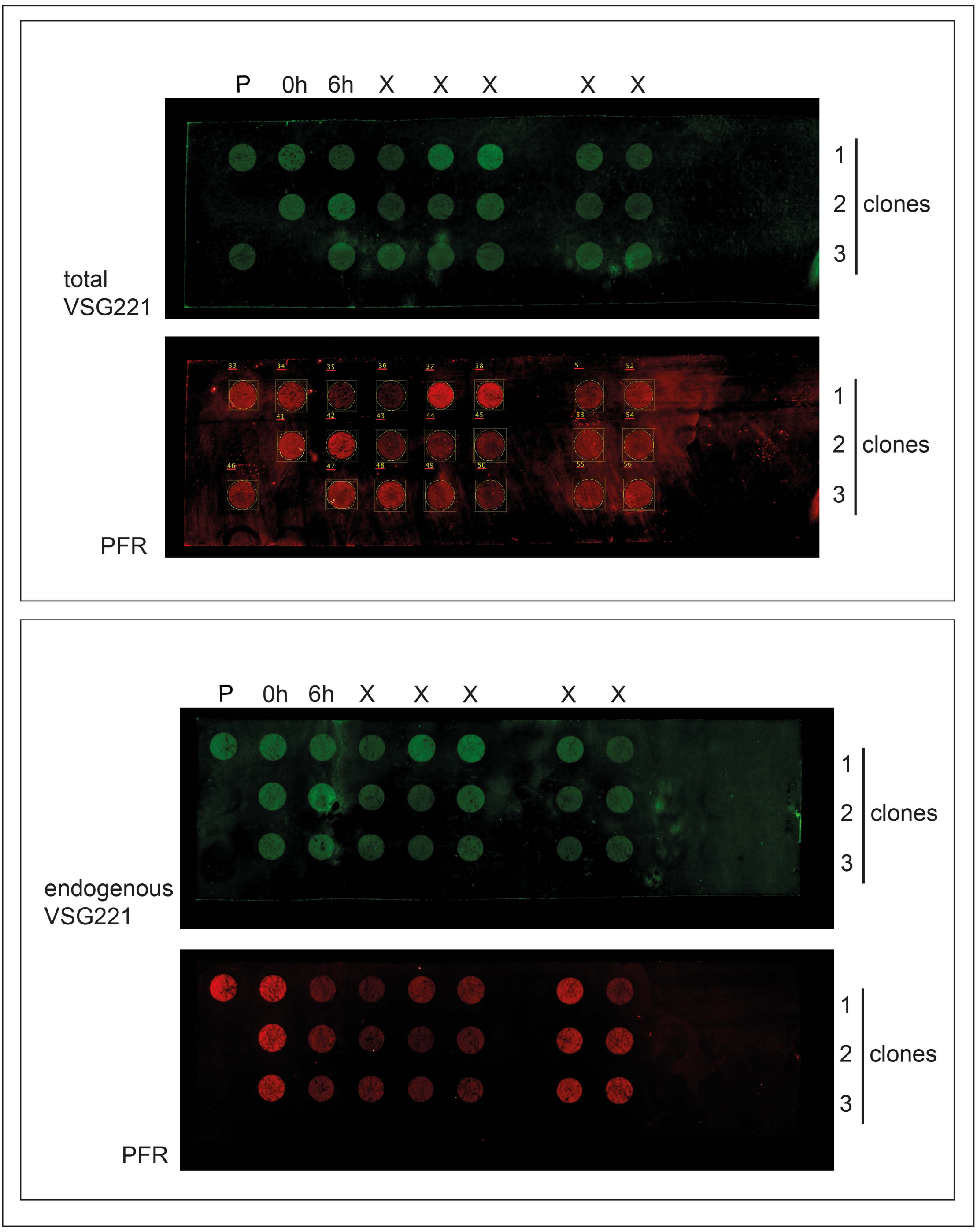

Figure 7B

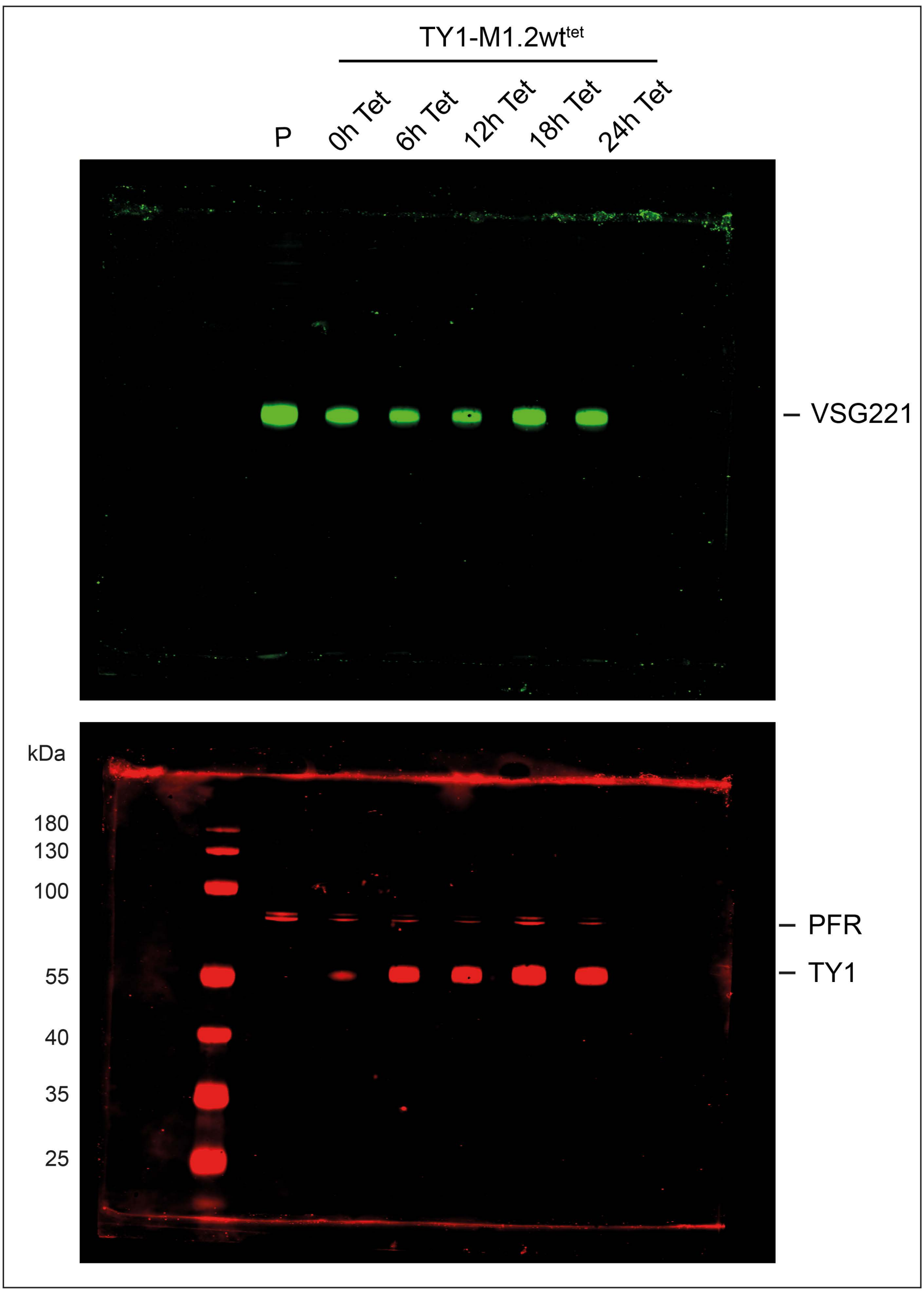

Figure 7C

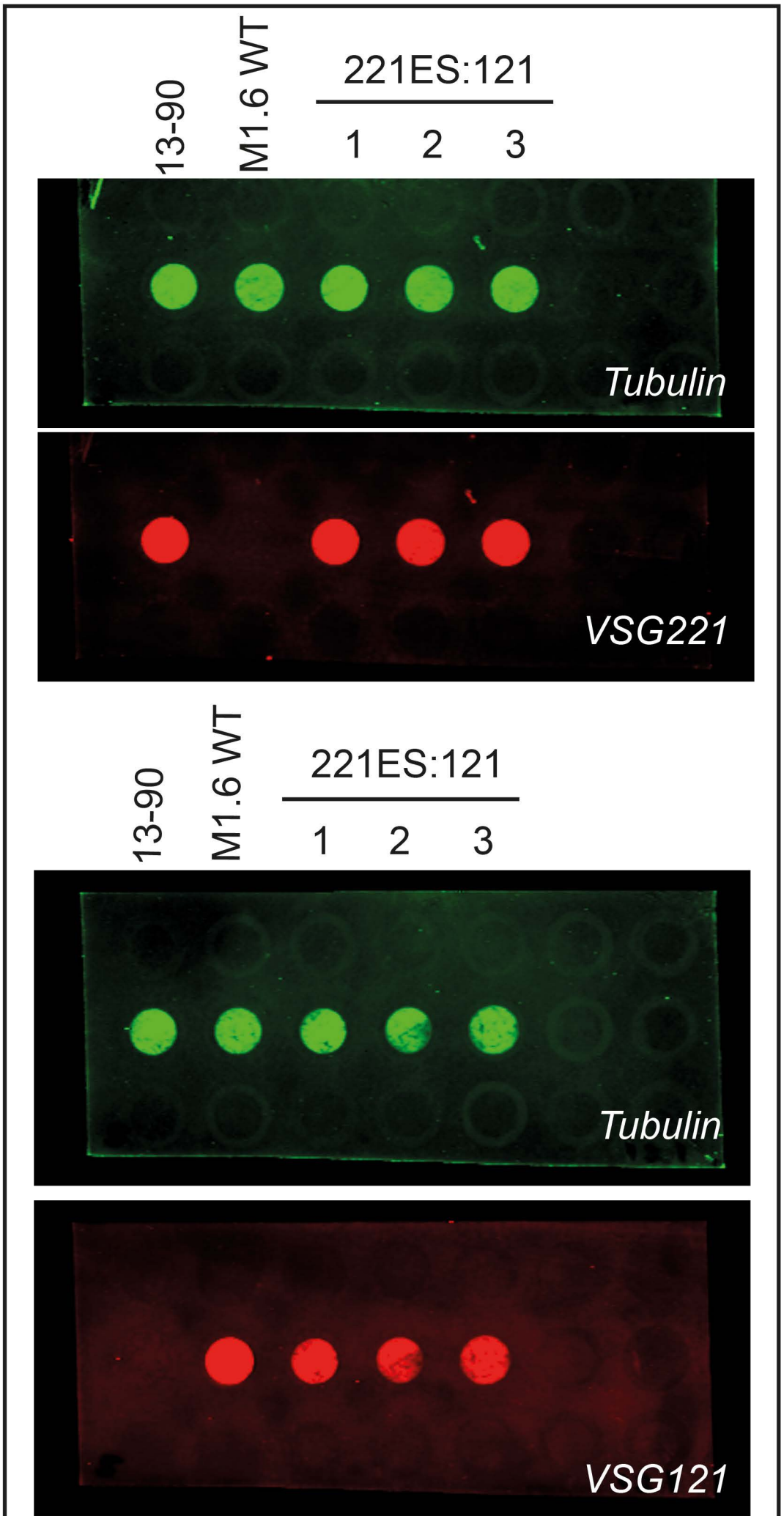

S4A Figure

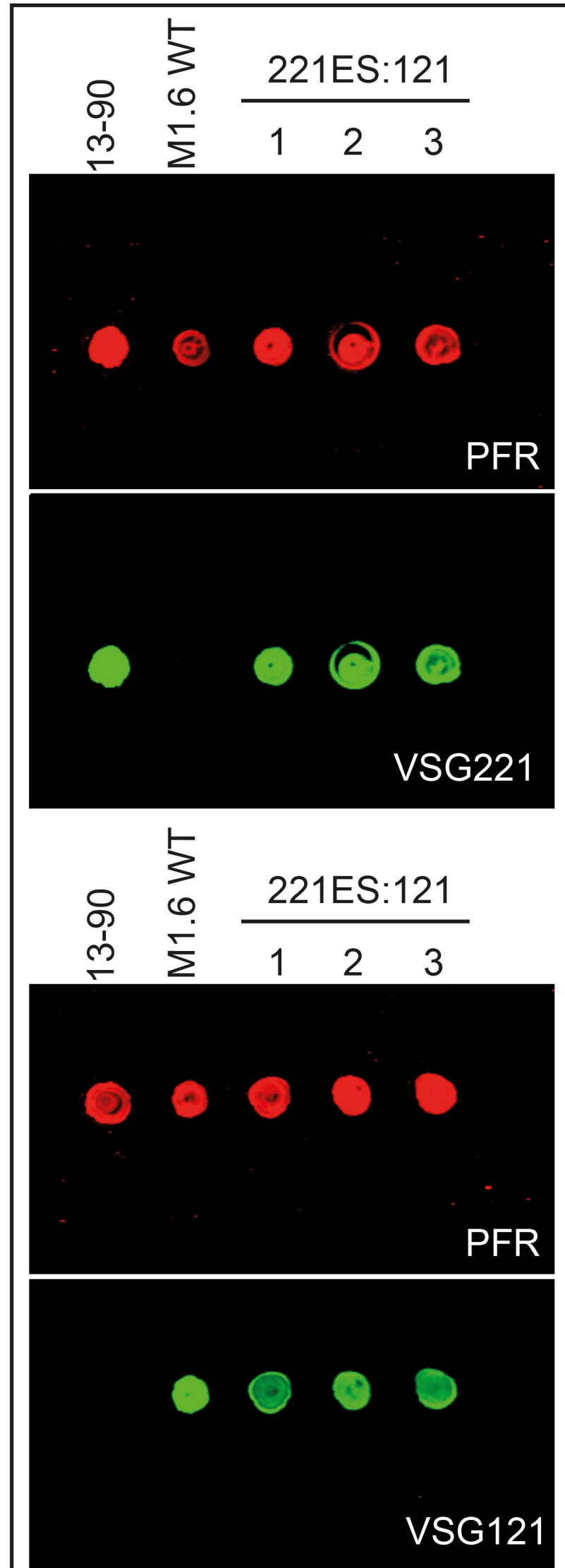

S4B Figure

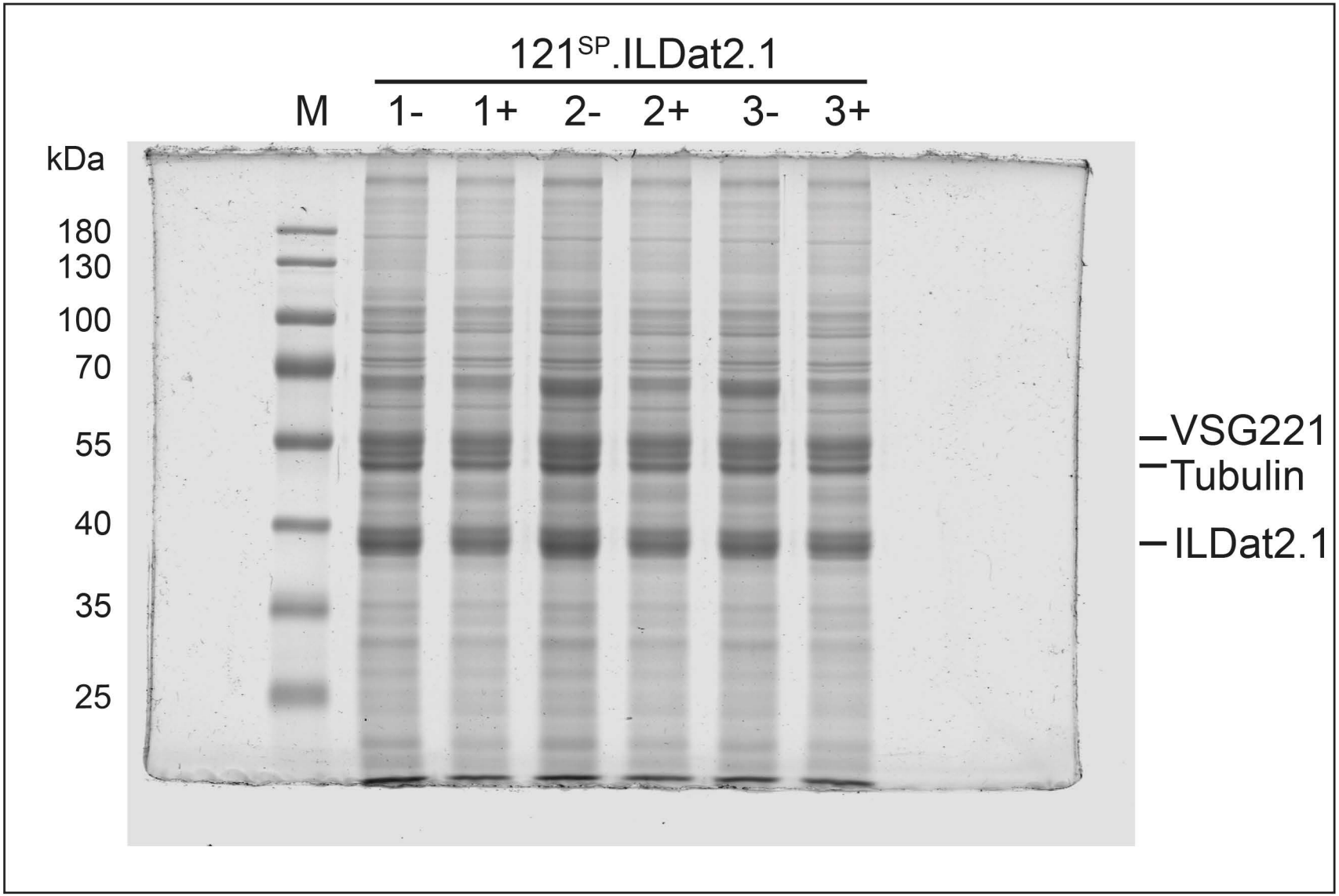
121SP.ILDat2.1
M
1-
1+
2-
2+
3-
3+
kDa
180
130
100
70
55
40
35
25
VSG221
Tubulin
ILDat2.1

S5 Figure

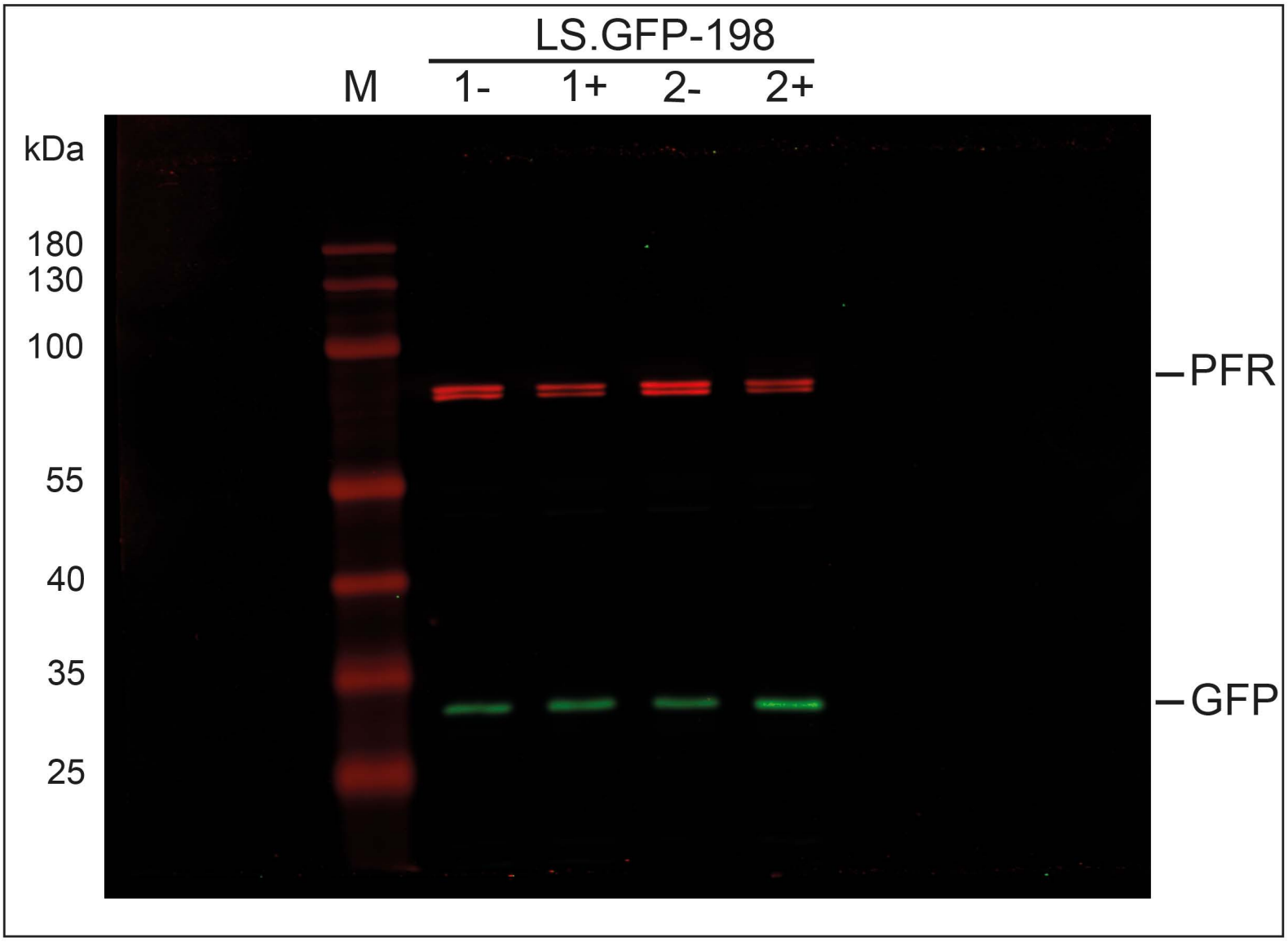

S6A Figure

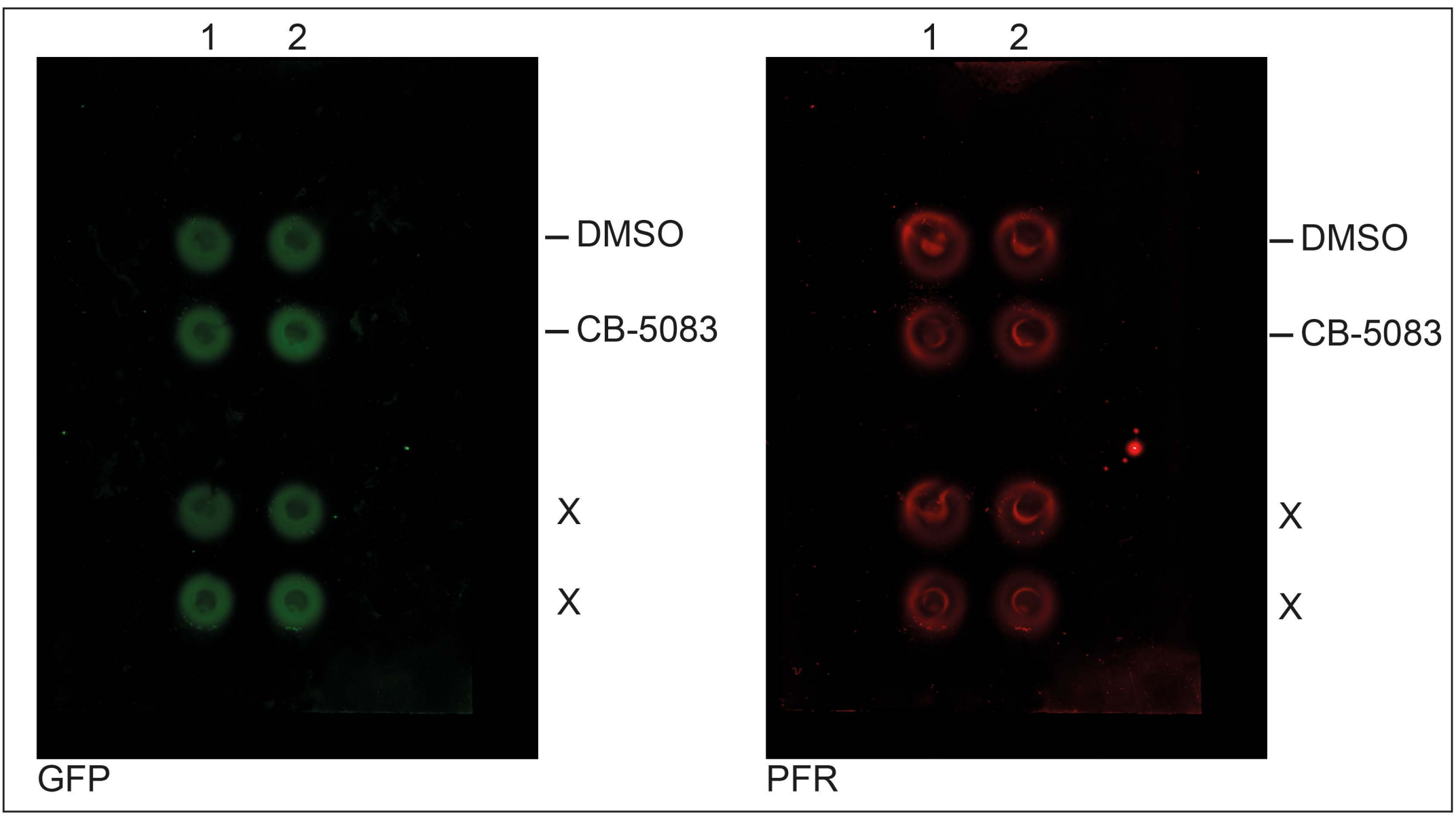

S6B Figure
