# Supplementary material for: Endoplasmic reticulum engagement of transcripts triggers VSG mRNA balancing in trypanosomes
Source: PLoS Pathog. 2026 Aug 18;22(8):e1014518. doi: 10.1371/journal.ppat.1014518 (PMC13515020; doi:10.1371/journal.ppat.1014518)
Supplement: Supplementary file 2 [file ppat.1014518.s009.docx]

***ILDat2.1* ORF**

ATGGATTGGGGGGGTGACAATGCGGAAAATATTGGCGCTTGCAGCGTGTGGCTGCTCTTCGCGAGGCACGCGGAGTGCGCGGAAGGCGGCAACGCAGCGGAGTTTCAGCTTCTCTGCGACGCCGCGCGACTGGCAGCAGCAATGGCAACTGGCAAGGAGCGGTCCATGCTGGCGACAACGGGGTCGCCAGAAAGCGTCGCGAGAGTGGAGGTGGGTCAAGGGTTGATTGGAGCGGCACTGGAGGCGTGGCTGAATGGGCAAAGTGACGAGGCTGATAAAAAAATCGCGGAGGAAACTGCAAGCAGGATGACGGAGGCGAGCCAGACGCACAGCGACGCATTGAAGGCCGCACTGACGGCAGCCGAGGCACACGCGCGAAGTCAAGCACAGAGCAAACAGGCAGAGGCCAAGAAACTCGCCGCACAGGCACTCGGCGTGGATGAGGGGACACTGACAATCACACAGAGCTCGACAAAGTGCGGCGAGCAGAAGTGGACAAGCAGCGCGCCAGCAAGTTACGCCACCACCCACGCGGGGAAATGCATCGCCGGCGACATCATGTACCTGTGCAAAACGCAAGCACAGAACGAATGCCTCAAAGGCCTGGTCTCAAGCAACGACATCGCCCAGAACAACGCCGTCGAAAAGTGGGAAACAGACATCAAGACACGCATCGACAAGCCATGCCTCGCCGGCGATCACACAGGCATCGAAGTAACACTCGACGCAGTGGACACAGCTATAGGGCGCATTCGCGCTGCCGTCGGCACAAAGTCAGGGAAGGACAAGTTCGGCAGTGACAGCAGCACAACGCTCACGTACACAGCAGCGTTCGCAACAAACGGCAACGGCAACGAGGGCGAACACTTCTGGGGGAAAAAACTGAAGCAGGCCGCAACGCACCTGGCCCAAGCACGCCAAGCGCTGGCATTCGCACAAGAAATGCAAGCACGCAAAAGAAAAATCGAGAACACAGAAAGGCTCGTCGGCGACCCACTGGCCGAAACAAAAACGCGACGCGCAGCCAAGGAACAAACAGCGACAGAAGCACAAAAACGGGACCATCAAGAGGGTAACGAGGACATGAGCACACGCGAGACAGGGAAGAAGGACACATCAACACGCGTGGCACCGCACCTGCTTTTGGTGGCCACCATGCTAGCTACAGCAACGGGCTGA

***ILDat1.2* ORF**

ATGTGGCAGCGAATTTTGGTGAGTGTCGGTGTGATCGCGCTGGTCGCCGGAAAGGCAAGGGGCGCTAACAACTTCGCCGAGGCCGACATGAAGGGTGTCTGCACCGGCGCGAAGACACTGCGCGAAGCGGGGAGCGCGGCGAGCTACGTGGTGGAGGAAATGCAAAAGGAGGGCGAGAGGCTGACGGCAATTCTCGGCCAGCTGTGGCTTGTGACCGGCGCGGCGCAAGCCGCCAACAACGACACGGTTGTGCAGGCGGCAACGAAGGAAGCTCAGCAGGTGGGGGAAAAGCTaGCGGCGGTACGCGACACCGCGAACAAGGCTTCGGCACTCAGCACCGCCGCAGCTACGATTTCGGCGCGCCTGTACGAgTTCGTGGCTATGGCAAGCGCCATGGCAGGGAAGTCAGGCAGCTCGGGATACAAGTGCATCGGCGGAAACTCGGCGGAGCAAACAGGCGGCGTCACGCAAGACGCACAAATACCGGCTGATTGCAAGGCACAGGTGACTAAGCCGACAGACCTCGCGACCGCCAAGCAGAAAATTACTGCAGCTAAAACTGTCAAACTGACACCGAGCATCGGAGCTTTCACCACAAACGCATTCAACGGCGCTCAGAGTAGTGGCGCAAAGTGCCCGCTCACAGTCAAGGGCACAAGCTCCGAAGCAGGCCTCGTACAGTCAGGCAAGGTCATTTTCGCAGGCGTCATAGAAGCAGACCCATCTGGCGCAACCGCAATGGCAGTTAAGGAGGACACCACTCTGAAAGCACTCGACACAGACACAAGCCTGCAAAAAATTAAAGCAGACCTTCAGGCGCTCGAATACGCGGTGCAACAAGTGACGATGGAAAAGGGCTCGGTGTGCACGCCACTCACTGGCAACGCAATTGAGTGCAGCAACGCCAAAATGTTTGCGGAGCAAGCACGGCAACTCGCTCAGGAGACAACAAACACACATCGGGAAAATGAACGGCAACGAATGGAAATAGCGCAATCGCAAAGAGGTACCGCACGAAAACAGGGCACGACGCACAAGTCGGAGGCAGAGAACACAGGGGCCTCGACACAGCAGCATGCAGACACCGACCCAACAAGCGCGGACACGCAAGTGCAAGACCGCAAAGCGCGCAACGGCCTCAACACGCCACACACAGTGACGACGCGGGCAGCAACGGCTTTGCTGCTGGTGCGCGCGGTAGCCGCGCAGCAGTGA

***ILDat1.2* ORF with the coding region for the GPI signal sequence replaced by that *of T. brucei* *VSG MITat1.11***

ATGTGGCAGCGAATTTTGGTGAGTGTCGGTGTGATCGCGCTGGTCGCCGGAAAGGCAAGGGGCGCTAACAACTTCGCCGAGACCGACATGCAGGGTGTCTGCACCGGCGCGAAGACACTGCGCGAAGCGGGGAGCGCGGCGAGCTACGTGGTGGAGGAAATGCAAAAGGAGGGCGAGAGGCTGACGGCAATTCTCGGCCAGCTGTGGCTTGTGACCGGCGCGGCGCAAGCCGCCAACAACGACACGGTTGTGCAGGCGGCAACGAAGGAAGCTCAGCAGGTGGGGGAAAAGCTaGCGGCGGTACGCGACACCGCGAACAAGGCTTCGGCACTCAGCACCGCCGCAGCTACGATTTCGGCGCGCCTGTACGAgTTCGTGGCTATGGCAAGCGCCATGGCAGGGAAGTCAGGCAGCTCGGGATACAAGTGCATCGGCGGAAACTCGGCGGAGCAAACAGGCGGCGTCACGCAAGACGCACAAATACCGGCTGATTGCAAGGCACAGGTGACTAAGCCGACAGACCTCGCGACCGCCAAGCAGAAAATTACTGCAGCTAAAACTGTCAAACTGACACCGAGCATCGGAGCTTTCACCACAAACGCATTCAACGGCGCTCAGAGTAGTGGCGCAAAGTGCCCGCTCACAGTCAAGGGCACAAGCTCCGAAGCAGGCCTCGTACAGTCAGGCAAGGTCATTTTCGCAGGCGTCATAGAAGCAGACCCATCTGGCGCAACCGCAATGGCAGTTAAGGAGGACACCACTCTGAAAGCACTCGACACAGACACAAGCCTGCAAAAAATTAAAGCAGACCTTCAGGCGCTCGAATACGCGGTGCAACAAGTGACGATGGAAAAGGGCTCGGTGTGCACGCCACTCACTGGCAACGCAATTGAGTGCAGCAACGCCAAAATGTTTGCGGAGCAAGCACGGCAACTCGCTCAGGAGACAACAAACACACATCGGGAAAATGAACGGCAACGAATGGAAATAGCGCAATCGCAAAGAGGTACCGCACGAAAACAGGGCACGACGCACAAGTCGGAGGCAGAGAACACAGGGGCCTCGACACAGCAGCATGCAGACACCGACCCAACAAGCGCGGACACGCAAGTGCAAGACCGCAAAGCGCGCGATTCCAGTTTTCTAGTAAGCAAACAATTCGCCCTAATGGTTTCTTCTGCATTTGCGGCCTTACTTTTTTAA

***ILDat2.1* ORF with the coding region for the GPI signal sequence replaced by that *of T. brucei* *VSG MITat1.11***

ATGGATTGGGGGGGTGACAATGCGGAAAATATTGGCGCTTGCAGCGTGTGGCTGCTCTTCGCGAGGCACGCGGAGTGCGCGGAAGGCGGCAACGCAGCGGAGTTTCAGCTTCTCTGCGACGCCGCGCGACTGGCAGCAGCAATGGCAACTGGCAAGGAGCGGTCCATGCTGGCGACAACGGGGTCGCCAGAAAGCGTCGCGAGAGTGGAGGTGGGTCAAGGGTTGATTGGAGCGGCACTGGAGGCGTGGCTGAATGGGCAAAGTGACGAGGCTGATAAAAAAATCGCGGAGGAAACTGCAAGCAGGATGACGGAGGCGAGCCAGACGCACAGCGACGCATTGAAGGCCGCACTGACGGCAGCCGAGGCACACGCGCGAAGTCAAGCACAGAGCAAACAGGCAGAGGCCAAGAAACTCGCCGCACAGGCACTCGGCGTGGATGAGGGGACACTGACAATCACACAGAGCTCGACAAAGTGCGGCGAGCAGAAGTGGACAAGCAGCGCGCCAGCAAGTTACGCCACCACCCACGCGGGGAAATGCATCGCCGGCGACATCATGTACCTGTGCAAAACGCAAGCACAGAACGAATGCCTCAAAGGCCTGGTCTCAAGCAACGACATCGCCCAGAACAACGCCGTCGAAAAGTGGGAAACAGACATCAAGACACGCATCGACAAGCCATGCCTCGCCGGCGATCACACAGGCATCGAAGTAACACTCGACGCAGTGGACACAGCTATAGGGCGCATTCGCGCTGCCGTCGGCACAAAGTCAGGGAAGGACAAGTTCGGCAGTGACAGCAGCACAACGCTCACGTACACAGCAGCGTTCGCAACAAACGGCAACGGCAACGAGGGCGAACACTTCTGGGGGAAAAAACTGAAGCAGGCCGCAACGCACCTGGCCCAAGCACGCCAAGCGCTGGCATTCGCACAAGAAATGCAAGCACGCAAAAGAAAAATCGAGAACACAGAAAGGCTCGTCGGCGACCCACTGGCCGAAACAAAAACGCGACGCGCAGCCAAGGAACAAACAGCGACAGAAGCACAAAAACGGGACCATCAAGAGGGTAACGAGGACATGAGCACACGCGAGACAGGGAAGGATTCCAGTTTTCTAGTAAGCAAACAATTCGCCCTAATGGTTTCTTCTGCATTTGCGGCCTTACTTTTTTAA

***ILDat2.1* ORF with the coding region for the ER import signal replaced by that of *T. brucei* *VSG VSG121***

ATGGCCGTGCACAGAGCCCTAGCGGCGTACGCGATTAGTCTTTACGTTTTACTACCCAGAAAATCGGGAGCAGCGGAAGGCGGCAACGCAGCGGAGTTTCAGCTTCTCTGCGACGCCGCGCGACTGGCAGCAGCAATGGCAACTGGCAAGGAGCGGTCCATGCTGGCGACAACGGGGTCGCCAGAAAGCGTCGCGAGAGTGGAGGTGGGTCAAGGGTTGATTGGAGCGGCACTGGAGGCGTGGCTGAATGGGCAAAGTGACGAGGCTGATAAAAAAATCGCGGAGGAAACTGCAAGCAGGATGACGGAGGCGAGCCAGACGCACAGCGACGCATTGAAGGCCGCACTGACGGCAGCCGAGGCACACGCGCGAAGTCAAGCACAGAGCAAACAGGCAGAGGCCAAGAAACTCGCCGCACAGGCACTCGGCGTGGATGAGGGGACACTGACAATCACACAGAGCTCGACAAAGTGCGGCGAGCAGAAGTGGACAAGCAGCGCGCCAGCAAGTTACGCCACCACCCACGCGGGGAAATGCATCGCCGGCGACATCATGTACCTGTGCAAAACGCAAGCACAGAACGAATGCCTCAAAGGCCTGGTCTCAAGCAACGACATCGCCCAGAACAACGCCGTCGAAAAGTGGGAAACAGACATCAAGACACGCATCGACAAGCCATGCCTCGCCGGCGATCACACAGGCATCGAAGTAACACTCGACGCAGTGGACACAGCTATAGGGCGCATTCGCGCTGCCGTCGGCACAAAGTCAGGGAAGGACAAGTTCGGCAGTGACAGCAGCACAACGCTCACGTACACAGCAGCGTTCGCAACAAACGGCAACGGCAACGAGGGCGAACACTTCTGGGGGAAAAAACTGAAGCAGGCCGCAACGCACCTGGCCCAAGCACGCCAAGCGCTGGCATTCGCACAAGAAATGCAAGCACGCAAAAGAAAAATCGAGAACACAGAAAGGCTCGTCGGCGACCCACTGGCCGAAACAAAAACGCGACGCGCAGCCAAGGAACAAACAGCGACAGAAGCACAAAAACGGGACCATCAAGAGGGTAACGAGGACATGAGCACACGCGAGACAGGGAAGAAGGACACATCAACACGCGTGGCACCGCACCTGCTTTTGGTGGCCACCATGCTAGCTACAGCAACGGGCTGA

***ILDat2.1* ORF with the coding regions for the ER import and GPI signal sequences replaced by those *of T. brucei* *VSG121* and *MITat1.11*, respectively**

ATGGCCGTGCACAGAGCCCTAGCGGCGTACGCGATTAGTCTTTACGTTTTACTACCCAGAAAATCGGGAGCAGCGGAAGGCGGCAACGCAGCGGAGTTTCAGCTTCTCTGCGACGCCGCGCGACTGGCAGCAGCAATGGCAACTGGCAAGGAGCGGTCCATGCTGGCGACAACGGGGTCGCCAGAAAGCGTCGCGAGAGTGGAGGTGGGTCAAGGGTTGATTGGAGCGGCACTGGAGGCGTGGCTGAATGGGCAAAGTGACGAGGCTGATAAAAAAATCGCGGAGGAAACTGCAAGCAGGATGACGGAGGCGAGCCAGACGCACAGCGACGCATTGAAGGCCGCACTGACGGCAGCCGAGGCACACGCGCGAAGTCAAGCACAGAGCAAACAGGCAGAGGCCAAGAAACTCGCCGCACAGGCACTCGGCGTGGATGAGGGGACACTGACAATCACACAGAGCTCGACAAAGTGCGGCGAGCAGAAGTGGACAAGCAGCGCGCCAGCAAGTTACGCCACCACCCACGCGGGGAAATGCATCGCCGGCGACATCATGTACCTGTGCAAAACGCAAGCACAGAACGAATGCCTCAAAGGCCTGGTCTCAAGCAACGACATCGCCCAGAACAACGCCGTCGAAAAGTGGGAAACAGACATCAAGACACGCATCGACAAGCCATGCCTCGCCGGCGATCACACAGGCATCGAAGTAACACTCGACGCAGTGGACACAGCTATAGGGCGCATTCGCGCTGCCGTCGGCACAAAGTCAGGGAAGGACAAGTTCGGCAGTGACAGCAGCACAACGCTCACGTACACAGCAGCGTTCGCAACAAACGGCAACGGCAACGAGGGCGAACACTTCTGGGGGAAAAAACTGAAGCAGGCCGCAACGCACCTGGCCCAAGCACGCCAAGCGCTGGCATTCGCACAAGAAATGCAAGCACGCAAAAGAAAAATCGAGAACACAGAAAGGCTCGTCGGCGACCCACTGGCCGAAACAAAAACGCGACGCGCAGCCAAGGAACAAACAGCGACAGAAGCACAAAAACGGGACCATCAAGAGGGTAACGAGGACATGAGCACACGCGAGACAGGGAAGGATTCCAGTTTTCTAGTAAGCAAACAATTCGCCCTAATGGTTTCTTCTGCATTTGCGGCCTTACTTTTTTAA

***VSG121* ORF with the coding region for the ER import signal replaced by that of *T. congolense* *VSG VSG221***

ATGGATTGGGGGGGTGACAATGCGGAAAATATTGGCGCTTGCAGCGTGTGGCTGCTCTTCGCGAGGCACGCGGAGTGCACAGACAAAGGCGCGATCAAGTTTGAGACGTGGGAGCCGCTCTGTTTACTGACACAAGACTTCGGTAACCTTTACAACAGAGCGCACAAACTTAATCTCGACATCGACACCTACGTAACCGCAGCCCAGGCCGACCAACTGCGGCTCCAGGTTCTACTAAGTCGCGCTAGCAGCAAGATAGAAGCTGCTGCAGCCGCGGCAGCAACAGCAGCAATAGCAGCCGATCTAGCCGGCAAAGCTAAGCACGTCGCCAGCTGCAAGCTGGCAGCAACAACACTGACAGCCACAACAGGGTACTTGCACGGCCGCATTGCTGAATTTCTAGAGGTCATGACAGCTCACAGGGGAACAACAAACAAATACGGCTGCCTTTCAAAAAGCAGATCAGACAACTCAAATTCGATAACGGACAGCGTCGCAAATCTAAAAGACAAGTGCAAGCTGACAGTGCAACAAATAAGTCCGGACAACAAAGCAACCGAGCAGATAACCAAAGCAGGCTTCACGAAACTAACGGCGACCGGCGGGCTAACAAGCAGCGACCTGGGAGGCAGCGGCCAAGCAGTCTGCATGATTCTGAGCACCACAGCCAGCGAAGTGGTAAACAACGGAAACCTAGATGAACCGGTCCCTTACGCCGGCGGCTACCTAAGACGTAAACACGACCTGACATCGGACGGTAACGACAACTTAGCGACCATCACAGACTCAGCTTCAGCGCCAGCAACCCGGGCCAAGACAGACCCCTATCTCCAGATCTGGCGGGCCTTCAAAAACCTAGAAGACTGCGAATCCACGTTCACAAGCGGCTACAGCCGACCATCGCCAGAAACATTAAAAGCAGCTGACGAAACAAAAACAGCAATTAAAAACTATGTAGTACAGAAGGAAGGCAAATACGACCAGGCTACAGACAAAGAAGACGACTACAAGAACCTCGACAAGATATTCAAGGATGGCAAGGACTTTTACCCGCAAAAACTCTGGGATGCCATGGATAAGAAAGATCTGCTAAAAGATGCGACGCAAACCAACGAGATCAAAAAACTTGCCGACATAACCGACAGAAGCGAACTAAACAAGGTTCTGTTATATTACACAAGACAGAAGGAACAAACCTTAACTAAAGAACTAAAAGAAGCACAAGAAAAAGCAACACAGGCGAACCAGAATGACGCTGCAGCCAAAGCAGCAGAAGACTCTTGTAATAAGCTAGTTGGCGGAGAAAAATGCAACGCTGATAAAAAATGCAGCTATGAGACAGAAACTGATGGCACCAAAAAGTGCAAATTTAATGCTACCAAAGCTGAAAAAAGTGGTGCCCCTGTAACACAAGCTCAAACGGTAGGAGAAACCGAAGCAACGCCAGAAAAGTGCAAAGGCAAAGACGCCAAGACATGCGGCACCACTCAGGGTTGCAAATGGGAGGGTGAAACTTGCAAAGATTCCTCTATTCTAGTAACCAAGAAATTCGCCCTCACCGTGGTTTCTGCTGCATTTGTGGCCTTGCTTTTTTAA

***VSG121* ORF with the coding region for the ER import signal replaced by that of *T. brucei BARP***

ATGAGCATCACTTTTCATAGTTTATGGCTACTTTTGACAGTGTTATGCACTGCAGGTATTCGTGCTACAGACAAAGGCGCGATCAAGTTTGAGACGTGGGAGCCGCTCTGTTTACTGACACAAGACTTCGGTAACCTTTACAACAGAGCGCACAAACTTAATCTCGACATCGACACCTACGTAACCGCAGCCCAGGCCGACCAACTGCGGCTCCAGGTTCTACTAAGTCGCGCTAGCAGCAAGATAGAAGCTGCTGCAGCCGCGGCAGCAACAGCAGCAATAGCAGCCGATCTAGCCGGCAAAGCTAAGCACGTCGCCAGCTGCAAGCTGGCAGCAACAACACTGACAGCCACAACAGGGTACTTGCACGGCCGCATTGCTGAATTTCTAGAGGTCATGACAGCTCACAGGGGAACAACAAACAAATACGGCTGCCTTTCAAAAAGCAGATCAGACAACTCAAATTCGATAACGGACAGCGTCGCAAATCTAAAAGACAAGTGCAAGCTGACAGTGCAACAAATAAGTCCGGACAACAAAGCAACCGAGCAGATAACCAAAGCAGGCTTCACGAAACTAACGGCGACCGGCGGGCTAACAAGCAGCGACCTGGGAGGCAGCGGCCAAGCAGTCTGCATGATTCTGAGCACCACAGCCAGCGAAGTGGTAAACAACGGAAACCTAGATGAACCGGTCCCTTACGCCGGCGGCTACCTAAGACGTAAACACGACCTGACATCGGACGGTAACGACAACTTAGCGACCATCACAGACTCAGCTTCAGCGCCAGCAACCCGGGCCAAGACAGACCCCTATCTCCAGATCTGGCGGGCCTTCAAAAACCTAGAAGACTGCGAATCCACGTTCACAAGCGGCTACAGCCGACCATCGCCAGAAACATTAAAAGCAGCTGACGAAACAAAAACAGCAATTAAAAACTATGTAGTACAGAAGGAAGGCAAATACGACCAGGCTACAGACAAAGAAGACGACTACAAGAACCTCGACAAGATATTCAAGGATGGCAAGGACTTTTACCCGCAAAAACTCTGGGATGCCATGGATAAGAAAGATCTGCTAAAAGATGCGACGCAAACCAACGAGATCAAAAAACTTGCCGACATAACCGACAGAAGCGAACTAAACAAGGTTCTGTTATATTACACAAGACAGAAGGAACAAACCTTAACTAAAGAACTAAAAGAAGCACAAGAAAAAGCAACACAGGCGAACCAGAATGACGCTGCAGCCAAAGCAGCAGAAGACTCTTGTAATAAGCTAGTTGGCGGAGAAAAATGCAACGCTGATAAAAAATGCAGCTATGAGACAGAAACTGATGGCACCAAAAAGTGCAAATTTAATGCTACCAAAGCTGAAAAAAGTGGTGCCCCTGTAACACAAGCTCAAACGGTAGGAGAAACCGAAGCAACGCCAGAAAAGTGCAAAGGCAAAGACGCCAAGACATGCGGCACCACTCAGGGTTGCAAATGGGAGGGTGAAACTTGCAAAGATTCCTCTATTCTAGTAACCAAGAAATTCGCCCTCACCGTGGTTTCTGCTGCATTTGTGGCCTTGCTTTTTTAA

***VSG121* ORF with the coding region for the ER import signal replaced by that of *T. brucei EP1***

ATGGCACCTCGTTCCCTTTATCTGCTCGCTGTTCTTCTGTTCAGCGCGAACCTCTTCGCTGGCGTGGGATTTGCCGCAGCCACAGACAAAGGCGCGATCAAGTTTGAGACGTGGGAGCCGCTCTGTTTACTGACACAAGACTTCGGTAACCTTTACAACAGAGCGCACAAACTTAATCTCGACATCGACACCTACGTAACCGCAGCCCAGGCCGACCAACTGCGGCTCCAGGTTCTACTAAGTCGCGCTAGCAGCAAGATAGAAGCTGCTGCAGCCGCGGCAGCAACAGCAGCAATAGCAGCCGATCTAGCCGGCAAAGCTAAGCACGTCGCCAGCTGCAAGCTGGCAGCAACAACACTGACAGCCACAACAGGGTACTTGCACGGCCGCATTGCTGAATTTCTAGAGGTCATGACAGCTCACAGGGGAACAACAAACAAATACGGCTGCCTTTCAAAAAGCAGATCAGACAACTCAAATTCGATAACGGACAGCGTCGCAAATCTAAAAGACAAGTGCAAGCTGACAGTGCAACAAATAAGTCCGGACAACAAAGCAACCGAGCAGATAACCAAAGCAGGCTTCACGAAACTAACGGCGACCGGCGGGCTAACAAGCAGCGACCTGGGAGGCAGCGGCCAAGCAGTCTGCATGATTCTGAGCACCACAGCCAGCGAAGTGGTAAACAACGGAAACCTAGATGAACCGGTCCCTTACGCCGGCGGCTACCTAAGACGTAAACACGACCTGACATCGGACGGTAACGACAACTTAGCGACCATCACAGACTCAGCTTCAGCGCCAGCAACCCGGGCCAAGACAGACCCCTATCTCCAGATCTGGCGGGCCTTCAAAAACCTAGAAGACTGCGAATCCACGTTCACAAGCGGCTACAGCCGACCATCGCCAGAAACATTAAAAGCAGCTGACGAAACAAAAACAGCAATTAAAAACTATGTAGTACAGAAGGAAGGCAAATACGACCAGGCTACAGACAAAGAAGACGACTACAAGAACCTCGACAAGATATTCAAGGATGGCAAGGACTTTTACCCGCAAAAACTCTGGGATGCCATGGATAAGAAAGATCTGCTAAAAGATGCGACGCAAACCAACGAGATCAAAAAACTTGCCGACATAACCGACAGAAGCGAACTAAACAAGGTTCTGTTATATTACACAAGACAGAAGGAACAAACCTTAACTAAAGAACTAAAAGAAGCACAAGAAAAAGCAACACAGGCGAACCAGAATGACGCTGCAGCCAAAGCAGCAGAAGACTCTTGTAATAAGCTAGTTGGCGGAGAAAAATGCAACGCTGATAAAAAATGCAGCTATGAGACAGAAACTGATGGCACCAAAAAGTGCAAATTTAATGCTACCAAAGCTGAAAAAAGTGGTGCCCCTGTAACACAAGCTCAAACGGTAGGAGAAACCGAAGCAACGCCAGAAAAGTGCAAAGGCAAAGACGCCAAGACATGCGGCACCACTCAGGGTTGCAAATGGGAGGGTGAAACTTGCAAAGATTCCTCTATTCTAGTAACCAAGAAATTCGCCCTCACCGTGGTTTCTGCTGCATTTGTGGCCTTGCTTTTTTAA

***VSG121* ORF with the coding region for the ER import signal replaced by that of *T. brucei FHR***

ATGATGATTTCCCGCGCTTTGTTTGTGCTCGGCCTCAGTCTTCACTTCATATTCGCACCGACCGCCTACGCGACAGACAAAGGCGCGATCAAGTTTGAGACGTGGGAGCCGCTCTGTTTACTGACACAAGACTTCGGTAACCTTTACAACAGAGCGCACAAACTTAATCTCGACATCGACACCTACGTAACCGCAGCCCAGGCCGACCAACTGCGGCTCCAGGTTCTACTAAGTCGCGCTAGCAGCAAGATAGAAGCTGCTGCAGCCGCGGCAGCAACAGCAGCAATAGCAGCCGATCTAGCCGGCAAAGCTAAGCACGTCGCCAGCTGCAAGCTGGCAGCAACAACACTGACAGCCACAACAGGGTACTTGCACGGCCGCATTGCTGAATTTCTAGAGGTCATGACAGCTCACAGGGGAACAACAAACAAATACGGCTGCCTTTCAAAAAGCAGATCAGACAACTCAAATTCGATAACGGACAGCGTCGCAAATCTAAAAGACAAGTGCAAGCTGACAGTGCAACAAATAAGTCCGGACAACAAAGCAACCGAGCAGATAACCAAAGCAGGCTTCACGAAACTAACGGCGACCGGCGGGCTAACAAGCAGCGACCTGGGAGGCAGCGGCCAAGCAGTCTGCATGATTCTGAGCACCACAGCCAGCGAAGTGGTAAACAACGGAAACCTAGATGAACCGGTCCCTTACGCCGGCGGCTACCTAAGACGTAAACACGACCTGACATCGGACGGTAACGACAACTTAGCGACCATCACAGACTCAGCTTCAGCGCCAGCAACCCGGGCCAAGACAGACCCCTATCTCCAGATCTGGCGGGCCTTCAAAAACCTAGAAGACTGCGAATCCACGTTCACAAGCGGCTACAGCCGACCATCGCCAGAAACATTAAAAGCAGCTGACGAAACAAAAACAGCAATTAAAAACTATGTAGTACAGAAGGAAGGCAAATACGACCAGGCTACAGACAAAGAAGACGACTACAAGAACCTCGACAAGATATTCAAGGATGGCAAGGACTTTTACCCGCAAAAACTCTGGGATGCCATGGATAAGAAAGATCTGCTAAAAGATGCGACGCAAACCAACGAGATCAAAAAACTTGCCGACATAACCGACAGAAGCGAACTAAACAAGGTTCTGTTATATTACACAAGACAGAAGGAACAAACCTTAACTAAAGAACTAAAAGAAGCACAAGAAAAAGCAACACAGGCGAACCAGAATGACGCTGCAGCCAAAGCAGCAGAAGACTCTTGTAATAAGCTAGTTGGCGGAGAAAAATGCAACGCTGATAAAAAATGCAGCTATGAGACAGAAACTGATGGCACCAAAAAGTGCAAATTTAATGCTACCAAAGCTGAAAAAAGTGGTGCCCCTGTAACACAAGCTCAAACGGTAGGAGAAACCGAAGCAACGCCAGAAAAGTGCAAAGGCAAAGACGCCAAGACATGCGGCACCACTCAGGGTTGCAAATGGGAGGGTGAAACTTGCAAAGATTCCTCTATTCTAGTAACCAAGAAATTCGCCCTCACCGTGGTTTCTGCTGCATTTGTGGCCTTGCTTTTTTAA

***GFP* ORF**

ATGGTGAGCAAGGGCGAGGAGCTGTTCACCGGGGTGGTGCCCATCCTGGTCGAGCTGGACGGCGACGTAAACGGCCACAAGTTCAGCGTGTCCGGCGAGGGCGAGGGCGATGCCACCTACGGCAAGCTGACCCTGAAGTTCATCTGCACCACCGGCAAGCTGCCCGTGCCCTGGCCCACCCTCGTGACCACCCTGACCTACGGCGTGCAGTGCTTCAGCCGCTACCCCGACCACATGAAGCAGCACGACTTCTTCAAGTCCGCCATGCCCGAAGGCTACGTCCAGGAGCGCACCATCTTCTTCAAGGACGACGGCAACTACAAGACCCGCGCCGAGGTGAAGTTCGAGGGcGACACCCTGGTGAACCGCATCGAGCTGAAGGGCATCGACTTCAAGGAGGACGGCAACATCCTGGGGCACAAGCTGGAGTACAACTACAACAGCCACAACGTCTATATCATGGCCGACAAGCAGAAGAACGGCATCAAGGTGAACTTCAAGATCCGCCACAACATCGAGGACGGCAGCGTGCAGCTCGCCGACCACTACCAGCAGAACACCCCCATCGGcGACGGCCCCGTGCTGCTGCCCGACAACCACTACCTGAGCACCCAGTCCGCCCTGAGCAAAGACCCCAACGAGAAGCGCGATCACATGGTCCTGCTGGAGTTCGTGACCGCCGCCGGGATCACTCTCGGCATGGACGAGCTGTACAAGTAA

***GFP* ORF with the coding region for the ER import signal from *T. brucei EP1* added**

ATGGCACCTCGTTCCCTTTATCTGCTCGCTGTTCTTCTGTTCAGCGCGAACCTCTTCGCTGGCGTGGGATTTGCCGCAGCCGCTGAAGGACCAGAAGACAAGGGTCTTACTAAGGGAGGCAAAGGCAAAGGCGAGAAGGGAACCAAGGTCGGCGGGATCCACCGGCCGGTCGCCACCATGGTGAGCAAGGGCGAGGAGCTGTTCACCGGGGTGGTGCCCATCCTGGTCGAGCTGGACGGCGACGTAAACGGCCACAAGTTCAGCGTGTCCGGCGAGGGCGAGGGCGATGCCACCTACGGCAAGCTGACCCTGAAGTTCATCTGCACCACCGGCAAGCTGCCCGTGCCCTGGCCCACCCTCGTGACCACCCTGACCTACGGCGTGCAGTGCTTCAGCCGCTACCCCGACCACATGAAGCAGCACGACTTCTTCAAGTCCGCCATGCCCGAAGGCTACGTCCAGGAGCGCACCATCTTCTTCAAGGACGACGGCAACTACAAGACCCGCGCCGAGGTGAAGTTCGAGGGcGACACCCTGGTGAACCGCATCGAGCTGAAGGGCATCGACTTCAAGGAGGACGGCAACATCCTGGGGCACAAGCTGGAGTACAACTACAACAGCCACAACGTCTATATCATGGCCGACAAGCAGAAGAACGGCATCAAGGTGAACTTCAAGATCCGCCACAACATCGAGGACGGCAGCGTGCAGCTCGCCGACCACTACCAGCAGAACACCCCCATCGGcGACGGCCCCGTGCTGCTGCCCGACAACCACTACCTGAGCACCCAGTCCGCCCTGAGCAAAGACCCCAACGAGAAGCGCGATCACATGGTCCTGCTGGAGTTCGTGACCGCCGCCGGGATCACTCTCGGCATGGACGAGCTGTACAAGTAA

***TY1-VSG221* ORF**

ATGCCTTCCAATCAGGAGGCCCGGCTTTTCCTCGCCGTCTTGGTCCTAGCCCAAGTTCTTCCAATTCTTGTCGATTCGGCGGCTGAAAAAGGCGCCCGTGAGGTCCATACTAACCAGGACCCACTTGACAAACAAGCTTTTTGGCAACCTCTTTGCCAGGTCTCCGAGGAGCTAGACGACCAACCGAAGGGTGCGTTGTTTACGCTGCAAGCAGCGGCGAGCAAAATCCAGAAAATGAGGGACGCGGCACTGCGAGCAAGTATATACGCTGAAATAAATCACGGCACCAACAGGGCCAAGGCAGCCGTTATAGTCGCCAACCACTATGCCATGAAAGCTGATAGCGGCCTAGAGGCCCTAAAACAAACGTTAAGCAGCCAAGAGGTAACAGCTACTGCAACAGCGAGCTACCTAAAAGGAAGAATAGACGAATACCTAAATCTCCTTCTACAAACAAAGGAGAGCGGCACCAGCGGCTGCATGATGGACACCAGCGGAACAAACACGGTAACGAAGGCCGGCGGCACCATCGGAGGCGTTCCTTGCAAGCTGCAGTTGTCGCCGATACAGCCGAAGCGACCCGCAGCGACCTACCTAGGTAAAGCGGGCTACGTAGGCCTAACACGACAAGCAGATGCAGCCAACAATTTCCACGATAACGACGCCGAATGCAGGCTAGCCAGTGGGCACAACACCAACGGCCTCGGCAAAAGCGGCCAGCTTTCTGCAGCGGTCACTATGGCGGCCGGCTATGTCACAGTAGCGAACAGCCAAACAGCCGTCACGGTCCAGGCGCTCGATGCATTACAGGAAGCGAGCGGAGCAGCGCACCAACCGTGGATCGACGCCTGGAAGGCCAAGAAAGCGCTAACAGGAGCAGAAACCGCTGAGTTCAGAAACGAAACAGCCGGAATAGCTGGCAAAACAGGCGTTACCAAGCTTGTTGAAGAAGCTTTACTAAAGAAAAAAGACTCAGAGGCCTCAGAAATACAAACAGAATTAAAAAAATACTTTAGCGGCCACGAAAATGAACAGTGGACAGCAATAGAAAAGCTCATATCCGAGCAGCCAGTGGCGCAAAACCTGGTAGGCGACAACCAGCCAACCAAGCTAGGGGAACTGGAGGGCAATGCCAAGTTAACGACTATACTTGCCTATTACCGAATGGAAACAGCAGGGAAATTTGAAGTTTTAACCCAGAAGCACAAGCCCGCTGAAAGCCAACAACAAGCAGCAGAAACAGAAGGCAGCTGCAACAAGAAGGACCAAAATGAGTGCAAATCCCCATGCAAATGGCATAACGATGCGGAAAACAAAAAGTGCACATTGGATAAGGAGGAGGCAAAAAAGGTAGCAGATGAGACTGCAAAAGATGGGAAAACTGGAAACACAAACACCACAGGAAGCAGCAATTCTTTTGTCATTAGCAAGACCCCTCTTTGGCTTGCAGTTTTGCTTTTTTAATTTCCCCCCTCAAATTTCCCCCCTCCTTTTAAAATTTTCCTTGCTACTTGAAAACTTTTTGATATATTTTAACACCAAAACCAGCCGAGATTTTGTGTTCTGTGTTTTGTAAGTTGACTGTCTGATTGTCTAGAAATATTTTCTGGCAACTAAAATTTTTTTCTTTTTTCCTGTTTTTTTTGTAGGTAGGTAGGAATGGGGGGGGGGGGTAGTTAGGTAGGTTAGTTAGGTTAGTTAGGGGGTTAGTTAGGGGGGTTAGGCTTAGGATTAGGATTAGACTTAGGCTTAGGATTAGGATTAGGATTAGGATTAGGGTTAATTTTTTCCTCTTTTTTTTTAACTCACACCTCTATCCTGGATTTTTAATTTTTTTTTTTAGCCATTCGCGGCTCCTTTTTTTTTTTTTGCGCCAATGTTTAATTTTTTATTGTGTTTTCAATTTTTTTGTCAACCATGCAGCGGCTGTTTTGTTATGCGGACCCTAACCCTCCTCCCCCCCCCCCGCCCGCGCACCTCCATTTTTAA

**The *TY1-VSG221* construct contained its native *VSG221* 3**′**UTR, whereas all other constructs listed above were fused to a *T. brucei* *VSG* 3’UTR. The sequences of the 3**′**UTRs are listed below.**

**The *VSG221* 3**′**UTR:**

TTTTTTTTTTTAGCCATTCGCGGCTCCTTTTTTTTTTTTTGCGCCAATGTTTAATTTTTTATTGTGTTTTCAATTTTTTTGTCAACCATGCAGCGGCTGTTTTGTTATGCGGACCCTAACCCTCCTCCCCCCCCCCCGCCCGCGCACCTCCATTTTTAAAAATTTTTTTACCGCGTCCTTCAACCAGAATTTTTTTAAATTTTTTAATTTTTTTTATTTTCCGTGGTTTTGAATCTTAATTTTTCG

**For the two *GFP* constructs from *VSG121*:**

TTTTCCCCCTCTTTTTCTTAAAAATTCTTGCTACTTGAAAACTCCTGATATATTTTAACACGCAAATTACCCGAAAAAATTGAGCTGTTTTTGTAAGTTGTAAGTTTGTGTTTCTGTGCAAATTTTTTTCTGGGAACTAGAAATTTTTTTTTTCTTTTTTCCTGTTTTTTTTAATTTTGTAGGTAGCTAGGTAGGAGG

**For all other constructs from *VSG MITat1.1*:**

TTCCCCCTCTTTTTAAACGAAATTTGCTATTTGATATATTTTAACACGTAAAAGTTTCCCGAAAAAATTGAACTTTTTTTTGTAAGTTGACTGTCTGACTGTCTAGAAATGTTTTCTGGCAGCTAAAATTTTTTTTTCTTCTTTTCCTTTTTTTTTTGTGGAAGTGGGGAGGGGGGATAGTTAGGTTAGTTAGGG
