# Supplementary material for: Endoplasmic reticulum engagement of transcripts triggers VSG mRNA balancing in trypanosomes
Source: PLoS Pathog. 2026 Aug 18;22(8):e1014518. doi: 10.1371/journal.ppat.1014518 (PMC13515020; doi:10.1371/journal.ppat.1014518)
Supplement: Supplementary file 3 [file ppat.1014518.s010.docx]

| Target | **Sequence** |
| --- | --- |
| *VSG221* | ATGCCTTCCAATCAGGAGGCCCGGCTTTTCCTCGCCGTCTTGGTCCTAGCCCAAGTTCTTCCAATTCTTGTCGATTCGGCGGCTGAAAAAGGTTTCAAACAAGCTTTTTGGCAACCTCTTTGCCAGGTCTCCGAGGAGCTAGACGACCAACCGAAGGGTGCGTTGTTTACGCTGCAAGCAGCGGCGAGCAAAATCCAGAAAATGAGGGACGCGGCACTGCGAGCAAGTATATACGCTGAAATAAATCACGGCACCAACAGGGCCAAGGCAGCCGTTATAGTCGCCAACCACTATGCCATGAAAGCTGATAGCGGCCTAGAGGCCCTAAAACAAACGTTAAGCAGCCAAGAGGTAACAGCTACTGCAACAGCGAGCTACCTAAAAGGAAGAATAGACGAATACTTAAATCTCCTTCTACAAACAAAGGAGAGCGGCACCAGCGGCTGCATGATGGACACCAGCGGAACAAACACGGTAACGAAGGCCGGCGGCACCATCGGAGGCGTTCCTTGCAAGCTGCAGTTGTCGCCGATACAGCCGAAGCGACCCGCAGCGACCTACCTAGGTAAAGCGGGCTACGTAGGCCTAACACGACAAGCAGATGCAGCCAACAATTTCCACGATAACGACGCCGAATGCAGGCTAGCCAGTGGGCACAACACCAACGGCCTCGGCAAAAGCGGCCAGCTTTCTGCAGCGGTCACTATGGCGGCCGGCTATGTCACAGTAGCGAACAGCCAAACAGCCGTCACGGTCCAGGCGCTCGATGCATTACAGGAAGCGAGCGGAGCAGCGCACCAACCGTGGATCGACGCCTGGAAGGCCAAGAAAGCGCTAACAGGAGCAGAAACCGCTGAGTTCAGAAACGAAACAGCCGGAATAGCTGGCAAAACAGGCGTTACCAAGCTTGTTGAAGAAGCTTTACTAAAGAAAAAAGACTCAGAGGCCTCAGAAATACAAACAGAATTAAAAAAATACTTTAGCGGCCACGAAAATGAACAGTGGACAGCAATAGAAAAGCTCATATCCGAGCAGCCAGTGGCGCAAAACCTGGTAGGCGACAACCAGCCAACCAAGCTAGGGGAACTGGAGGGCAATGCCAAGTTAACGACTATACTTGCCTATTACCGAATGGAAACAGCAGGGAAATTTGAAGTTTTAACCCAGAAGCACAAGCCCGCTGAAAGCCAACAACAAGCAGCAGAAACAGAAGGCAGCTGCAACAAGAAGGACCAAAATGAGTGCAAATCCCCATGCAAATGGCATAACGATGCGGAAAACAAAAAGTGCACATTGGATAAGGAGGAGGCAAAAAAGGTAGCAGATGAGACTGCAAAAGATGGGAAAACTGGAAACACAAACACCACAGGAAGCAGCAATTCTTTTGTCATTAGCAAGACCCCTCTTTGGCTTGCAGTTTTGCTTTTTTAA |
| *ILDat2.1* | ATGGATTGGGGGGGTGACAATGCGGAAAATATTGGCGCTTGCAGCGTGTGGCTGCTCTTCGCGAGGCACGCGGAGTGCGCGGAAGGCGGCAACGCAGCGGAGTTTCAGCTTCTCTGCGACGCCGCGCGACTGGCAGCAGCAATGGCAACTGGCAAGGAGCGGTCCATGCTGGCGACAACGGGGTCGCCAGAAAGCGTCGCGAGAGTGGAGGTGGGTCAAGGGTTGATTGGAGCGGCACTGGAGGCGTGGCTGAATGGGCAAAGTGACGAGGCTGATAAAAAAATCGCGGAGGAAACTGCAAGCAGGATGACGGAGGCGAGCCAGACGCACAGCGACGCATTGAAGGCCGCACTGACGGCAGCCGAGGCACACGCGCGAAGTCAAGCACAGAGCAAACAGGCAGAGGCCAAGAAACTCGCCGCACAGGCACTCGGCGTGGATGAGGGGACACTGACAATCACACAGAGCTCGACAAAGTGCGGCGAGCAGAAGTGGACAAGCAGCGCGCCAGCAAGTTACGCCACCACCCACGCGGGGAAATGCATCGCCGGCGACATCATGTACCTGTGCAAAACGCAAGCACAGAACGAATGCCTCAAAGGCCTGGTCTCAAGCAACGACATCGCCCAGAACAACGCCGTCGAAAAGTGGGAAACAGACATCAAGACACGCATCGACAAGCCATGCCTCGCCGGCGATCACACAGGCATCGAAGTAACACTCGACGCAGTGGACACAGCTATAGGGCGCATTCGCGCTGCCGTCGGCACAAAGTCAGGGAAGGACAAGTTCGGCAGTGACAGCAGCACAACGCTCACGTACACAGCAGCGTTCGCAACAAACGGCAACGGCAACGAGGGCGAACACTTCTGGGGGAAAAAACTGAAGCAGGCCGCAACGCACCTGGCCCAAGCACGCCAAGCGCTGGCATTCGCACAAGAAATGCAAGCACGCAAAAGAAAAATCGAGAACACAGAAAGGCTCGTCGGCGACCCACTGGCCGAAACAAAAACGCGACGCGCAGCCAAGGAACAAACAGCGACAGAAGCACAAAAACGGGACCATCAAGAGGGTAACGAGGACATGAGCACACGCGAGACAGGGAAGAAGGACACATCAACACGCGTGGCACCGCACCTGCTTTTGGTGGCCACCATGCTAGCTACAGCAACGGGCTGA |
| *α-Tubulin* | ATGCGTGAGGCTATCTGCATCCACATTGGTCAGGCTGGTTGCCAGGTTGGTAACGCCTGCTGGGAATTGTTCTGCCTGGAACACGGCATTCAACCCGATGGTGCGATGCCCTCTGACAAGACGATTGGCGTTGAGGATGATGCGTTCAACACCTTCTTCTCTGAGACTGGTGCTGGCAAGCACGTTCCCCGCGCGGTGTTCTTGGACCTGGAGCCAACAGTGGTGGATGAAGTGCGCACTGGCACGTACCGCCAGCTGTTCCACCCCGAGCAGCTGATCTCCGGCAAGGAGGATGCGGCCAACAACTACGCTCGTGGCCACTACACCATTGGTAAGGAGATCGTCGACCTCTGCCTGGACCGCATCCGCAAGCTCGCTGACAACTGCACTGGTCTTCAGGGCTTCCTCGTGTATCACGCCGTCGGCGGTGGCACTGGTTCTGGCCTGGGTGCGCTGCTCTTGGAGCGCCTCTCCGTGGACTATGGCAAGAAGTCCAAGCTCGGCTACACGGTGTATCCATCACCGCAGGTGTCGACGGCTGTCGTGGAGCCCTACAACTCTGTGCTCTCGACACACTCACTTCTGGAGCACACCGATGTTGCTGCGATGCTTGACAATGAAGCAATTTATGATTTGACTCGCCGCAACCTCGATATTGAGCGCCCCACGTACACCAACCTGAACCGCCTCATCGGTCAGGTGGTTTCCTCGCTGACAGCGTCCCTCCGCTTCGACGGTGCATTGAACGTGGATCTGACAGAGTTCCAGACAAACCTTGTGCCGTACCCACGTATCCACTTCGTGCTGACAAGCTATGCACCAGTCATCTCCGCAGAGAAGGCCTACCACGAGCAACTCTCTGTCTCTGAGATCTCGAACGCTGTGTTTGAGCCCGCCTCCATGATGACAAAGTGCGACCCCCGCCACGGCAAGTACATGGCGTGCTGCCTCATGTACCGTGGTGACGTTGTGCCAAAGGATGTGAATGCTGCCGTCGCGACCATCAAGACGAAGCGCACGATTCAGTTCGTGGACTGGTCTCCCACAGGCTTCAAGTGCGGTATCAACTACCAGCCACCCACGGTGGTGCCAGGTGGTGACCTTGCCAAGGTGCAGCGCGCGGTATGCATGATCGCCAACTCCACGGCCATCGCAGAGGTGTTCGCTCGTATTGACCACAAATTCGATCTCATGTACAGCAAGCGCGCCTTCGTGCACTGGTACGTCGGTGAGGGTATGGAAGAGGGTGAGTTCTCCGAGGCCCGTGAAGACCTTGCAGCACTTGAGAAGGACTACGAAGAGGTTGGTGCCGAGTCCGCGGATATGGACGGTGAGGAGGATGTGGAGGAGTACTAG |
