# Supplementary material for: Endoplasmic reticulum engagement of transcripts triggers VSG mRNA balancing in trypanosomes
Source: PLoS Pathog. 2026 Aug 18;22(8):e1014518. doi: 10.1371/journal.ppat.1014518 (PMC13515020; doi:10.1371/journal.ppat.1014518)
Supplement: Supplementary file 4 [file ppat.1014518.s011.docx]

| UniProtKB | **Name** | **Signal sequence** | **GRAVY score** |
| --- | --- | --- | --- |
| P26332 | VSG221 | MPSNQEARLFLAVLVLAQVLPILVDS | 1.054 |
| P26334 | VSG121 | MAVHRALAAYAISLYVLLPRKSGA | 0.771 |
| F9WVY4 | ILDat1.2 | MWQRILVSVGVIALVAGKARG | 1.019 |
| C9ZZN9 | BARP | MSITFHSLWLLLTVLCTAGIRA | 1.377 |
| Q57Z47 | FHR | MMISRALFVLGLSLHFIFAPTAYA | 1.438 |
| Q389V1 | EP1 | MAPRSLYLLAILLFSANLFAGVGFAAA | 1.522 |
| Q27114 | ILDat2.1 | DWGGDNAENIGACSVWLLFARHAE | -0.072 |
